# MOF-Derived Cu@N-C Catalyst for 1,3-Dipolar Cycloaddition Reaction

**DOI:** 10.3390/nano12071070

**Published:** 2022-03-24

**Authors:** Zhuangzhuang Wang, Xuehao Zhou, Shaofeng Gong, Jianwei Xie

**Affiliations:** 1 College of Chemistry and Bioengineering, Hunan University of Science and Engineering, Yongzhou 425199, China; wangzz9527@163.com; 2 Key Laboratory for Green Processing of Chemical Engineering of Xinjiang Bingtuan, School of Chemistry and Chemical Engineering, Shihezi University, Shihezi 832003, China; zxh15570943534@163.com

**Keywords:** MOF-derived materials, heterogeneous copper catalysts, 1,2,3-triazole, click chemistry

## Abstract

Cu(im)_2_-derived Cu@N-C composites were used for the first time as efficient heterogeneous catalysts for one-pot 1,3-dipolar cycloaddition of terminal alkynes, aryl halides, and sodium azide to preparation of 1,4-disubstituted 1,2,3-triazoles with broad substrate scope and high yields. The catalyst can be easily reused without the changes of structure and morphology, and the heterogeneity nature was confirmed from the catalyst recyclability and metal leaching test.

## 1. Introduction

**Figure 1:**
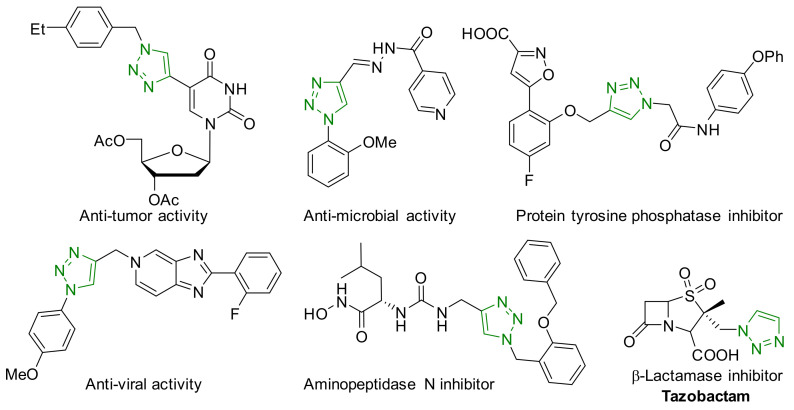
Chemical structures of some 1,2,3-triazole-containing biologically active compounds.

1,4-Disubstituted 1,2,3-triazole is a well-known structural motif present in a large class of pharmaceuticals, agrochemicals, and biologically active compounds, displaying interesting anti-tumor, anti-microbial, anti-biotics, anti-viral, and enzyme inhibitor activities (Figure 1). They are also found applications in various fields of study such as in chemistry, biochemistry, material science, medicinal chemistry, etc. [1,2,3,4,5,6,7,8,9,10,11,12,13,14,15]. For example, they are often used as versatile bio-isosteres for amide, ester, carboxylic acid, olefins, and heterocycles groups in drug discovery, chemical biology, and proteomic applications due to their suitable solubility, high chemical stability (stable to metabolic degradation and oxidative/reductive conditions) and the ability to mimic (participate in hydrogen bonding and dipole-dipole interactions) [16]. Consequently, the development of mild and efficient methods for 1,4-disubstituted 1,2,3-triazole synthesis attracts much attention in both academia and industry [17].

Cu(I)-catalyzed alkyne–azide cycloaddition reaction (CuAAC) stands out of the most used ways for the preparation of this scaffold with high selectivity and efficiency under room temperature or only moderate heating condition, which was reported independently by two groups in 2002 [18,19]. Since then, CuAAC has been extensively studied and widely used [17,20,21,22]. Because Cu(I) salts are generally unstable, traditional catalytic systems for this reaction often consist of a Cu(II) salt and a reducing agent that has been achieved significant progress in the last decades. However, the major limitations of existing protocols can be realized as using homogeneous catalysts, which are often suffered from non-recovery and non-regeneration of the catalysts, as well as difficulty in the removal of residual copper impurities from the final products. In an attempt to overcome those abovementioned drawbacks, several heterogeneous copper-based catalysts have been developed. One of the most successful strategies is to support or immobilize the copper salts or copper complexes on solid supports (such as charcoal, silica, zeolite, chitosan, montmorillonite, etc.) or into the matrix of organic and inorganic polymeric materials [20].

Metal-organic frameworks (MOFs), an essential class of porous materials composed of metal/metal clusters and polydentate organic ligands, have also been targeted as excellent heterogeneous catalyst [23,24,25,26] or catalyst supports [27] for the preparation of various 1,2,3-triazoles, regarding their elevated surface areas, tunable porous structures, and easy synthetic procedure. However, some of them are inevitably unstable in organic/water or pure water solvents due to the higher affinities of metal ions with water molecules than the organic ligands, causing the damage of MOFs structures [28]. Most of the present cycloaddition reactions often occur in aqueous environment. Thus, the development of new types of stable MOFs or MOFs-derived solid catalysts could be an effective alternative approach to address such issues.

Recently, owing to the controllable structures of MOFs, the synthesis of MOF-derived carbon-supported metal/metal oxides materials has become a fast-growing research field for their specific applications in the area of environment, energy storage, supercapacitors, and gas adsorption and separation, which have been reviewed by several groups [29,30,31,32,33,34,35,36]. Their applications in heterogeneous catalytic reactions are another important field, such as oxidation, reduction, cross-coupling, hydrogenation, and electrochemical [37,38,39,40]. These materials can be obtained via direct pyrolysis of MOFs precursors and exhibit higher tolerance against harsh conditions. However, to the best of our knowledge, only a few pieces of literature were involved in MOF-derived materials as the catalyst for click chemistry [41,42]. For example, Cu-BTC-derived copper nanocatalysts were obtained under high-pressure and high temperature (HPHT) [41] or reduction with sodium borohydride [42] and demonstrated catalytic activity in azide-alkyne Huisgen cycloaddition. As part of our studies on copper-catalyzed cycloaddition reactions [43,44], we herein report a one-pot 1,3-dipolar cycloaddition of terminal alkynes, aryl halides, and sodium azide to the preparation of 1,4-disubstituted 1,2,3-triazoles by using Cu(im)_2_-derived Cu@N-C as the efficient and recyclable heterogeneous catalyst.

## 2. Experimental

### 2.1. Material and Methods

All chemicals were purchased from commercial suppliers (*Adamas*-beta) and used without further purification. Column chromatography and thin-layer chromatography were performed with silica gel (200–300 mesh) and GF_254_ plates purchased from Qingdao Haiyang Chemical Co. Ltd. (Qingdao, China) ^1^H NMR, ^13^C NMR, and ^19^F NMR were recorded on a Bruker Avance 400 (Bruker Optics, Billerica, MA, USA) instrument using DMSO-*d*_6_ or CDCl_3_ as the solvent. All 1,4-disubstituted 1,2,3-triazoles are characterized by ^1^H NMR, ^13^C NMR, or ^19^F NMR, which were compared with the previously reported data. High-resolution mass spectrum (HRMS) was recorded on a Thermo Scientific LTQ Orbitrap XL (Walpole, MA, USA) instrument under the APCI ion source. X-ray diffraction (XRD) was measured on Bruke D8 (Karlsruhe, Germany) Advance spectrometer. Elemental analyses (EA) were performed on a Thermo Fisher Flash 2000 Elemental Analysis (Waltham, MA, USA) instrument. Inductively coupled plasma-optical emission spectroscopy (ICP-OES) was tested on Agilent 5110 (Santa Clara, CA, USA) spectrometer, and inductively coupled plasma-mass spectrometry (ICP-MS) was tested on Agilent 7800 (Santa Clara, CA, USA) spectrometer. Field emission scanning electron microscope (FE-SEM) images, elemental mapping, and EDS spectrum were recorded using on a TESCAN MIRA LMS Scanning Electron Microscope (Shanghai, China). Catalysts were calcined in a tube furnace of SLG 1200-50 (Shanghai, China).

### 2.2. Synthesis of Cu(im)_2_

The copper(II) bisimidazolate (Cu(im)_2_) was synthesized according to the reported method [45]. In a 150 mL two-necked flask, a solution of imidazole (1.36 g, 20 mmol) and NaHCO_3_ (6.6 g, 78.5 mmol) in H_2_O (50 mL) was heated to 80 °C in an oil bath for 3 h. CuSO_4_·5H_2_O (2.5 g, 10.1 mmol) in H_2_O (12.5 mL) solution was added dropwise to the imidazole/NaHCO_3_ solution while stirring. The formation of a violet precipitate was immediately observed, and then the violet compound was gradually transformed into a blue solid. After 2 h, the blue solid was filtered, washed with water, and dried at 110 °C overnight to afford Cu(im)_2_ (1.25 g, 63.5%).

### 2.3. Synthesis of Cu@N-C(x) (X Represents Different Pyrolysis Temperature)

The powder Cu(im)_2_ (1.5 g) was placed in a tube furnace and calcined up to 400 °C/600 °C/800 °C with a heating rate of 5 °C·min^−1^ under argon flow. Maintaining the targeted temperature for 5 h, the resulting solid was cooled to room temperature to afford the copper supported on nitrogen-doped carbon, denoted as Cu@N-C(400), Cu@N-C(600), and Cu@N-C(800), respectively.

### 2.4. General Procedure for the Cycloaddition Reaction

A 25 mL Schlenk tube was charged with Cu@N-C(x) (10 mg), benzyl halide (**1**, 0.5 mmol), NaN_3_ (**2**, 0.6 mmol), alkyne (**3**, 0.6 mmol) and 2 mL mixture of *t*-BuOH/water (*v*/*v* = 3:1). The mixture was stirred at 50 °C and monitored by TLC until the benzyl halide was consumed. The reaction mixture was then extracted with ethyl acetate (3 × 10 mL). The combined organic phases were washed with water and brine, dried over anhydrous Na_2_SO_4_, and concentrated in *vacuo*. The residue was purified by flash column chromatograph on silica gel (ethyl acetate/petroleum ether as the eluent) to provide the target products **4** and **5**.

### 2.5. Recycling of Cu@N-C(600) Catalyst

After complete separation of the organic phase of the reaction by centrifugation, the catalyst was washed with ethyl acetate and then dried in an oven at 100 °C for 12 h. The dried solid catalyst was reused in a new cycle and repeated the process 4 times under the standard conditions.

### 2.6. Metal Leaching Test of Cu@N-C(600) Catalyst

After completion of the reaction, the reaction mixture was filtered hot under vacuum. The solid was washed with *t*-BuOH, and the liquid phase was analyzed by ICP-MS.

## 3. Results and Discussion

**Figure 2:**
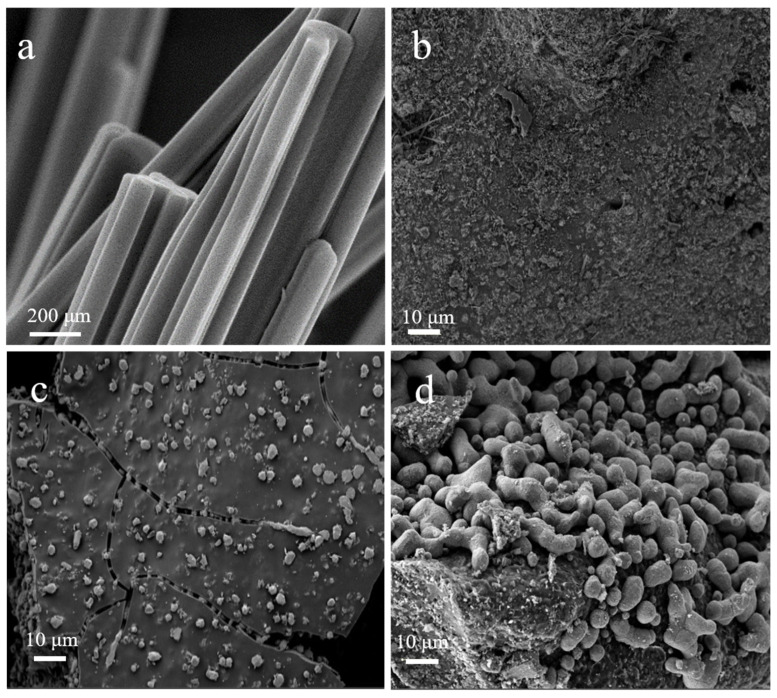
FE-SEM image of (**a**) Cu(im)_2_, (**b**) Cu@N-C(400), (**c**) Cu@N-C(600), and (**d**) Cu@N-C(800).

The powder XRD pattern (Figure S1) and FE-SEM image (Figure 2a) of the Cu(im)_2_ demonstrate that the crystal structure and morphology are all in suitable agreement with the literature [45,46,47], confirming a successful synthesis of Cu(im)_2_. Considering that Cu(im)_2_ begins to decompose when the temperature increases to ca. 300 °C, the applied calcination temperature are varied from 400 to 800 °C under argon flow [47].

**Figure 3:**
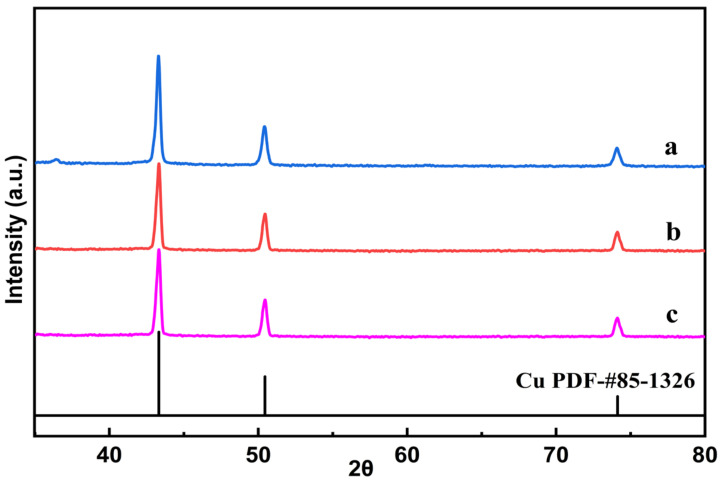
Powder XRD patterns of (**a**) Cu@N-C(400), (**b**) Cu@N-C(600), and (**c**) Cu@N-C(800).

The surface appearances of the as-synthesized Cu@N-C materials were firstly analyzed by FE-SEM analysis, and the results are shown in Figure 2. It is obviously indicated that thermolysis of Cu(im)_2_ destroyed the rod structure of the MOF template and formed a flat-like structure. Moreover, it can be seen that the copper-containing particles tended to aggregate gradually with the increase in pyrolysis temperature. Elemental mapping and EDS spectra show the distribution of C, N, and Cu present in the materials (Figures S2–S4), which further supported the FE-SEM results. In addition, we can see that N atoms homogeneously distribute in all three catalysts, suggesting the N atoms are doped successfully into the catalysts. Figure 3 shows the powder XRD pattern of the three catalysts. The XRD diffraction peaks at 43.4°, 50.5°, and 74.2° 2θ values correspond to the (111), (200) and (220) lattice planes of metallic copper (JCPDS 85-1326), respectively, which indicate that the bivalent Cu^2+^ cations in Cu(im)_2_ are in situ reduced to zero-valent Cu at high temperature. Interestingly, similar XRD patterns were observed for the three Cu@N-C composites due to the same crystal structures. These results suggested that the thermolysis temperature and N-containing imidazole ligands played important roles in particle size and species of copper.

The content of Cu was detected by ICP-OES, while the proportions of C, H, and N were determined by element analysis. As illustrated in Table 1, we can see that higher pyrolysis temperature could lead to a significant increase in the Cu’s content, and about 73.73% of Cu content is observed when the temperature rises to 800 °C. On the contrary, the contents of C, N, and H decreased. In addition, the actual measured value of Cu(im)_2_ is slightly lower than the theoretical calculated value, which may be related to the existence of a few impurities.

With the Cu@N-C composites prepared, the cycloaddition reaction between benzyl bromide (**1a**), sodium azide (**2**), and phenylacetylene (**3a**) was selected as the model reaction to investigate the catalytic activity. The results are summarized in Table 2. The parent Cu(im)_2_ only gave low activity, suggesting that cupric ions coordinated with imidazole were not suitable for this reaction (entry 1). The reaction could not proceed without copper catalyst (entry 2). To our delight, Cu(im)_2_ pyrolyzed composites showed high activities in this transformation, and Cu@N-C(600) exhibited the highest efficiency, affording the corresponding 1,2,3-triazole **4a** in 94% isolated yield (entries 3–5). Lower or higher thermolysis temperature all resulted in lower reactivity of the resulting materials. Investigation of a variety of solvents (H_2_O, alcohols, or H_2_O/alcohols mixture) showed that the mixed solvents of *t*-BuOH/H_2_O (3/1, *v*/*v*) gave a better yield (98%) than others (entries 6–17). Inferior results were found when reducing the catalyst loading or lowering the reaction temperature (entries 18–20). As the classical catalytic system, the mixture of CuSO_4_ and sodium ascorbate (NaAsc), which was used as the catalyst under the same reaction conditions, also resulted in obviously decreased yield (entry 21).

Having established the optimized conditions (Table 2, entry 15), we then evaluated the substrates scope of alkynes and aryl halides, and the results were listed in Table 3. For alkynes, we found that all arylacetylenes bearing electron-donating, electron-withdrawing, or electron-neutral groups at the *para*-, *meta*-, or *ortho*-positions of the aromatic ring could smoothly be converted the desired triazoles in 90–98% yield (**4a**–**4k**). Sterically 2-substituted substrate also reacted without any problem to give **4f** in 90% yield. Aliphatic alkynes seemed to be less reactive than aryl alkynes, and longer reaction times or higher temperatures were needed (**4l** and **4m**). For benzyl bromides, most substrates worked well to give the corresponding cycloaddition products in 70–95% yields under the standard conditions (**4n**–**4ae**). However, the electron effect of substituents was more obvious than the aryl acetylenes. For example, the substrates containing electron-withdrawing groups at the *para*-condition resulted in lower yields than other ones (**4s**–**4v**). Longer reaction time was required for benzyl chloride due to the lower activity for the nucleophilic substitution reaction between sodium azide and benzyl chloride, which is the first step for the one-pot 1,3-dipolar cycloaddition (**4a** and **4s**).

**Figure 4:**
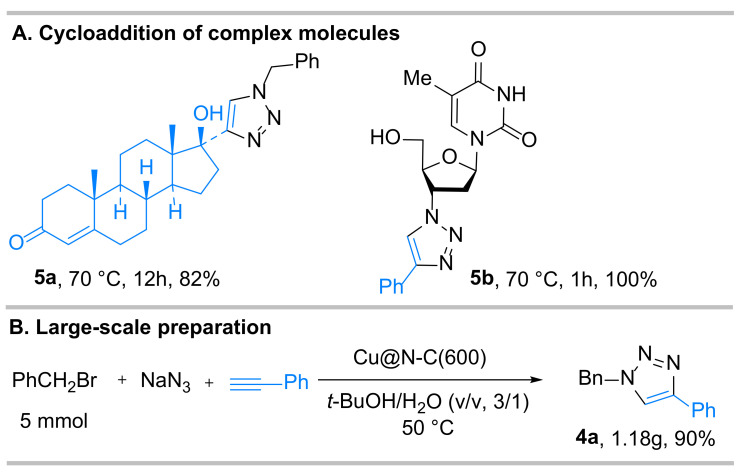
Synthetic applications and large-scale preparation.

Based on the heterogeneous catalyst and mild reaction conditions, as well as excellent functional-group compatibility, the catalytic system was also used to derivatize two selected complex drug-like substrates bearing alkyne or azide moieties (Figure 4). Ethisterone showed suitable efficiency, and the corresponding cycloaddition product was obtained in 82% yield with only increasing the temperature to 70 °C (**5a**). Surprisingly, a quantitative yield was gained for zidovudine within 1 h (**5b**). Furthermore, in order to establish the industrial viability of our method, a gram-scale reaction was carried out under the optimal reaction conditions, and the expected product **4a** was formed in 90% yield (1.18 g), indicating the synthetic utility of this method from a practical point of view.

**Figure 5:**
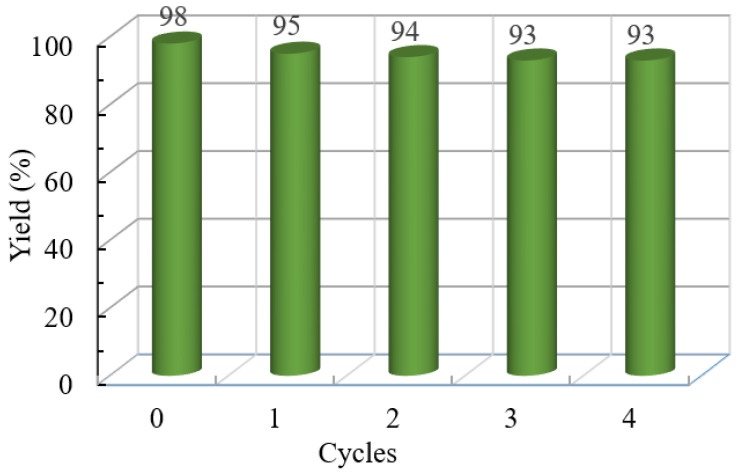
Reuse of Cu@N-C(600) catalyst.

**Figure 6:**
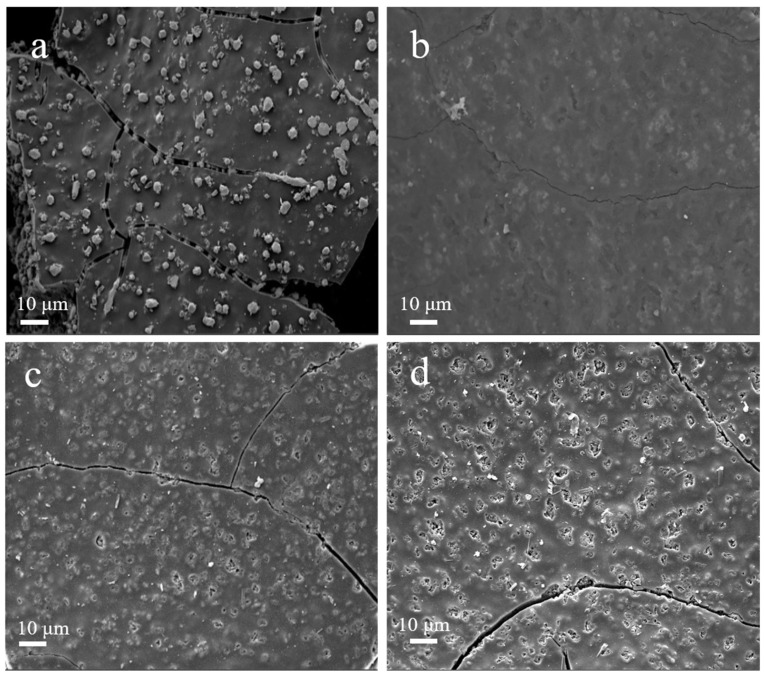
FE-SEM image of (**a**) Cu@N-C(600), and used Cu@N-C(600) (**b**) the first run, (**c**) the second run, and (**d**) the fourth run.

Recoverability and reusability of Cu@N-C(600) were investigated in the cycloaddition between benzyl bromide, NaN_3,_ and phenylacetylene. According to Figure 5, the recycled catalyst Cu@N-C(600) could be recovered and reused without considerable deterioration in catalytic activities, and the yield of **4a** always remains above 90% after four runs. The XRD patterns of the recycled catalyst showed no obvious differences of the fresh Cu@N-C(600), which indicates that the crystallinity and structure of the catalyst can be maintained well during the process of the reaction (Figure S5). However, different results were observed from the FE-SEM image of the reused catalysts (Figure 6). The metal particles on the surface of fresh Cu@N-C(600) disappeared after the first run, but no changes in the second and the fourth run. The loss of surface copper species may result from the weak binding affinity and mechanical abrasion-induced exfoliation during the reaction process. The results were also supported by the EDS and element mapping analysis. As illustrated in Figures S6–S8, the copper element mapping of the first reused catalyst showed a weaker signal than the fresh one, but the second and fourth cycles were basically unchanged from the first one.

**Figure 7:**
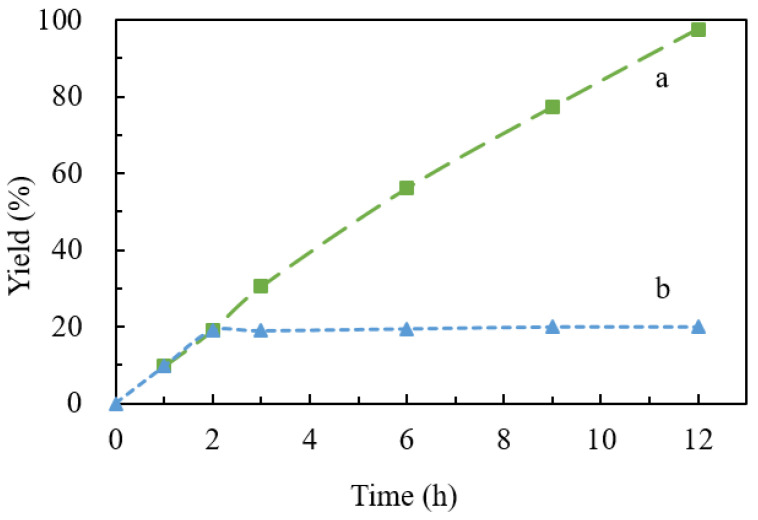
Time-concentration profile of the model reaction and the yield was determined by ^1^H NMR with 1,3,5-trimethoxybenzene as the standard. (**a**) Reaction under the optimized conditions; (**b**) reaction after removal of Cu@N-C(600) catalyst by hot filtration at 2 h.

Leaching experiments for model reactions between benzyl bromide, sodium azide, and phenylacetylene were conducted to check the stability of the catalyst. We can see that 1.7% of the initial copper content was detected in the reaction solution by ICP-MS analysis, which was collected by hot filtration after the first cycle. Lower Cu leaching was observed in the next three cycles (Table 4), which was in suitable agreement with the FE-SEM results. Moreover, the reaction with the solution after removal of catalyst via hot filtration at approximately 20% yield stopped, and the yield of cycloaddition product did not increase further even after 10 h under the same conditions (Figure 7). These aforementioned results suggested that Cu(im)_2_-derived Cu@N-C composites was an excellent stable and reusable heterogeneous catalyst for this type of reaction.

Finally, we compared the activity of the present MOF-derived catalyst with other reported heterogeneous copper catalysts in the one-pot 1,3-dipolar cycloaddition reaction (Table S1). The results demonstrated that the present catalyst exhibited a higher efficiency with higher yields, mild reaction conditions, and broad substrate scopes than other reported methods.

## 4. Conclusions

In summary, by employing a MOF-templated method, we have reported a Cu(im)_2_-derived Cu@N-C composite, which was applied for the first time as an efficient, recyclable heterogeneous catalyst for the synthesis of 1,4-disubstituted 1,2,3-triazoles with high yields. The catalyst features easily prepared, broad substrate scope with excellent functional tolerance and regioselectivity, low metal leaching, and ambient reaction conditions. The catalyst can be separated by simple filtration and recovered at least four times without declining activity, and the structure maintained well during the reaction process. Further investigations into other types of Cu@N-C-catalyzed reactions are currently ongoing in our laboratory and will be reported in due course.

## Figures and Tables

**Table 1:** The contents of Cu, C, N, and H of samples.

Sample	Cu Content (%) ^1^	C Content (%) ^2^	N Content (%) ^2^	H Content (%) ^2^
Cu(im)_2_	32.14 ^3^	36.45 ^3^	28.34 ^3^	3.06 ^3^
Cu(im)_2_	29.20	35.49	27.89	2.70
Cu@N-C(400)	39.03	22.76	16.29	1.30
Cu@N-C(600)	48.85	23.98	14.48	0.86
Cu@N-C(800)	73.73	12.58	1.31	0.24

^1^ Measured by ICP-OES; ^2^ measured by element analysis; ^3^ calculated value from the formula.

**Table 2:**
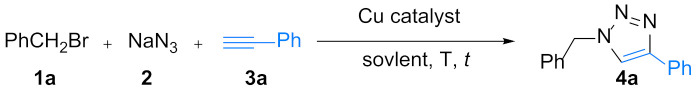
Optimization of the reaction conditions ^1^.

Entry	Catalyst	Solvent (*v*/*v*)	T (°C)	Yield (%) ^2^
1	Cu(im)_2_	H_2_O	50	20
2	-	H_2_O	50	trace
3	Cu@N-C(400)	H_2_O	50	83
4	Cu@N-C(600)	H_2_O	50	94
5	Cu@N-C(800)	H_2_O	50	78
6	Cu@N-C(600)	EtOH	50	90
7	Cu@N-C(600)	*i*-PrOH	50	68
8	Cu@N-C(600)	*t*-BuOH	50	7
9	Cu@N-C(600)	EtOH/H_2_O (3/1)	50	96
10	Cu@N-C(600)	EtOH/H_2_O (1/3)	50	88
11	Cu@N-C(600)	EtOH/H_2_O (1/1)	50	40
12	Cu@N-C(600)	*i*-PrOH/H_2_O (3/1)	50	92
13	Cu@N-C(600)	*i*-PrOH/H_2_O (1/3)	50	86
14	Cu@N-C(600)	*i*-PrOH/H_2_O (1/1)	50	74
15	Cu@N-C(600)	*t*-BuOH/H_2_O (3/1)	50	98
16	Cu@N-C(600)	*t*-BuOH/H_2_O (1/3)	50	45
17	Cu@N-C(600)	*t*-BuOH/H_2_O (1/1)	50	55
18	Cu@N-C(600)	*t*-BuOH/H_2_O (3/1)	50	80 ^3^
19	Cu@N-C(600)	*t*-BuOH/H_2_O (3/1)	40	56
20	Cu@N-C(600)	*t*-BuOH/H_2_O (3/1)	25	50
21	CuSO_4_ + NaAsc	*t*-BuOH/H_2_O (3/1)	50	88 ^4^

^1^ Reaction conditions: **1a** (0.5 mmol), **2a** (0.6 mmol), **3a** (0.6 mmol), Cu catalyst (10 mg), solvent (2 mL), 12 h; ^2^ isolated yield; ^3^ catalyst (5 mg); ^4^ CuSO_4_ (5 mol%), NaAsc (10 mmol%).

**Table 3:**
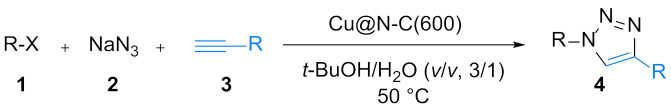
Investigation of substrate scopes and limitations ^1^.

Scope of alkynes (14 examples)		Scope of benzyl halides (19 examples)	
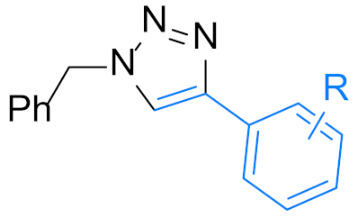		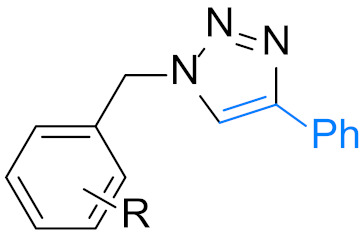	
**4a**: R=H	12 h, 98%	**4n**: R = 4-Me	12 h, 86%
	24 h, 90% (X=Cl)	**4o**: R = 3,5-di-Me	12 h, 88%
**4b**: R = 4-Me	12 h, 90%	**4p**: R = 3-OMe	24 h, 88%
**4c**: R = 4-Et	24 h, 93%	**4q**: R = 3,5-di-OMe	24 h, 82%
**4d**: R = 4-OMe	12 h, 90%	**4r**: R = 4-*t*-Bu	14 h, 87%
**4e**: R = 3-Me	12 h, 92% ^2^	**4s**: R = 4-F	12 h, 80%
**4f**: R = 2-Me	12 h, 90%		24 h, 94% (X=Cl)
**4g**: R = 4-F	12 h, 97%	**4t**: R = 4-Cl	24 h, 72%
**4h**: R = 4-Cl	24 h, 91%	**4u**: R = 4-Br	24 h, 70%
**4i**: R = 4-Br	24 h, 90%	**4v**: R = 4-NO_2_	12 h, 85% ^2^
**4j**: R = 3-F	12 h, 93% ^2^	**4w**: R = 4-CF_3_	15 h, 90%
**4k**: R = 3-Br	24 h, 91% ^2^	**4x**: R = 2-F	24 h, 94%
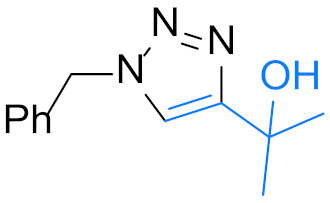	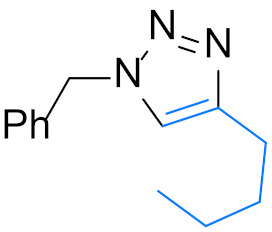	**4y**: R = 2-Cl	24 h, 95%
		**4z**: R = 2,5-di-F	24 h, 82%
		**4aa**: R = 3-F	16 h, 90%
		**4ab**: R = 3-Cl	24 h, 94%
		**4ac**: R = 3-Br	15 h, 90% ^2^
**4l**, 12 h, 97% ^2^	**4m**, 18 h, 66% ^2^	**4ad**: R = 3,4-di-Cl	12 h, 76%
		**4ae**: R = 3-Cl, 4-F	24 h, 94%

^1^ Reaction conditions: **1** (0.5 mmol), **2** (0.6 mmol), **3** (0.6 mmol), Cu@N-C(600) (10 mg), *t*-BuOH/H_2_O (2 mL), 50 °C, isolated yield; ^2^ 60 °C.

**Table 4:** The Cu leaching of Cu@N-C(600) catalyst.

Sample	Cu Leaching (%) ^1^
First run	1.7
Second run	0.5
Third run	0.4
Fourth run	0.5

^1^ Measured by ICP-MS analysis.

## Data Availability

The data are available on reasonable request from the corresponding author.

## Supplementary Materials

The following supporting information can be downloaded at: https://www.mdpi.com/article/10.3390/nano12071070/s1, Figure S1: Powder XRD patterns of Cu(im)_2_, Figure S2: (a) FE-SEM image, elemental mapping of (b) C, (c) N, and (d) Cu, and (e) EDS spectrum of Cu@N-C(400), Figure S3: (a) FE-SEM image, elemental mapping of (b) C, (c) N, and (d) Cu, and (e) EDS spectrum of Cu@N-C(600), Figure S4: (a) FE-SEM image, elemental mapping of (b) C, (c) N, and (d) Cu, and (e) EDS spectrum of Cu@N-C(800), Figure S5: Powder XRD pattern of (a) fresh and (b) used Cu@N-C(600), Figure S6: (a) FE-SEM image, elemental mapping of (b) C, (c) N, and (d) Cu, and (e) EDS spectrum of the used Cu@N-C(600) (the first run), Figure S7: (a) FE-SEM image, elemental mapping of (b) C, (c) N, and (d) Cu, and (e) EDS spectrum of the used Cu@N-C(600) (the second run), Figure S8: (a) FE-SEM image, elemental mapping of (b) C, (c) N, and (d) Cu, and (e) EDS spectrum of the used Cu@N-C(600) (the fourth run); characterization data of compound **4a**–**5b**, Table S1: Comparison of catalytic performance between Cu@N-C(600) and other types of catalysts. References [48,49,50,51,52,53,54,55,56,57,58,59,60] are cited in the supplementary materials.

## References

[1] Kolb H.C., Finn M.G., Sharpless K.B. (2001). Click chemistry: Diverse chemical function from a few good reactions. Angew. Chem. Int. Ed..

[2] Tron G.C., Pirali T., Billington R.A., Canonico P.L., Sorba G., Genazzani A.A. (2008). Click chemistry reactions in medicinal chemistry: Applications of the 1,3-dipolar cycloaddition between azides and alkynes. Med. Res. Rev..

[3] Thirumurugan P., Matosiuk D., Jozwiak K. (2013). Click chemistry for drug development and diverse chemical-biology applications. Chem. Rev..

[4] Tang W., Becker M.L. (2014). “Click” reactions: A versatile toolbox for the synthesis of peptide-conjugates. Chem. Soc. Rev..

[5] Xi W., Scott T.F., Kloxin C.J., Bowman C.N. (2014). Click chemistry in materials science. Adv. Funct. Mater..

[6] Lal K., Yadav P. (2018). Recent advancements in 1,4-disubstituted 1*H*-1,2,3-triazoles as potential anticancer agents. Anti-Cancer Agents Med. Chem..

[7] Lin S., Sharma A. (2018). Recent advances in the synthesis and synthetic applications of 1,2,3-triazoles. Chem. Heterocycl. Compd..

[8] Rani A., Singh G., Singh A., Maqbool U., Kaur G., Singh J. (2020). CuAAC-ensembled 1,2,3-triazole-linked isosteres as pharmacophores in drug discovery: Review. RSC Adv..

[9] Recnik L.-M., Mindt T.L., Recnik L.-M., Kandioller W., Mindt T.L., Recnik L.-M., Mindt T.L. (2020). 1,4-Disubstituted 1,2,3-triazoles as amide bond surrogates for the stabilisation of linear peptides with biological activity. Molecules.

[10] Sahu A., Sahu P., Agrawal R. (2020). A recent review on drug modification using 1,2,3-triazole. Curr. Chem. Biol..

[11] Slavova K.I., Todorov L.T., Kostova I.P., Belskaya N.P., Palafox M.A. (2020). Developments in the application of 1,2,3-triazoles in cancer treatment. Recent Pat. Anticancer Drug Discov..

[12] Agouram N., Mestafa El Hadrami E., Bentama A. (2021). 1,2,3-Triazoles as biomimetics in peptide science. Molecules.

[13] Wang X., Zhang X., Ding S. (2021). 1,2,3-Triazole-based sequence-defined oligomers and polymers. Poly. Chem..

[14] Da S.M., Forezi L., Lima C.G.S., Amaral A.A.P., Ferreira P.G., de Souza M.C.B.V., Cunha A.C., da Silva D.C.F., Ferreira V.F. (2021). Bioactive 1,2,3-triazoles: An account on their synthesis, structural diversity and biological applications. Chem. Rec..

[15] Bozorov K., Zhao J., Aisa H.A. (2019). 1,2,3-Triazole-containing hybrids as leads in medicinal chemistry: A recent overview. Bioorg. Med. Chem..

[16] Bonandi E., Christodoulou M.S., Fumagalli G., Perdicchia D., Rastelli G., Passarella D. (2017). The 1,2,3-triazole ring as a bioisostere in medicinal chemistry. Drug Discov. Today.

[17] Forezi D.S.M.L., Cardoso F.C.M., Gonzaga T.G.D., da Silva D.C.F., Ferreira V.F. (2018). Alternative routes to the click method for the synthesis of 1,2,3-triazoles, an important heterocycle in medicinal chemistry. Curr. Top. Med. Chem..

[18] Rostovtsev V.V., Green L.G., Fokin V.V., Sharpliess K.B. (2002). A stepwise huisgen cycloaddition process: Copper(I)-catalyzed regioselective “ligation” of azides and terminal alkynes. Angew. Chem. Int. Ed..

[19] Tornøe C.W., Christensen C., Meldal M. (2002). Peptidotriazoles on solid phase: [1,2,3]-triazoles by regiospecific copper(I)-catalyzed 1,3-dipolar cycloadditions of terminal alkynes to azides. J. Org. Chem..

[20] Noriega S., Leyva E., Moctezuma E., Flores L., Loredo-Carrillo S. (2020). Recent catalysts used in the synthesis of 1,4-disubstituted 1,2,3-triazoles by heterogeneous and homogeneous methods. Curr. Org. Chem..

[21] Kaushik C.P., Sangwan J., Luxmi R., Kumar K., Pahwa A. (2019). Synthetic routes for 1,4-disubstituted 1,2,3-triazoles: A review. Curr. Org. Chem..

[22] Meldal M., Tornoe C.W. (2008). Cu-catalyzed azide-aldyne cycloaddition. Chem. Rev..

[23] Jia X., Xu G., Du Z., Fu Y. (2018). Cu(BTC)-MOF catalyzed multicomponent reaction to construct 1,4-disubstituted-1,2,3-triazoles. Polyhedron.

[24] Luz I., Llabrés i Xamena F.X., Corma A. (2010). Bridging homogeneous and heterogeneous catalysis with MOFs: “Click” reactions with Cu-MOF catalysts. J. Catal..

[25] Li P., Regati S., Huang H., Arman H.D., Zhao J.C.G., Chen B. (2015). A metal–organic framework as a highly efficient and reusable catalyst for the solvent-free 1,3-dipolar cycloaddition of organic azides to alkynes. Inorg. Chem. Front..

[26] Mansano Willig J.C., Granetto G., Reginato D., Dutra F.R., Poruczinski É.F., de Oliveira I.M., Stefani H.A., de Campos S.D., de Campos É.A., Manarin F. (2020). A comparative study between Cu(INA)_2_-MOF and [Cu(INA)_2_(H_2_O)_4_] complex for a click reaction and the Biginelli reaction under solvent-free conditions. RSC Adv..

[27] Hu Y.-H., Wang J.-C., Yang S., Li Y.-A., Dong Y.-B. (2017). CuI@UiO-67-IM: A MOF-based bifunctional composite triphase-transfer catalyst for sequential one-pot azide–alkyne cycloaddition in water. Inorg. Chem..

[28] Liu J., Chen L., Cui H., Zhang J., Zhang L., Su C.Y. (2014). Applications of metal-organic frameworks in heterogeneous supramolecular catalysis. Chem. Soc. Rev..

[29] Marpaung F., Kim M., Khan J.H., Konstantinov K., Yamauchi Y., Hossain M.S.A., Na J., Kim J. (2019). Metal–organic framework (MOF)-derived nanoporous carbon materials. Chem. Asian J..

[30] Han A., Wang B., Kumar A., Qin Y., Jin J., Wang X., Yang C., Dong B., Jia Y., Liu J. (2019). Recent advances for MOF-derived carbon-supported single-atom catalysts. Small Methods.

[31] Zhou Q., Wu Y. (2018). Research progress on prepartion of MOF-derived porous carbon materials through pyrolysis. Chin. Sci. Bull..

[32] Cao X., Tan C., Sindoro M., Zhang H. (2017). Hybrid micro-/nano-structures derived from metal–organic frameworks: Preparation and applications in energy storage and conversion. Chem. Soc. Rev..

[33] Pei X., Chen Y., Li S., Zhang S., Feng X., Zhou J., Wang B. (2016). Metal-organic frameworks derived porous carbons: Syntheses, porosity and gas sorption properties. Chin. J. Chem..

[34] Xia W., Mahmood A., Zou R., Xu Q. (2015). Metal–organic frameworks and their derived nanostructures for electrochemical energy storage and conversion. Energ. Environ. Sci..

[35] Chaikittisilp W., Ariga K., Yamauchi Y. (2013). A new family of carbon materials: Synthesis of MOF-derived nanoporous carbons and their promising applications. J. Mater. Chem. A.

[36] Zhao Y., Song Z., Li X., Sun Q., Cheng N., Lawes S., Sun X. (2016). Metal organic frameworks for energy storage and conversion. Energy Storage Mater..

[37] Shen K., Chen X., Chen J., Li Y. (2016). Development of MOF-derived carbon-based nanomaterials for efficient catalysis. ACS Catal..

[38] Hu L., Li W., Wang L., Wang B. (2021). Turning metal-organic frameworks into efficient single-atom catalysts via pyrolysis with a focus on oxygen reduction reaction catalysts. EnergyChem.

[39] Konnerth H., Matsagar B.M., Chen S.S., Prechtl M.H.G., Shieh F.-K., Wu K.C.W. (2020). Metal-organic framework (MOF)-derived catalysts for fine chemical production. Coordin. Chem. Rev..

[40] Chen Y.-Z., Zhang R., Jiao L., Jiang H.-L. (2018). Metal–organic framework-derived porous materials for catalysis. Coordin. Chem. Rev..

[41] Yamane I., Sato K., Otomo R., Yanase T., Miura A., Nagahama T., Kamiya Y., Shimada T. (2021). Ultrahigh-pressure preparation and catalytic activity of MOF-derived Cu nanoparticles. Nanomaterials.

[42] Kim A., Muthuchamy N., Yoon C., Joo S.H., Park K.H. (2018). MOF-derived Cu@Cu_2_O nanocatalyst for oxygen reduction reaction and cycloaddition reaction. Nanomaterials.

[43] Zhou C., Zhang J., Liu P., Xie J., Dai B. (2015). 2-Pyrrolecarbaldiminato-Cu(II) complex catalyzed three-component 1,3-dipolar cycloaddition for 1,4-disubstituted 1,2,3-triazoles synthesis in water at room temperature. RSC Adv..

[44] Hao C., Zhou C., Xie J., Zhang J., Liu P., Dai B. (2015). An Efficient Copper-catalyzed one-pot synthesis of 1-aryl-1,2,3-triazoles from arylboronic acids in water under mild conditions. Chin. J. Chem..

[45] Masciocchi N., Bruni S., Cariati E., Cariati F., Galli S., Sironi A. (2001). Extended polymorphism in copper(II) imidazolate polymers:  a spectroscopic and XRPD structural study. Inorg. Chem..

[46] Luz I., Corma A., Llabrés i Xamena F.X. (2014). Cu-MOFs as active, selective and reusable catalysts for oxidative C–O bond coupling reactions by direct C–H activation of formamides, aldehydes and ethers. Catal. Sci. Technol..

[47] Karahan Ö., Biçer E., Taşdemir A., Yürüm A., Gürsel S.A. (2018). Development of efficient copper-based MOF-derived catalysts for the reduction of aromatic nitro compounds. Eur. J. Inorg. Chem..

[48] Bonyasi R., Gholinejad M., Saadati F., Nájera C. (2018). Copper ferrite nanoparticle modified starch as a highly recoverable catalyst for room temperature click chemistry: Multicomponent synthesis of 1,2,3-triazoles in water. New J. Chem..

[49] Khalili D., Kavoosi L., Khalafi-Nezhad A. (2019). Copper aluminate spinel in click chemistry: An efficient heterogeneous nanocatalyst for the highly regioselective synthesis of triazoles in water. Synlett.

[50] Hasanpour Z., Maleki A., Hosseini M., Gorgannezhad L., Nejadshafiee V., Ramazani A., Haririan I., Shafiee A., Khoobi M. (2017). Efficient multicomponent synthesis of 1,2,3-triazoles catalyzed by Cu(II) supported on PEI[Rate]Fe_3_O_4_ MNPs in a water/PEG300 system. Turk. J. Chem..

[51] Karimi Zarchi M.A., Nazem F. (2014). One-pot three-component synthesis of 1,4-disubstituted 1*H*-1,2,3-triazoles using green and recyclable cross-linked poly(4-vinylpyridine)-supported copper sulfate/sodium ascorbate in water/t-BuOH system. J. Iran. Chem. Soc..

[52] Haito A., Yamaguchi M., Chatani N. (2018). Ru_3_(CO)_12_-catalyzed carbonylation of C−H bonds by triazole-directed C−H activation. Asian J. Org. Chem..

[53] Zheng Z., Shi L. (2016). An efficient regioselective copper-catalyzed approach to the synthesis of 1,2,3-triazoles from N-tosylhydrazones and azides. Tetrahedron Lett..

[54] Dehbanipour Z., Moghadam M., Tangestaninejad S., Mirkhani V., Mohammadpoor-Baltork I. (2017). Copper(II) bisthiazole complex immobilized on silica nanoparticles: Preparation, characterization and its application as a highly efficient catalyst for click synthesis of 1,2,3-triazoles. Polyhedron.

[55] Zhao Z., Wang X., Si J., Yue C., Xia C., Li F. (2018). Truncated concave octahedral Cu_2_O nanocrystals with {hkk} high-index facets for enhanced activity and stability in heterogeneous catalytic azide–alkyne cycloaddition. Green Chem..

[56] Pérez J.M., Cano R., Ramón D.J. (2014). Multicomponent azide–alkyne cycloaddition catalyzed by impregnated bimetallic nickel and copper on magnetite. RSC Adv..

[57] Larionov V.A., Stashneva A.R., Titov A.A., Lisov A.A., Medvedev M.G., Smol’yakov A.F., Tsedilin A.M., Shubina E.S., Maleev V.I. (2020). Mechanistic study in azide–alkyne cycloaddition (CuAAC) catalyzed by bifunctional trinuclear copper(I) pyrazolate complex: Shift in rate-determining step. J. Catal..

[58] De Angelis S., Franco M., Trimini A., Gonzalez A., Sainz R., Degennaro L., Romanazzi G., Carlucci C., Petrelli V., de la Esperanza A. (2019). A study of graphene-based copper catalysts: Copper(I) nanoplatelets for batch and continuous-flow applications. Chem. Asian J..

[59] Rajabi M., Albadi J., Momeni A. (2020). Click synthesis of 1,4-disubstituted-1,2,3-triazoles catalyzed by melamine-supported CuO nanoparticles as an efficient recyclable catalyst in water. Res. Chem. Intermediat..

[60] Ötvös S.B., Georgiádes Á., Ádok-Sipiczki M., Mészáros R., Pálinkó I., Sipos P., Fülöp F. (2015). A layered double hydroxide, a synthetically useful heterogeneous catalyst for azide−alkyne cycloadditions in a continuous-flow reactor. Appl. Catal. A: Gen..

