# Supplementary material for: MOF-Derived Cu@N-C Catalyst for 1,3-Dipolar Cycloaddition Reaction
Source: Nanomaterials (Basel). 2022 Mar 24;12(7):1070. doi: 10.3390/nano12071070 (PMC9000828; doi:10.3390/nano12071070)

nanomaterials

Supporting Information

MOF-Derived Cu@N-C Catalyst for 1,3-Dipolar Cycloaddition Reaction

Zhuangzhuang Wang [1,2], Xuehao Zhou [2], Shaofeng Gong [1,*] and Jianwei Xie [1,2,*]

1 College of Chemistry and Bioengineering, Hunan University of Science and Engineering, Yongzhou 425199, China; wangzz9527@163.com
2 School of Chemistry and Chemical Engineering/Key Laboratory for Green Processing of Chemical Engineering of Xinjiang Bingtuan, Shihezi University, Shihezi 832003, China; zxh15570943534@163.com
* Correspondence: simon.gong@huse.edu.cn (S.G.); cesxjw@foxmail.com (J.X.)

Characterization of the catalyst. 3

Table S1. Comparison of catalytic performance between Cu@N-C(600) and other types of catalysts. 8

Characterization data of compound **4a-5b** 9

References.. 20

Copies of 1H NMR, 13C NMR and 19F NMR spectra 22

Citation: Wang, Z.; Zhou, X.; Gong, S.; Xie, J. MOF-Derived Cu@N-C Catalyst for 1,3-Dipolar Cycloaddition Reaction. *Nanomaterials* **2022**, *12*, 1070. https://doi.org/10.3390/nano12071070

Academic Editor: Kyoung-Su Ha

Publisher's Note: MDPI stays neutral with regard to jurisdictional claims in published maps and institutional affiliations.

Copyright: © 2022 by the authors. Submitted for possible open access publication under the terms and conditions of the Creative Commons Attribution (CC BY) license (https://creativecommons.org/licenses/by/4.0/).

Characterization of the catalyst.

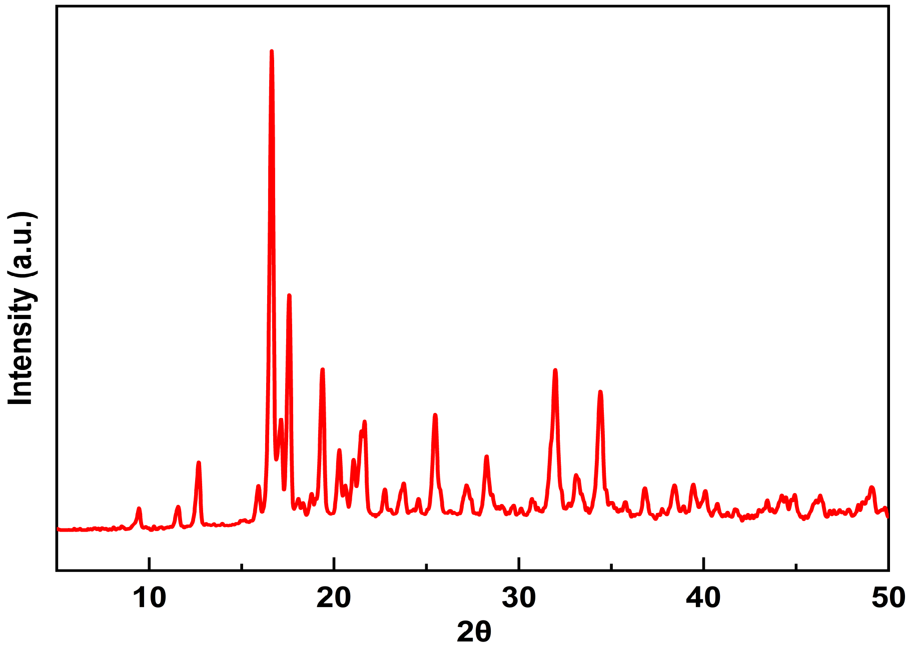

Figure S1. Powder XRD patterns of Cu(im)$_2$.

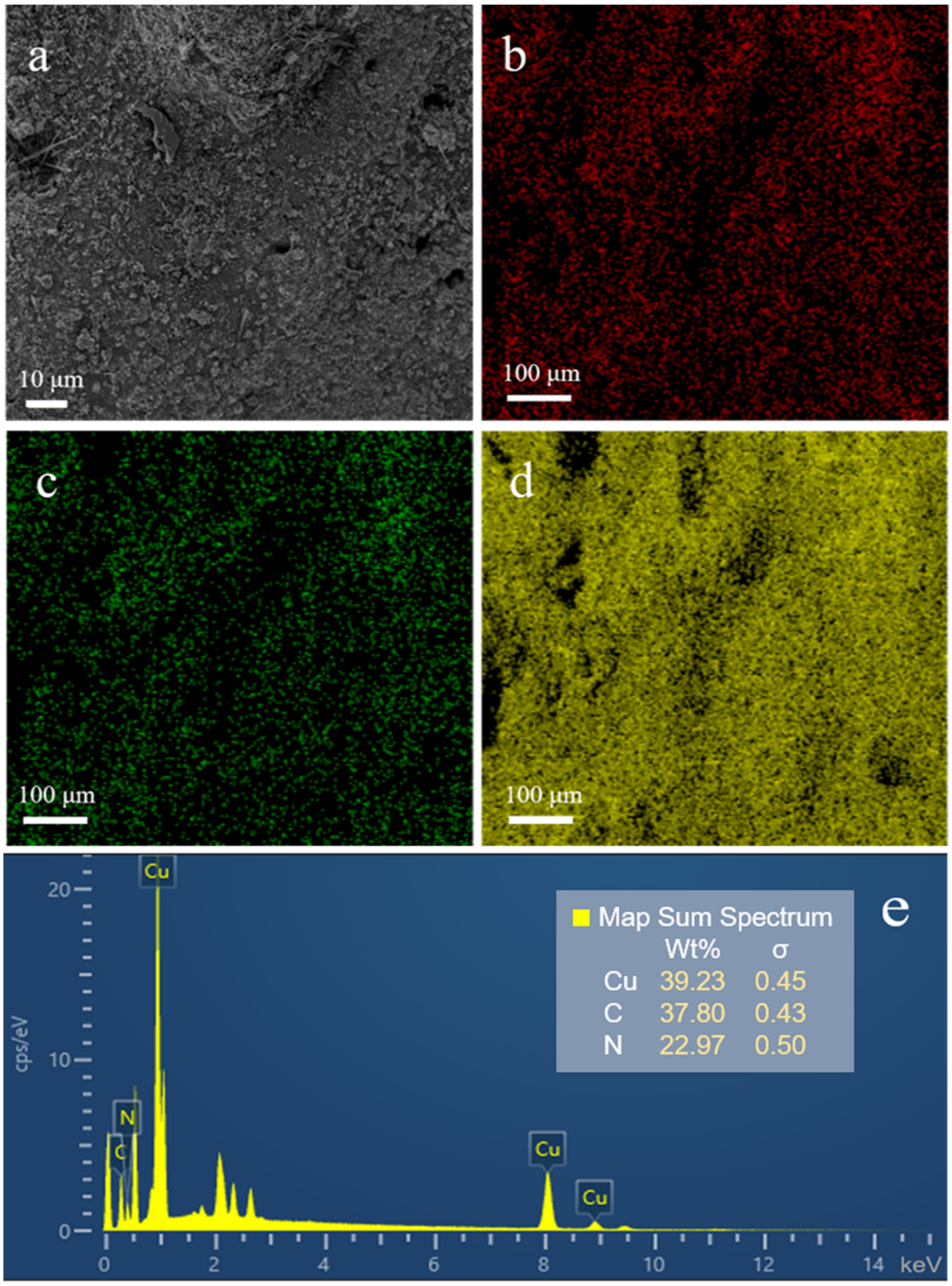

Figure S2. (a) FE-SEM image, elemental mapping of (b) C, (c) N, and (d) Cu, and (e) EDS spectrum of Cu@N-C(400).

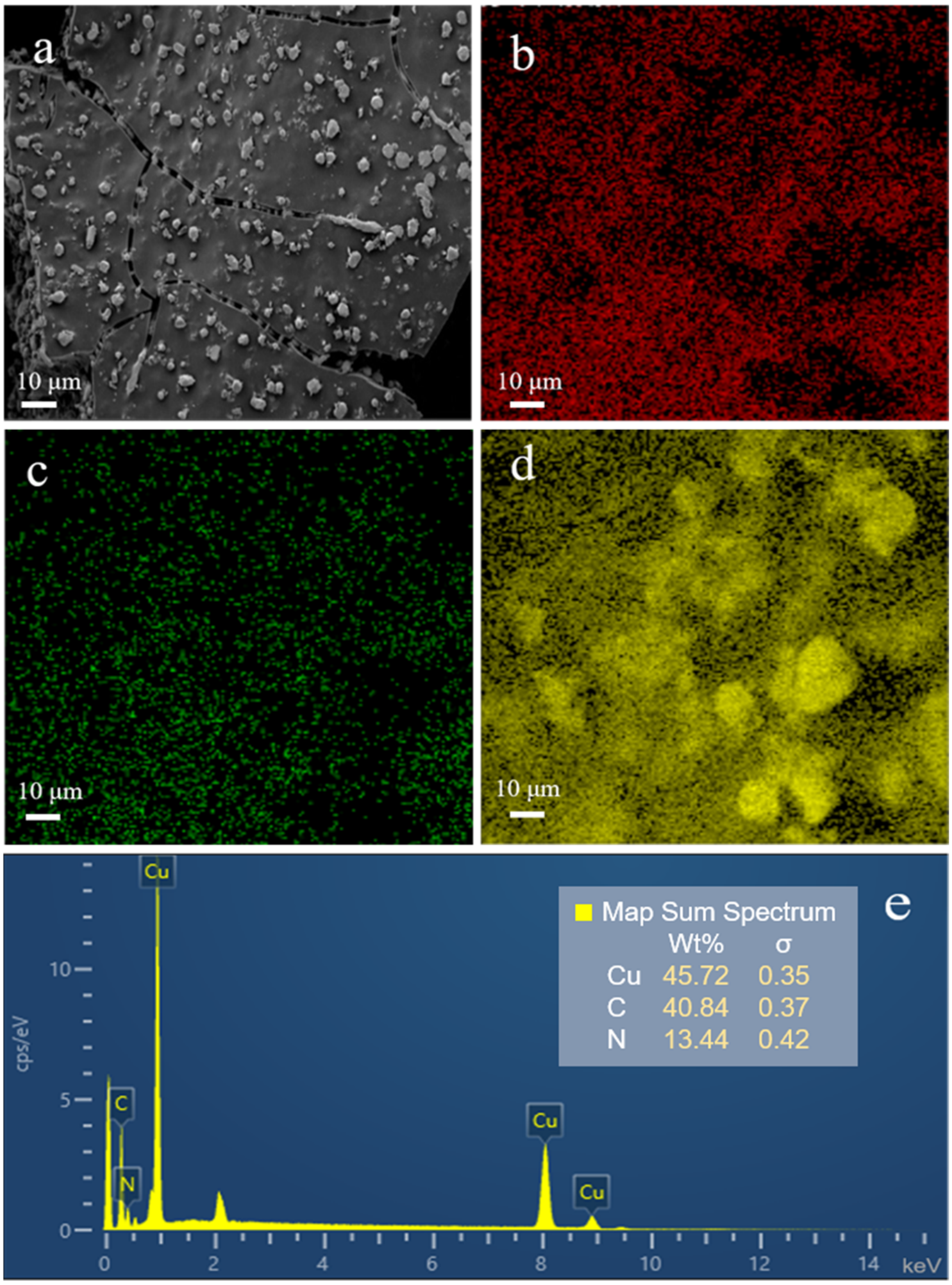

Figure S3. (a) FE-SEM image, elemental mapping of (b) C, (c) N, and (d) Cu, and (e) EDS spectrum of Cu@N-C(600).

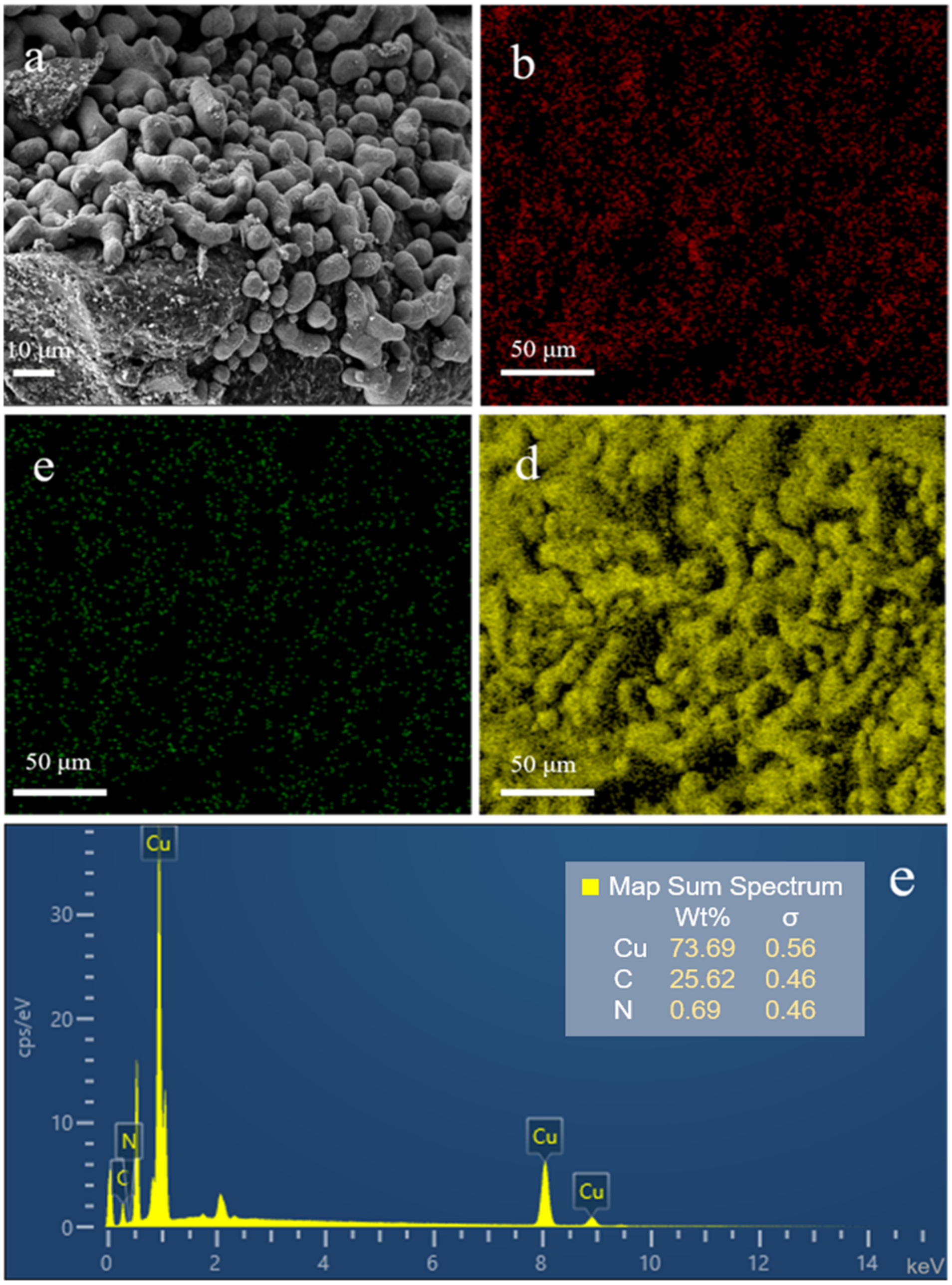

Figure S4. (a) FE-SEM image, elemental mapping of (b) C, (c) N, and (d) Cu, and (e) EDS spectrum of Cu@N-C(800).

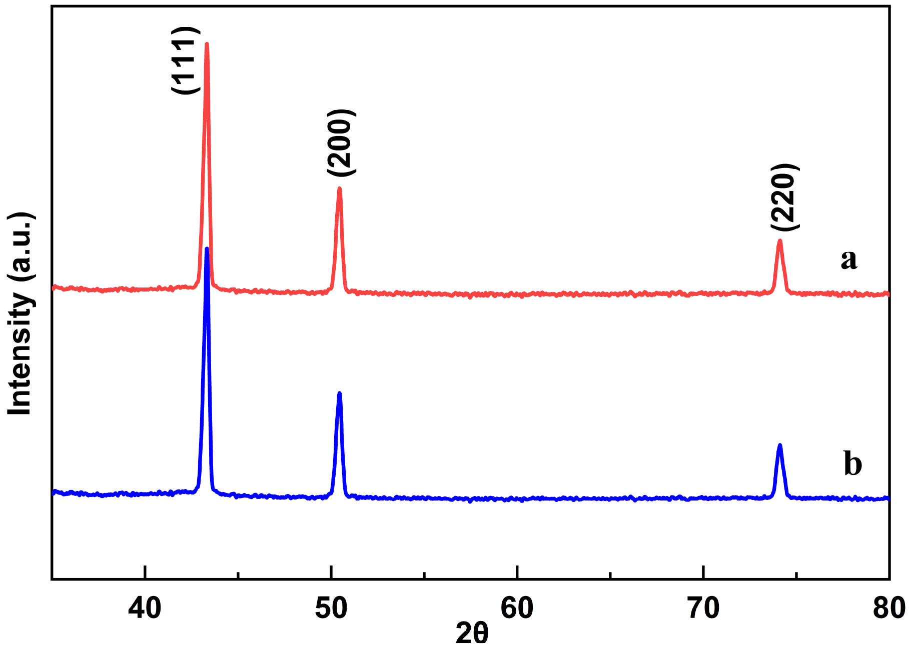

Figure S5. Powder XRD pattern of (**a**) fresh and (**b**) used Cu@N-C(600).

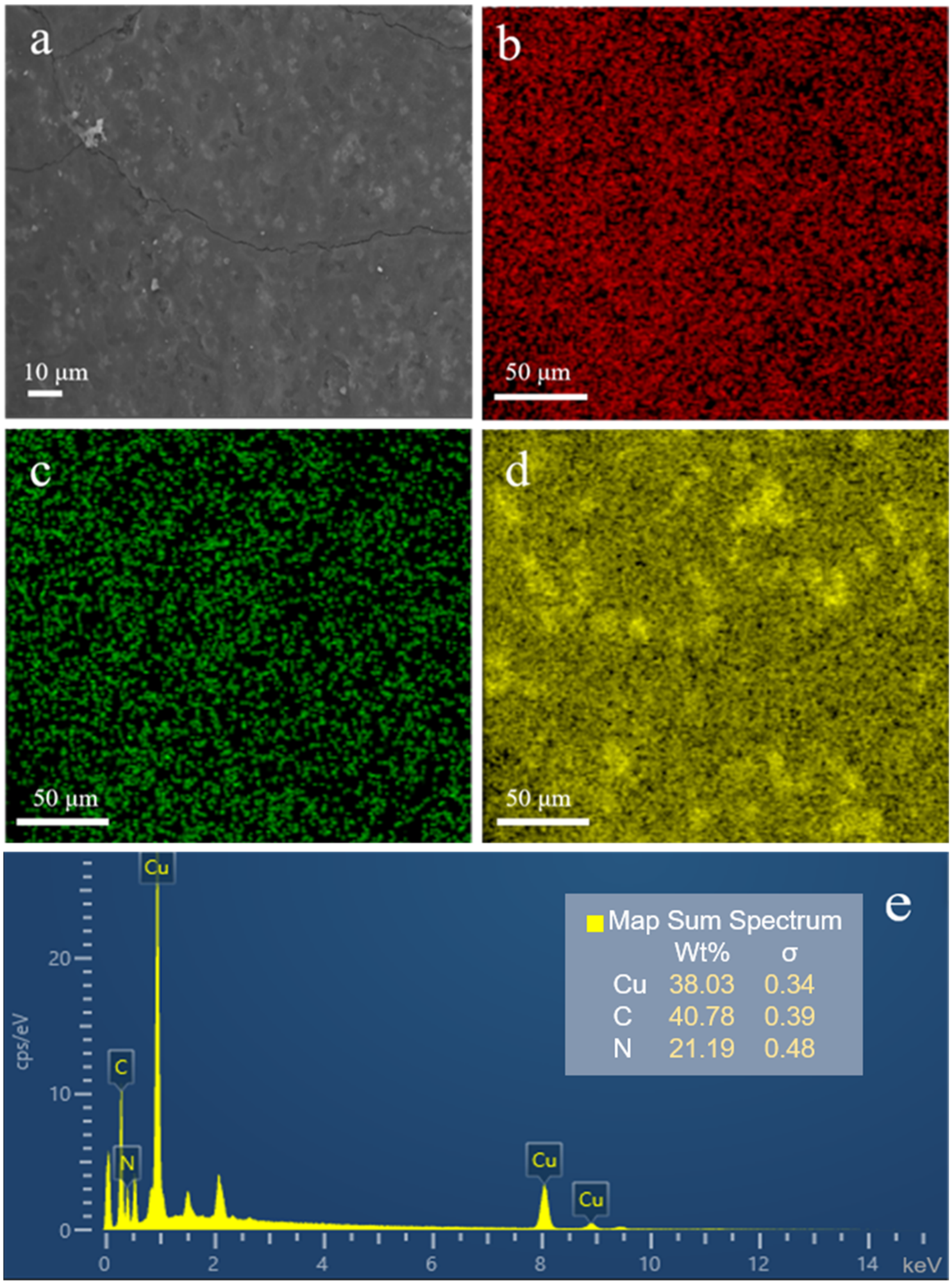

Figure S6. (a) FE-SEM image, elemental mapping of (b) C, (c) N, and (d) Cu, and (e) EDS spectrum of the used Cu@N-C(600) (the first run).

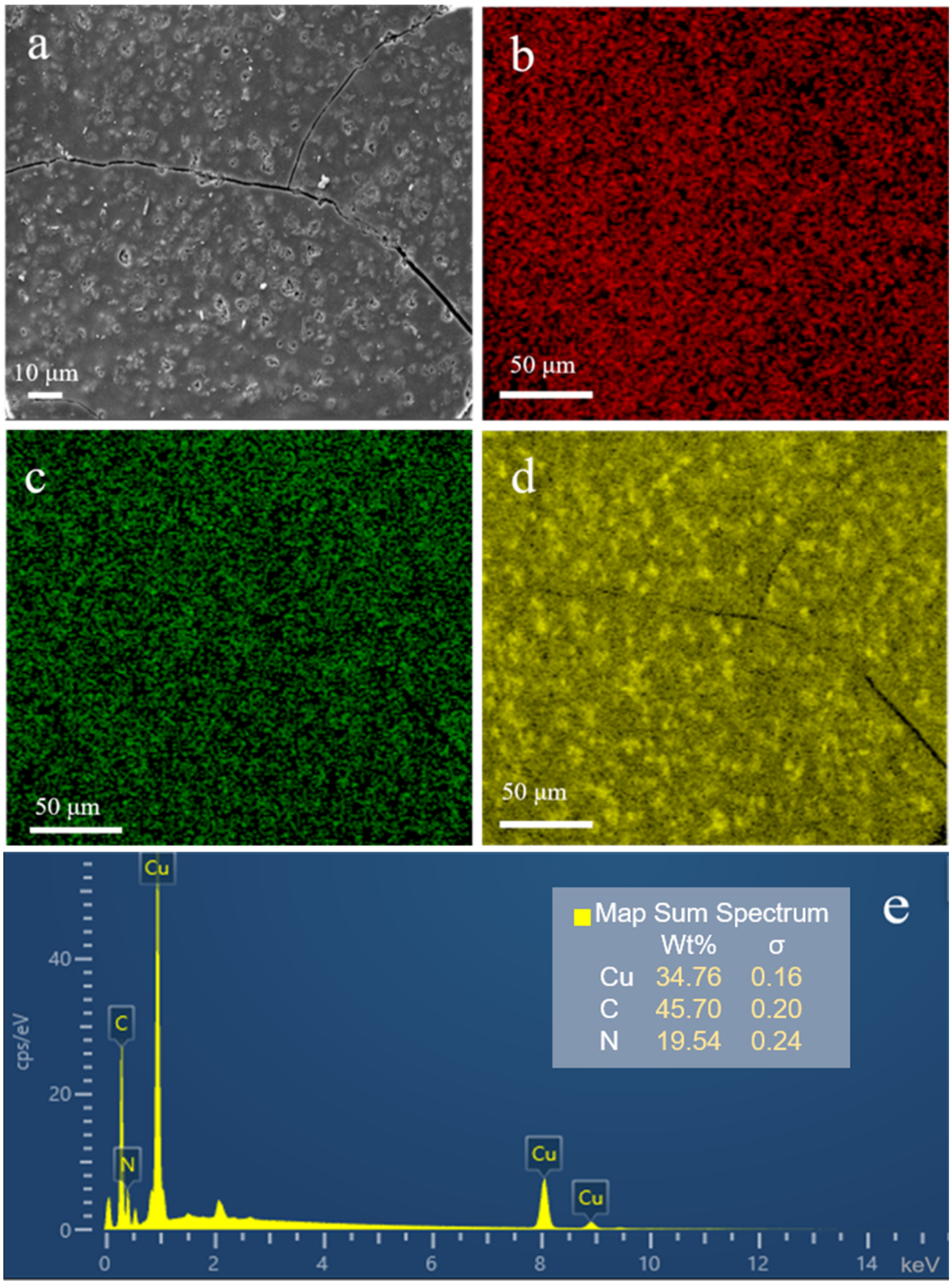

Figure S7. (a) FE-SEM image, elemental mapping of (b) C, (c) N, and (d) Cu, and (e) EDS spectrum of the used Cu@N-C(600) (the second run).

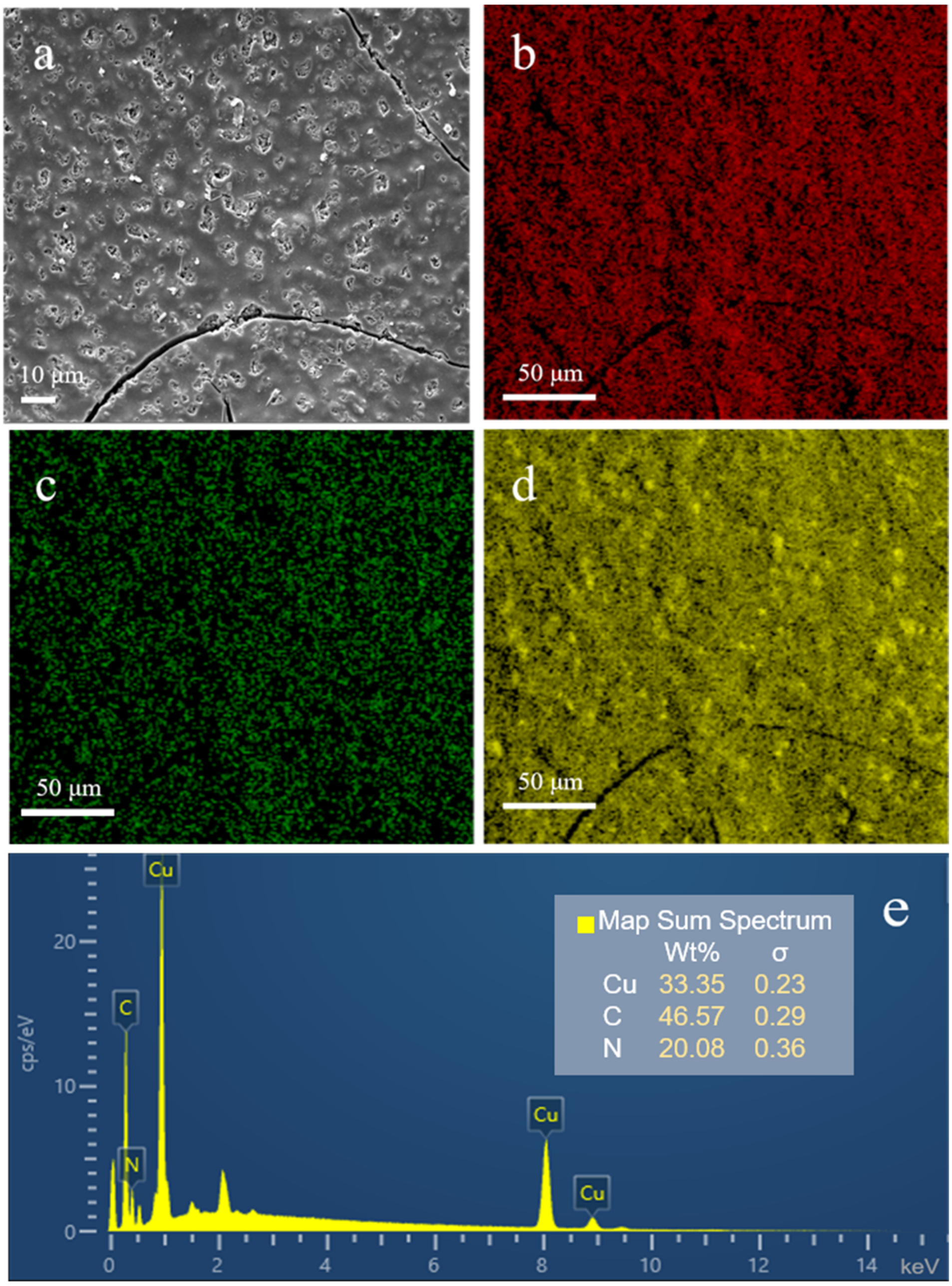

Figure S8. (a) FE-SEM image, elemental mapping of (b) C, (c) N, and (d) Cu, and (e) EDS spectrum of the used Cu@N-C(600) (the fourth run).

Table S1. Comparison of catalytic performance between Cu@N-C(600) and other types of catalysts.

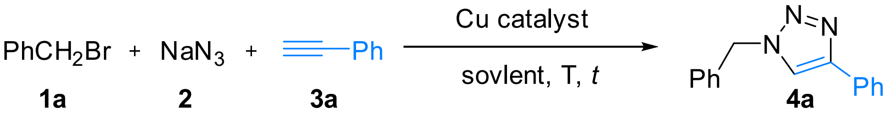

Entry	Catalyst	Yield	Reference
1	Cu(BTC)-MOF, CH_3OH, rt, 16 h	91%	[23]
2	$CuFe_2O_4$@Starch, H_2O, 30 °C, 24 h	92%	[48]

3	$CuAl_2O_4$ NPs, H_2O, 90 °C, 45 min	97%	[49]
4	Cu-PEI@Fe_3O_4 MNPs, H_2O/PEG, 70 °C, 3 h	96%	[50]
5	[P4-VP]$CuSO_4$/NaAsc, t-BuOH/H_2O (1:1), 70 °C, 40 min	91%	[51]
6	Cu@N-C(600), t-BuOH/H_2O (3:1), 50 °C, 12 h	98%	This work

Characterization data of compound 4a-5b

Benzyl-4-phenyl-1H-1,2,3-triazole (4a) [43]

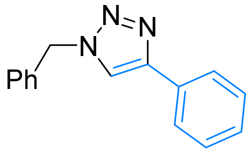

98% yield for benzyl bromide, and 90% for benzyl chloride. White solid.

^{1}H NMR (400 MHz, $CDCl_3$): δ 7.84-7.76 (m, 2H), 7.67 (s, 1H), 7.44-7.35 (m, 5H), 7.34-7.29 (m, 3H), 5.57 (s, 2H).

^{13}C NMR (100 MHz, $CDCl_3$): δ 148.20, 134.66, 130.46, 129.18, 128.83, 128.82, 128.22, 128.09, 125.71, 119.55, 54.27.

Benzyl-4-(p-tolyl)-1H-1,2,3-triazole (4b) [23]

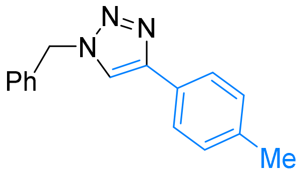

90% yield. White solid.

^{1}H NMR (400 MHz, $CDCl_3$): δ 7.69 (d, *J* = 8.1 Hz, 2H), 7.63 (s, 1H), 7.40-7.35 (m, 3H), 7.34-7.27 (m, 2H), 7.20 (d, *J* = 7.9 Hz, 2H), 5.56 (s, 2H), 2.36 (s, 3H).

^{13}C NMR (100 MHz, $CDCl_3$): δ 148.26, 138.07, 134.71, 129.51, 129.16, 128.78, 128.08, 127.62, 125.62, 119.20, 54.25, 21.32.

1-Benzyl-4-(4-ethylphenyl)-1H-1,2,3-triazole (4c) [43]

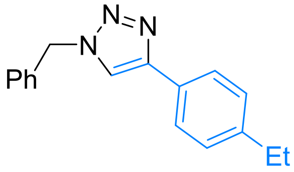

93% yield. White solid.

^{1}H NMR (400 MHz, $CDCl_3$): δ 7.72 (d, *J* = 8.2 Hz, 2H), 7.63 (s, 1H), 7.42-7.35 (m, 3H), 7.34-7.26 (m, 2H), 7.23 (d, *J* = 8.1 Hz, 2H), 5.57 (s, 2H), 2.66 (q, *J* = 7.6 Hz, 2H), 1.25 (t, *J* = 7.6 Hz, 3H).

^{13}C NMR (100 MHz, $CDCl_3$): δ 148.27, 144.47, 134.72, 129.16, 128.78, 128.33, 128.06, 127.84, 125.71, 119.24, 54.25, 28.69, 15.56.

Benzyl-4-(4-methoxyphenyl)-1H-1,2,3-triazole (4d) [43]

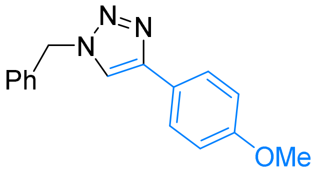

90% yield. White solid.

^{1}H NMR (400 MHz, $CDCl_3$): δ 7.72 (d, *J* = 8.7 Hz, 2H), 7.58 (s, 1H), 7.43-7.33 (m, 3H), 7.35-7.26 (m, 2H), 6.93 (d, *J* = 8.7 Hz, 2H), 5.56 (s, 2H), 3.82 (s, 3H).

^{13}C NMR (100 MHz, $CDCl_3$): δ 159.60, 148.06, 134.75, 129.15, 128.77, 128.07, 127.02, 123.20, 118.74, 114.21, 55.33, 54.23.

Benzyl-4-(4-methoxyphenyl)-1H-1,2,3-triazole (4e) [43]

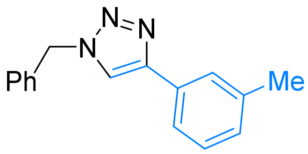

92% yield. White solid.

^{1}H NMR (400 MHz, $CDCl_3$): δ 7.65 (s, 2H), 7.59-7.54 (m, 1H), 7.42-7.35 (m, 3H), 7.32-7.25 (m, 3H), 7.16-7.10 (m, 1H), 5.56 (s, 2H), 2.37 (s, 3H).

^{13}C NMR (100 MHz, $CDCl_3$): δ 148.31, 138.52, 134.70, 130.33, 129.17, 128.98, 128.80, 128.73, 128.08, 126.39, 122.81, 119.53, 54.25, 21.44.

Benzyl-4-(o-tolyl)-1*H*-1,2,3-triazole (4f) [52]

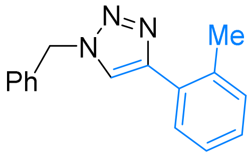

90% yield. White solid.

^{1}H NMR (400 MHz, $CDCl_3$): δ 7.75 (dd, *J* = 5.3, 2.4 Hz, 1H), 7.57 (s, 1H), 7.38 (d, *J* = 7.5 Hz, 3H), 7.31 (d, *J* = 7.8 Hz, 2H), 7.25 (d, *J* = 2.9 Hz, 3H), 5.60 (s, 2H), 2.43 (s, 3H).

^{13}C NMR (100 MHz, $CDCl_3$): δ 147.52, 135.49, 134.78, 130.87, 129.80, 129.17, 128.89, 128.77, 128.19, 127.99, 126.09, 121.71, 54.19, 21.42.

Benzyl-4-(4-fluorophenyl)-1*H*-1,2,3-triazole (4g) [43]

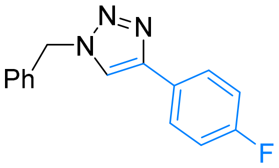

97% yield. White solid.

^{1}H NMR (400 MHz, $CDCl_3$): δ 7.80-7.74 (m, 2H), 7.63 (s, 1H), 7.43-7.36 (m, 3H), 7.34-7.29 (m, 2H), 7.09 (t, *J* = 8.7 Hz, 2H), 5.57 (s, 2H).

^{13}C NMR (100 MHz, $CDCl_3$): δ 162.68 (d, *J* = 245.8 Hz), 147.31, 134.52, 129.22, 128.89, 128.12, 127.47 (d, *J* = 8.2 Hz), 126.62 (d, *J* = 3.2 Hz), 119.31, 115.83 (d, *J* = 21.6), 54.35.

^{19}F NMR (376 MHz, $CDCl_3$): δ -113.48.

Benzyl-4-(4-chlorophenyl)-1*H*-1,2,3-triazole (4h) [43]

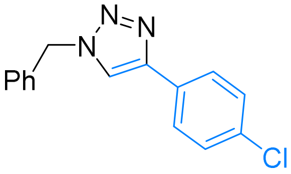

91% yield. White solid.

^{1}H NMR (400 MHz, $CDCl_3$): δ 7.73 (d, *J* = 8.5 Hz, 2H), 7.65 (s, 1H), 7.45-7.34 (m, 5H), 7.35-7.28 (m, 2H), 5.57 (s, 2H).

^{13}C NMR (100 MHz, $CDCl_3$): δ 147.17, 134.49, 133.91, 129.23, 129.02, 128.91, 128.83, 128.14, 126.95, 119.57, 54.34.

Benzyl-4-(4-bromophenyl)-1*H*-1,2,3-triazole (4i) [43]

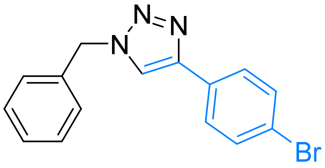

90% yield. White solid.

^{1}H NMR (400 MHz, $CDCl_3$): δ 7.68 (s, 1H), 7.54-7.50 (m, 2H), 7.56-7.48 (m, 2H), 7.45-7.33 (m, 3H), 7.36-7.27 (m, 2H), 5.57 (s, 2H).

^{13}C NMR (100 MHz, $CDCl_3$): δ 147.13, 134.43, 131.97, 129.37, 129.24, 128.93, 128.15, 127.25, 122.12, 119.62, 54.38.

Benzyl-4-(3-fluorophenyl)-1*H*-1,2,3-triazole (4j) [43]

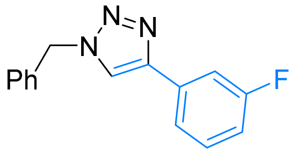

93% yield. White solid.

^{1}H NMR (400 MHz, $CDCl_3$): δ 7.68 (s, 1H), 7.59-7.48 (m, 2H), 7.43-7.33 (m, 4H), 7.35-7.28 (m, 2H), 7.06-6.95 (m, 1H), 5.58 (s, 2H).

^{13}C NMR (100 MHz, $CDCl_3$): δ 163.13 (d, J = 244.3 Hz), 147.13 (d, J = 2.9 Hz), 134.47, 132.65 (d, J = 8.5 Hz), 130.41 (d, J = 8.4 Hz), 129.23, 128.91, 128.13, 121.31 (d, J = 2.9 Hz), 119.92, 115.00 (d, J = 21.2 Hz), 112.64 (d, J = 23.1 Hz), 54.34.

^{19}F NMR (376 MHz, $CDCl_3$): δ -112.69.

Benzyl-4-(3-bromophenyl)-1*H*-1,2,3-triazole (4k) [53]

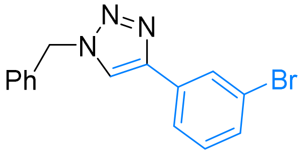

91% yield. White solid.

^{1}H NMR (400 MHz, $CDCl_3$): δ 7.94 (s, 1H), 7.74 (d, J = 7.8 Hz, 1H), 7.67 (s, 1H), 7.47-7.35 (m, 4H), 7.33-7.24 (m, 3H), 5.58 (s, 2H).

^{13}C NMR (100 MHz, $CDCl_3$): δ 146.79, 134.41, 132.49, 131.10, 130.40, 129.25, 128.95, 128.65, 128.15, 124.24, 122.91, 119.89, 54.39.

2-(1-Benzyl-1*H*-1,2,3-triazol-4-yl)propan-2-ol (4l) [43]

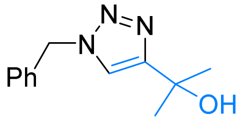

97% yield. White solid.

^{1}H NMR (400 MHz, $CDCl_3$): δ 7.41-7.33 (m, 3H), 7.33 (s, 1H), 7.31-7.24 (m, 2H), 5.49 (s, 2H), 1.61 (s, 6H).

^{13}C NMR (100 MHz, $CDCl_3$): δ 156.02, 134.57, 129.13, 128.77, 128.18, 119.07, 68.52, 54.17, 30.43.

Benzyl-4-butyl-1*H*-1,2,3-triazole (4m) [43]

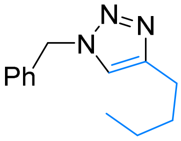

66% yield. White solid.

^1H NMR (400 MHz, $CDCl_3$): δ 7.40-7.33 (m, 3H), 7.27-7.24 (m, 2H), 7.19 (s, 1H), 5.49 (s, 2H), 2.69 (t, *J* = 7.5 Hz, 2H), 1.66-1.59 (m, 2H), 1.39-1.33 (m, 2H), 0.91 (t, *J* = 7.4 Hz, 3H).

^{13}C NMR (100 MHz, $CDCl_3$): δ 148.94, 134.99, 129.06, 128.61, 127.97, 120.51, 54.00, 31.53, 25.41, 22.34, 13.84.

1-(4-Methylbenzyl)-4-phenyl-1*H*-1,2,3-triazole (4n) [43]

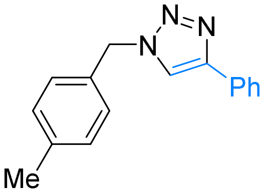

86% yield. White solid.

^1H NMR (400 MHz, $CDCl_3$): δ 7.82-7.75 (m, 2H), 7.64 (s, 1H), 7.39 (m, 2H), 7.35-7.26 (m, 1H), 7.23-7.17 (m, 4H), 5.52 (s, 2H), 2.36 (s, 3H).

^{13}C NMR (100 MHz, $CDCl_3$): δ 148.13, 138.77, 131.62, 130.54, 129.83, 128.81, 128.16, 126.93, 125.69, 119.43, 54.08, 21.22.

1-(4-Methylbenzyl)-4-phenyl-1*H*-1,2,3-triazole (4o) [54]

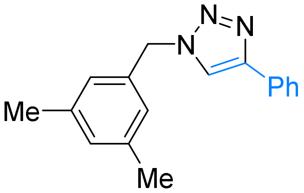

88% yield. White solid.

^1H NMR (400 MHz, $CDCl_3$): δ 7.83-7.78 (m, 2H), 7.66 (s, 1H), 7.40 (t, *J* = 7.5 Hz, 2H), 7.35-7.28 (m, 1H), 6.99 (s, 1H), 6.92 (s, 2H), 5.48 (s, 2H), 2.30 (s, 6H).

^{13}C NMR (100 MHz, $CDCl_3$): δ 148.10, 138.89, 134.48, 130.54, 130.41, 128.82, 128.17, 125.90, 125.70, 119.55, 54.28, 21.25.

1-(3-Methoxybenzyl)-4-phenyl-1*H*-1,2,3-triazole (4p) [55]

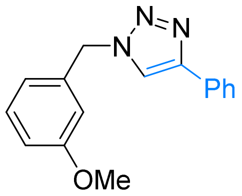

88% yield. White solid.

^{1}H NMR (400 MHz, $CDCl_3$): δ 7.83-7.77 (m, 2H), 7.68 (s, 1H), 7.43-7.37 (m, 2H), 7.36-7.27 (m, 2H), 6.97-6.86 (m, 2H), 6.83 (s, 1H), 5.54 (s, 2H), 3.78 (s, 3H).

^{13}C NMR (100 MHz, $CDCl_3$): δ 160.13, 136.09, 130.47, 130.26, 128.91, 128.83, 128.21, 125.71, 120.28, 119.57, 114.27, 113.65, 55.34, 54.23.

1-(3,5-Dimethoxybenzyl)-4-phenyl-1*H*-1,2,3-triazole (4q) [56]

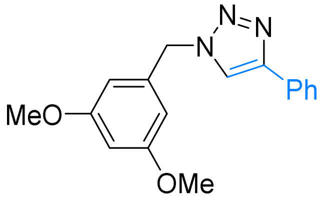

82% yield. White solid.

^{1}H NMR (400 MHz, $CDCl_3$): δ 7.80 (d, J = 7.1 Hz, 2H), 7.69 (s, 1H), 7.40 (t, J = 7.5 Hz, 2H), 7.32 (t, J = 7.4 Hz, 1H), 6.44 (s, 3H), 5.49 (s, 2H), 3.76 (s, 6H).

^{13}C NMR (100 MHz, $CDCl_3$): δ 161.34, 148.19, 136.76, 130.46, 128.83, 128.21, 125.71, 119.56, 106.08, 100.44, 55.46, 54.34.

1-(4-(Tert-butyl)benzyl)-4-phenyl-1*H*-1,2,3-triazole (4r) [57]

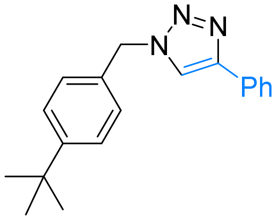

87% yield. White solid.

^{1}H NMR (400 MHz, $CDCl_3$): δ 7.82-7.78 (m, 2H), 7.67 (s, 1H), 7.42-7.37 (m, 4H), 7.33-7.28 (m, 1H), 7.25 (d, J = 6.4 Hz, 2H), 5.54 (s, 2H), 1.31 (s, 9H).

^{13}C NMR (100 MHz, $CDCl_3$): δ 151.95, 148.12, 131.63, 130.54, 128.81, 128.16, 127.92, 126.10, 125.71, 119.53, 53.99, 34.68, 31.29.

1-(4-Fluorobenzyl)-4-phenyl-1*H*-1,2,3-triazole (4s) [43]

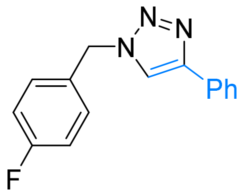

80% yield for 4-fluorobenzyl bromide and 94% yield for 4-fluorobenzyl chloride. White solid.

^{1}H NMR (400 MHz, $CDCl_3$): δ 7.85-7.74 (m, 2H), 7.67 (s, 1H), 7.40 (t, *J* = 7.5 Hz, 2H), 7.36-7.26 (m, 3H), 7.08 (t, *J* = 8.6 Hz, 2H), 5.55 (s, 2H).

^{13}C NMR (100 MHz, $CDCl_3$): δ 162.88 (d, *J* = 246.6 Hz), 148.33, 130.53 (d, *J* = 3.3 Hz), 130.36, 129.96 (d, *J* = 8.4 Hz), 128.86, 128.29, 125.71, 119.41, 116.19 (d, *J* = 21.8 Hz), 53.51.

^{19}F NMR (376 MHz, $CDCl_3$): δ -112.60.

1-(4-Chlorobenzyl)-4-phenyl-1*H*-1,2,3-triazole (4t) [43]

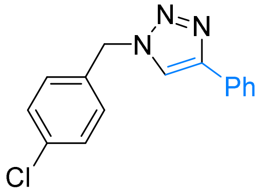

72% yield. White solid.

^{1}H NMR (400 MHz, $CDCl_3$): δ 8.01-7.74 (m, 2H), 7.67 (s, 1H), 7.61-7.27 (m, 5H), 7.26-7.23 (m, 2H), 5.54 (s, 2H).

^{13}C NMR (100 MHz, $CDCl_3$): δ 148.37, 134.86, 133.16, 130.29, 129.39, 128.87, 128.33, 125.72, 119.47, 100.66, 53.52.

1-(4-Bromobenzyl)-4-phenyl-1*H*-1,2,3-triazole (4u) [43]

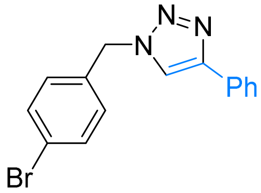

70% yield. White solid.

^{1}H NMR (400 MHz, $CDCl_3$): δ 7.84-7.77 (m, 2H), 7.68 (s, 1H), 7.59-7.46 (m, 2H), 7.41 (t, *J* = 7.4 Hz, 2H), 7.33 (t, *J* = 7.4 Hz, 1H), 7.18 (d, *J* = 8.3 Hz, 2H), 5.53 (s, 2H).

^{13}C NMR (100 MHz, $CDCl_3$): δ 133.66, 132.35, 131.96, 130.26, 129.68, 128.88, 128.35, 125.73, 122.99, 119.51, 53.59.

1-(4-Nitrobenzyl)-4-phenyl-1*H*-1,2,3-triazole (4v) [48]

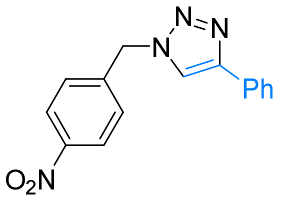

85% yield. White solid.

^{1}H NMR (400 MHz, $CDCl_3$): δ 8.23 (d, *J* = 8.7 Hz, 2H), 7.85-7.77 (m, 2H), 7.77 (s, 1H), 7.52-7.38 (m, 4H), 7.37-7.30 (m, 1H), 5.70 (s, 2H).

^{13}C NMR (100 MHz, $CDCl_3$): δ 148.69, 148.06, 141.77, 130.05, 128.94, 128.57, 128.53, 125.75, 124.35, 119.78, 53.20.

4-Phenyl-1-(4-(trifluoromethyl)benzyl)-1*H*-1,2,3-triazole (4w) [58]

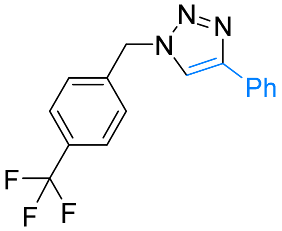

90% yield. White solid.

^{1}H NMR (400 MHz, $CDCl_3$): δ 7.84-7.79 (m, 2H), 7.71 (s, 1H), 7.65 (d, *J* = 8.1 Hz, 2H), 7.44-7.39 (m, 4H), 7.38-7.29 (m, 1H), 5.65 (s, 2H).

^{13}C NMR (100 MHz, $CDCl_3$): δ 148.56, 138.64, 131.07 (d, *J* = 32.8 Hz), 130.20, 128.89, 128.41, 128.19, 126.17 (q, *J* = 3.8 Hz), 125.74, 125.12, 122.41, 119.61, 53.58.

^{19}F NMR (376 MHz, $CDCl_3$): δ -62.75.

1-(2-Fluorobenzyl)-4-phenyl-1*H*-1,2,3-triazole (4x) [48]

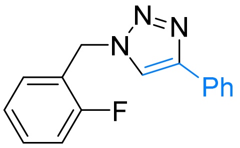

94% yield. White solid.

^{1}H NMR (400 MHz, $CDCl_3$): δ 7.94-7.79 (m, 2H), 7.77 (s, 1H), 7.52-7.27 (m, 5H), 7.20-7.07 (m, 2H), 5.64 (s, 2H).

^{13}C NMR (100 MHz, $CDCl_3$): δ 160.53 (d, *J* = 246.2 Hz), 148.23, 130.96 (d, *J* = 8.2 Hz), 130.61 (d, *J* = 20.1 Hz), 128.84, 128.24, 125.73, 124.91 (d, *J* = 3.7 Hz), 121.97 (d, *J* = 14.6 Hz), 119.71, 115.86 (d, *J* = 21.2 Hz), 47.78.

^{19}F NMR (376 MHz, $CDCl_3$): δ -118.15.

1-(2-Chlorobenzyl)-4-phenyl-1*H*-1,2,3-triazole (4y) [59]

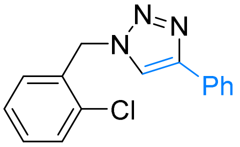

95% yield. White solid.

^{1}H NMR (400 MHz, $CDCl_3$): δ 7.84-7.80 (m, 2H), 7.78 (s, 1H), 7.47-7.37 (m, 3H), 7.35-7.26 (m, 3H), 7.26-7.19 (m, 1H), 5.71 (s, 2H).

^{13}C NMR (100 MHz, $CDCl_3$): δ 148.13, 133.44, 132.52, 130.40, 130.28, 130.14, 129.95, 128.85, 128.26, 127.67, 125.74, 119.85, 51.50.

1-(2,5-Difluorobenzyl)-4-phenyl-1*H*-1,2,3-triazole (4z) [60]

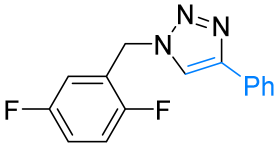

82% yield. White solid.

^{1}H NMR (400 MHz, $CDCl_3$): δ 7.95-7.68 (m, 3H), 7.47-7.36 (m, 2H), 7.38-7.27 (m, 1H), 7.20-6.93 (m, 3H), 5.61 (s, 2H).

^{13}C NMR (100 MHz, $CDCl_3$): δ 159.97 (d, J = 2.5 Hz), 157.55 (t, J = 2.2 Hz), 155.14 (d, J = 2.7 Hz), 148.41, 130.23, 128.88, 128.37, 125.76, 123.51 (dd, J = 17.3, 7.8 Hz), 119.76, 117.50 (d, J = 8.6 Hz), 117.22 (dd, J = 8.4, 7.6 Hz), 116.98, 116.92 (d, J = 4.7 Hz), 116.72 (d, J = 3.6 Hz), 47.43.

^{19}F NMR (376 MHz, $CDCl_3$): δ -116.98 (d, J = 18.0 Hz), -124.10 (d, J = 17.8 Hz).

1-(3-Fluorobenzyl)-4-phenyl-1*H*-1,2,3-triazole (4aa) [49]

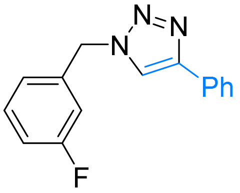

90% yield. White solid.

^{1}H NMR (400 MHz, $CDCl_3$): δ 7.84-7.78 (m, 2H), 7.72 (s, 1H), 7.46-7.29 (m, 4H), 7.18-6.90 (m, 3H), 5.57 (s, 2H).

^{13}C NMR (101 MHz, $CDCl_3$): δ 163.02 (d, J = 246.4 Hz), 148.35, 137.02 (d, J = 7.3 Hz), 130.85 (d, J = 8.2 Hz), 130.24, 128.88, 128.36, 125.75, 123.55 (d, J = 3.0 Hz), 119.63, 115.85 (d, J = 21.0 Hz), 115.02 (d, J = 22.2 Hz), 53.63 .

^{19}F NMR (376 MHz, $CDCl_3$): δ -111.49.

1-(3-Chlorobenzyl)-4-phenyl-1*H*-1,2,3-triazole (4ab) [59]

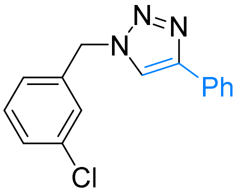

94% yield. White solid.

^{1}H NMR (400 MHz, $CDCl_3$): δ 7.91-7.76 (m, 2H), 7.71 (s, 1H), 7.41 (t, J = 7.5 Hz, 2H), 7.36-7.29 (m, 4H), 7.21-7.12 (m, 1H), 5.55 (s, 2H).

^{13}C NMR (100 MHz, $CDCl_3$): δ 148.41, 136.61, 135.05, 130.49, 130.28, 129.03, 128.88, 128.35, 128.09, 126.09, 125.74, 119.58, 53.55.

1-(3-Bromobenzyl)-4-phenyl-1*H*-1,2,3-triazole (4ac) [48]

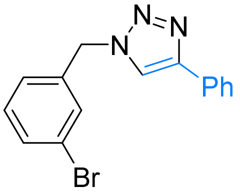

90% yield. White solid.

^{1}H NMR (400 MHz, $CDCl_3$): δ 7.85-7.77 (m, 2H), 7.71 (s, 1H), 7.52-7.39 (m, 4H), 7.36-7.30 (m, 1H), 7.26-7.17 (m, 2H), 5.54 (s, 2H).

^{13}C NMR (100 MHz, $CDCl_3$): δ 148.42, 136.85, 131.98, 130.99, 130.75, 130.27, 128.88, 128.35, 126.57, 125.75, 123.14, 119.57, 53.48.

1-(3,4-Dichlorobenzyl)-4-phenyl-1*H*-1,2,3-triazole (4ad) [50]

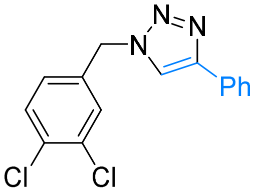

76% yield. White solid.

^{1}H NMR (400 MHz, $CDCl_3$): δ 7.85-7.77 (m, 2H), 7.72 (s, 1H), 7.60-7.38 (m, 4H), 7.36-7.31 (m, 1H), 7.17-7.10 (m, 1H), 5.53 (s, 2H).

^{13}C NMR (100 MHz, $CDCl_3$): δ 148.54, 134.79, 133.36, 133.19, 131.18, 130.15, 129.89, 128.91, 128.43, 127.20, 125.75, 119.55, 52.96.

1-(3-Chloro-4-fluorobenzyl)-4-phenyl-1*H*-1,2,3-triazole (4ae) [50]

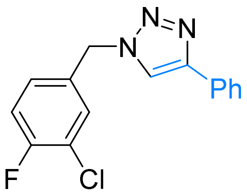

94% yield. White solid.

^{1}H NMR (400 MHz, $CDCl_3$): δ 7.87-7.77 (m, 2H), 7.71 (s, 1H), 7.46-7.31 (m, 4H), 7.24-7.07 (m, 2H), 5.54 (s, 2H).

^{13}C NMR (100 MHz, $CDCl_3$): δ 158.26 (d, *J* = 249.4 Hz), 148.51, 131.78 (d, *J* = 4.1 Hz), 130.34, 130.18, 128.90, 128.41, 127.87 (d, *J* = 7.6 Hz), 125.74, 121.89 (d, *J* = 18.1 Hz), 119.47, 117.36 (d, *J* = 21.5 Hz), 52.99.

^{19}F NMR (376 MHz, $CDCl_3$): δ -114.70.

(8R, 9S, 10R, 13S, 14S, 17S)-17-(1-benzyl-1*H*-1, 2, 3-triazol-4-yl)-17-hydroxy-10, 13-dimethyl-1, 2, 6, 7, 8, 9, 10, 11, 12, 13, 14, 15, 16, 17-tetra decahy dro-3*H*-cyclopenta[a] phenan thren-3-one (5a)

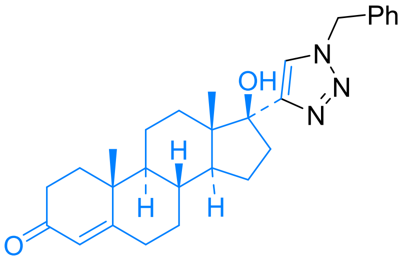

84% yield. White solid.

^{1}H NMR (400 MHz, DMSO-d_6): δ 7.84 (s, 1H), 7.39-7.27 (m, 5H), 5.60 (s, 1H), 5.55 (s, 2H), 5.10 (s, 1H), 2.41-2.19 (m, 4H), 2.10 (d, *J* = 16.8 Hz, 1H), 2.02-1.66 (m, 4H), 1.62-1.26 (m, 7H), 1.13 (s, 3H), 0.93-0.84 (m, 4H), 0.64-0.54 (m, 1H), 0.45-0.32 (m, 1H).

^{13}C NMR (100 MHz, DMSO-d_6): δ 198.44, 171.43, 154.86, 136.77, 129.16, 128.48, 128.31, 123.54, 123.21, 81.40, 53.55, 53.06, 48.56, 46.74, 38.63, 37.64, 36.09, 35.47, 34.07, 32.85, 32.51, 32.03, 24.31, 20.69, 17.40, 14.83, 14.83.

HRMS (APCI) m/z calcd for $C_{28}H_{36}O_2N_3^+$ $(M+H)^+$ 446.28020, found 446.27948.

1-((2R,4S,5S)-5-(hydroxymethyl)-4-(4-phenyl-1*H*-1,2,3-triazol-1-yl)tetrahydrofuran-2-yl)-5-methylpyrimidine-2,4(1*H*,3*H*)-dione (5b)

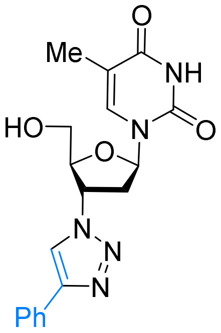

100% yield. White solid.

^{1}H NMR (400 MHz, DMSO-d_6): δ 11.37 (s, 1H), 8.79 (s, 1H), 7.87 (s, 1H), 7.85 (s, 2H), 7.47 (t, *J* = 7.6 Hz, 2H), 7.35 (t, *J* = 7.4 Hz, 1H), 6.46 (t, *J* = 6.6 Hz, 1H), 5.42-5.39 (m, 1H), 5.31 (t, *J* = 5.2 Hz, 1H), 4.33-4.28 (m, 1H), 3.77-3.68 (m, 2H), 2.85-2.78 (m, 1H), 2.75-2.67 (m, 1H), 1.83 (s, 3H).

^{13}C NMR (100 MHz, DMSO-d_6): δ 164.20, 150.91, 147.03, 136.72, 131.04, 129.41, 128.46, 125.63, 121.47, 110.11, 84.90, 84.37, 61.23, 59.84, 37.63, 12.74.

HRMS (APCI) m/z calcd for $C_{18}H_{20}O_4N_5^+$ $(M+H)^+$370.15098, found 370.15039.

References

[48] Bonyasi, R.; Gholinejad, M.; Saadati, F.; Nájera, C. Copper ferrite nanoparticle modified starch as a highly recoverable catalyst for room temperature click chemistry: multicomponent synthesis of 1,2,3-triazoles in water. *New J. Chem.* **2018,** *42*, 3078-3086.

[49] Khalili, D.; Kavoosi, L.; Khalafi-Nezhad, A. Copper aluminate spinel in click chemistry: an efficient heterogeneous nanocatalyst for the highly regioselective synthesis of triazoles in water. *Synlett* **2019**, *30*, 2136-2142.

[50] Hasanpour, Z.; Maleki, A.; Hosseini, M.; Gorgannezhad, L.; Nejadshafiee, V.; Ramazani, A.; Haririan, I.; Shafiee, A.; Khoobi, M. Efficient multicomponent synthesis of 1,2,3-triazoles catalyzed by Cu(II) supported on PEI[Rate]Fe_3O_4 MNPs in a water/PEG300 system. *Turk. J. Chem.* **2017**, *41*, 294-307.

[51] Karimi Zarchi, M.A.; Nazem, F. One-pot three-component synthesis of 1,4-disubstituted 1*H*-1,2,3-triazoles using green and recyclable cross-linked poly(4-vinylpyridine)-supported copper sulfate/sodium ascorbate in water/t-BuOH system. *J. Iran. Chem. Soc.* **2014,** *11*, 1731-1742.

[52] Haito, A.; Yamaguchi, M.; Chatani, N. $Ru_3(CO)_{12}$-catalyzed carbonylation of C−H bonds by triazole-directed C−H activation. *Asian J. Org. Chem.* **2018**, *7*, 1315-1318.

[53] Zheng, Z.; Shi, L. An efficient regioselective copper-catalyzed approach to the synthesis of 1,2,3-triazoles from N-tosylhydrazones and azides. *Tetrahedron Lett.* **2016**, *57*, 5132-5134.

[54] Dehbanipour, Z.; Moghadam, M.; Tangestaninejad, S.; Mirkhani, V.; Mohammadpoor-Baltork, I. Copper(II) bisthiazole complex immobilized on silica nanoparticles: Preparation, characterization and its application as a highly efficient catalyst for click synthesis of 1,2,3-triazoles. *Polyhedron* **2017**, *138*, 21-30.

[55] Zhao, Z.; Wang, X.; Si, J.; Yue, C.; Xia, C.; Li, F. Truncated concave octahedral Cu_2O nanocrystals with {hkk} high-index facets for enhanced activity and stability in heterogeneous catalytic azide–alkyne cycloaddition. *Green Chem.* **2018**, *20*, 832-837.

[56] Pérez, J.M.; Cano, R.; Ramón, D. J. Multicomponent azide–alkyne cycloaddition catalyzed by impregnated bimetallic nickel and copper on magnetite. *RSC Adv.* **2014**, *4*, 23943-23951.

[57] Larionov, V.A.; Stashneva, A.R.; Titov, A.A.; Lisov, A.A.; Medvedev, M.G.; Smol'yakov, A.F.; Tsedilin, A.M.; Shubina, E.S.; Maleev, V.I. Mechanistic study in azide–alkyne cycloaddition (CuAAC) catalyzed by bifunctional trinuclear copper(I) pyrazolate complex: Shift in rate-determining step. *J. Catal.* **2020**, *390*, 37-45.

[58] De Angelis, S.; Franco, M.; Trimini, A.; Gonzalez, A.; Sainz, R.; Degennaro, L.; Romanazzi, G.; Carlucci, C.; Petrelli, V.; de la Esperanza, A.; Goni, A.; Ferritto, R.; Acena, J.L.; Luisi, R.; Cid, M.B. A study of graphene-based copper catalysts: copper(I) nanoplatelets for batch and continuous-flow applications. *Chem. Asian J.* **2019**, *14*, 3011-3018.

[59] Rajabi, M.; Albadi, J.; Momeni. A. Click synthesis of 1,4-disubstituted-1,2,3-triazoles catalyzed by melamine-supported CuO nanoparticles as an efficient recyclable catalyst in water. *Res. Chem. Intermediat.* **2020**, *46*, 3879-3889.

[60] Ötvös, S.B.; Georgiádes, Á.; Ádok-Sipiczki, M.; Mészáros, R.; Pálinkó, I.; Sipos, P.; Fülöp, F., A layered double hydroxide. a synthetically useful heterogeneous catalyst for azide−alkyne cycloadditions in a continuous-flow reactor. *Appl. Catal. A: Gen.* **2015**, *501*, 63-73.

Copies of ^{1}H NMR, ^{13}C NMR and ^{19}F NMR spectra

1-Benzyl-4-phenyl-1*H*-1,2,3-triazole (4a)

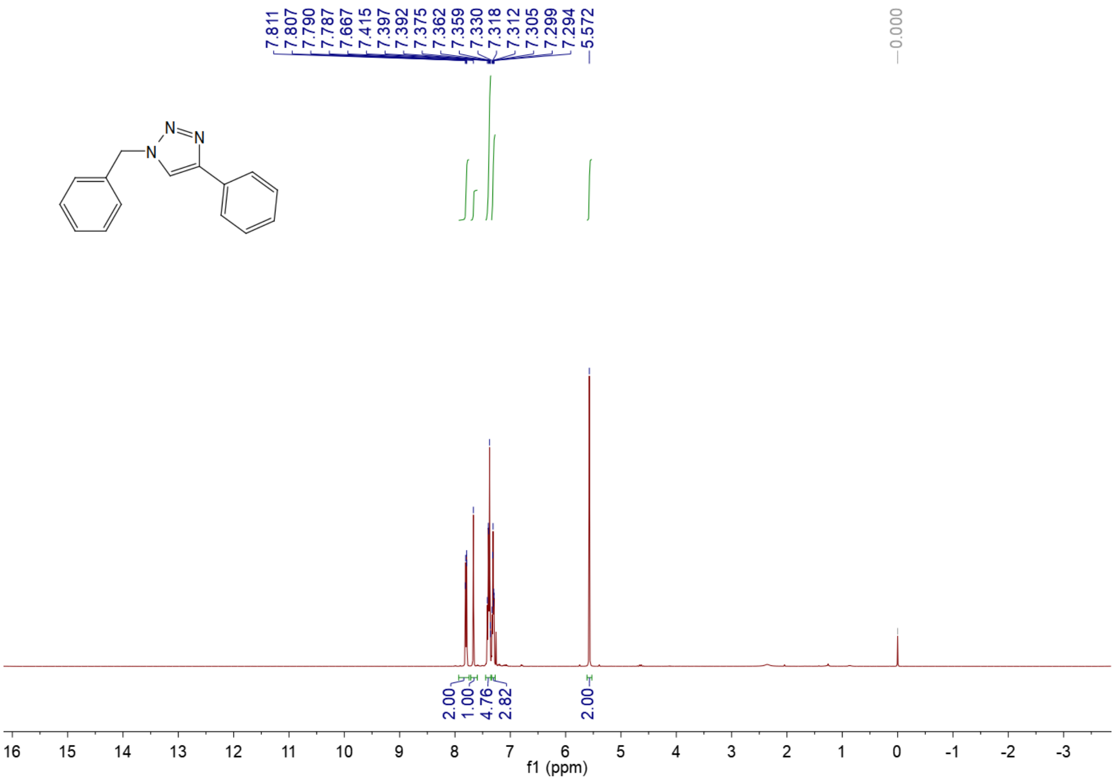

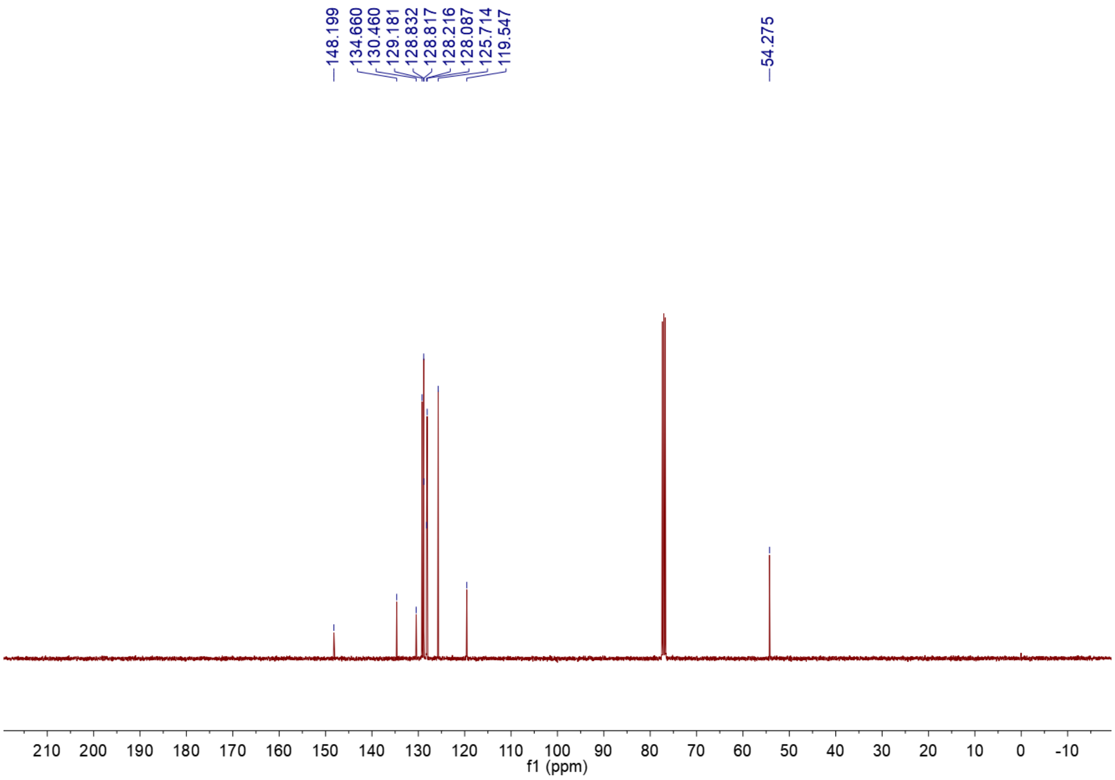

1-Benzyl-4-(p-tolyl)-1*H*-1,2,3-triazole (4b)

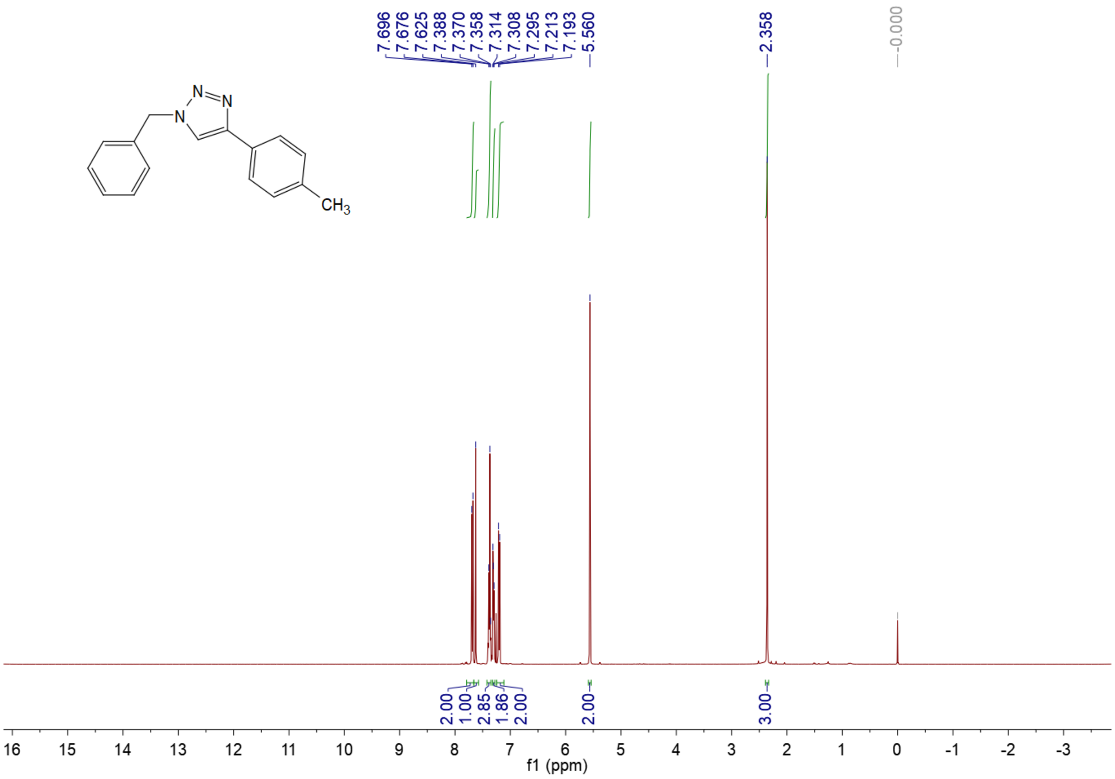

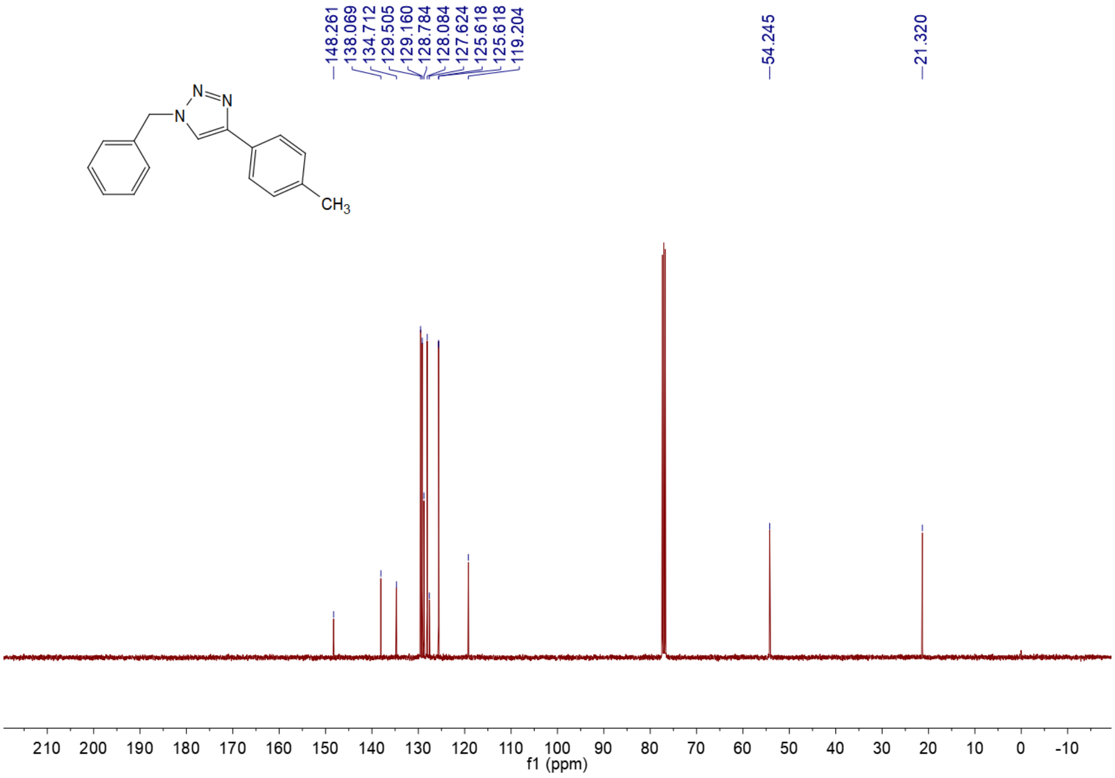

Benzyl-4-(4-ethylphenyl)-1*H*-1,2,3-triazole (4c)

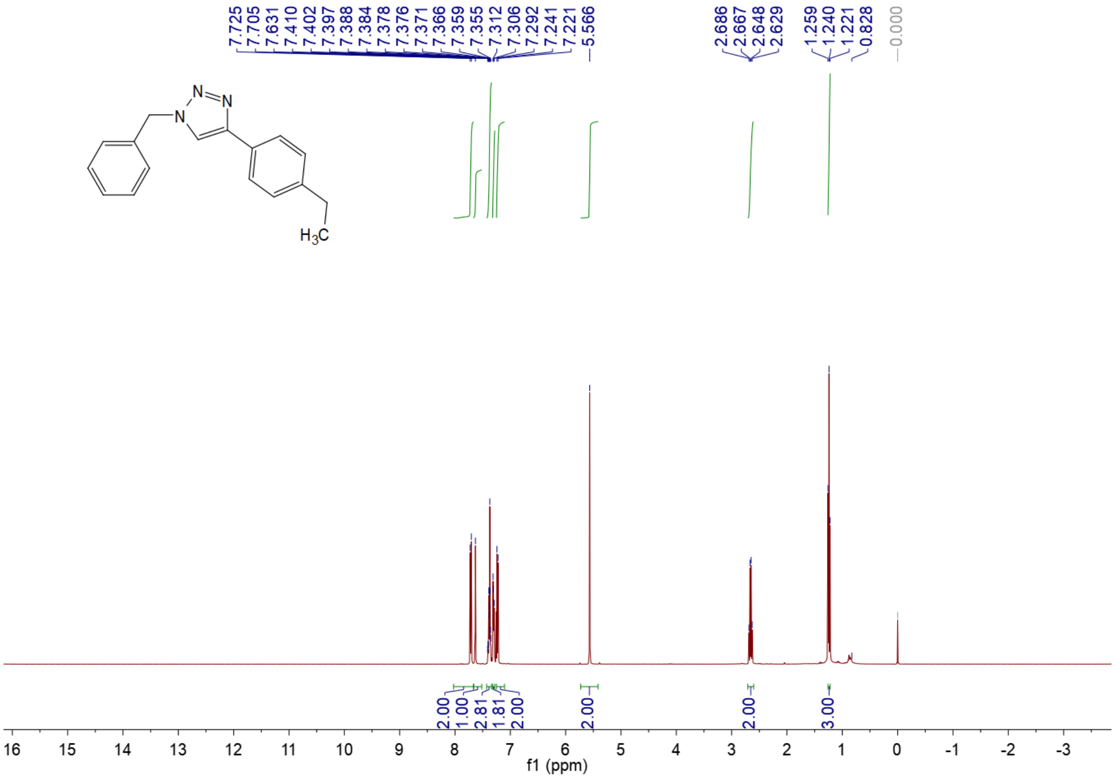

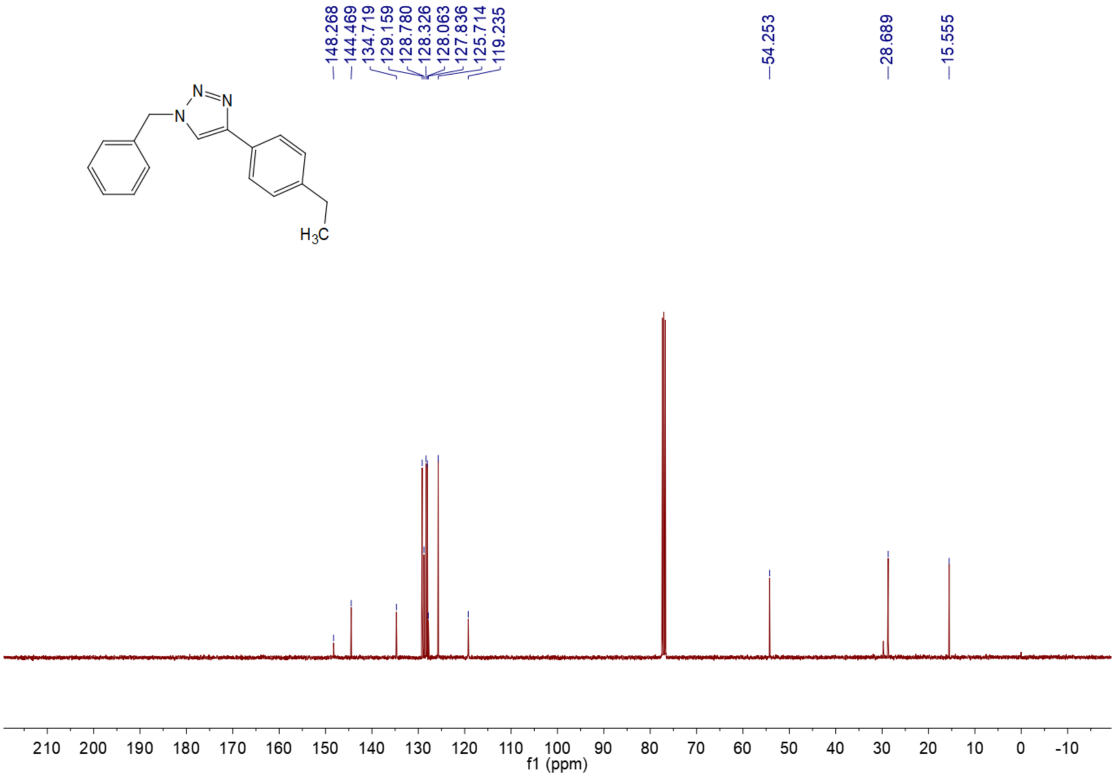

Benzyl-4-(4-methoxyphenyl)-1*H*-1,2,3-triazole (4d)

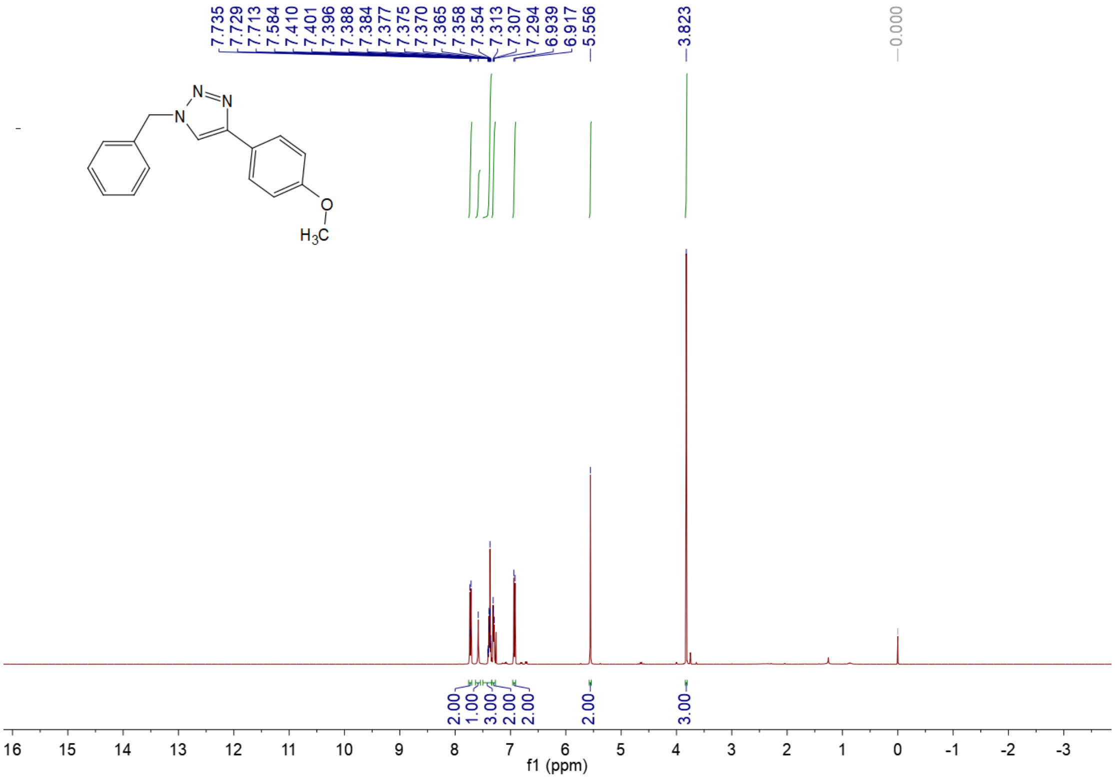

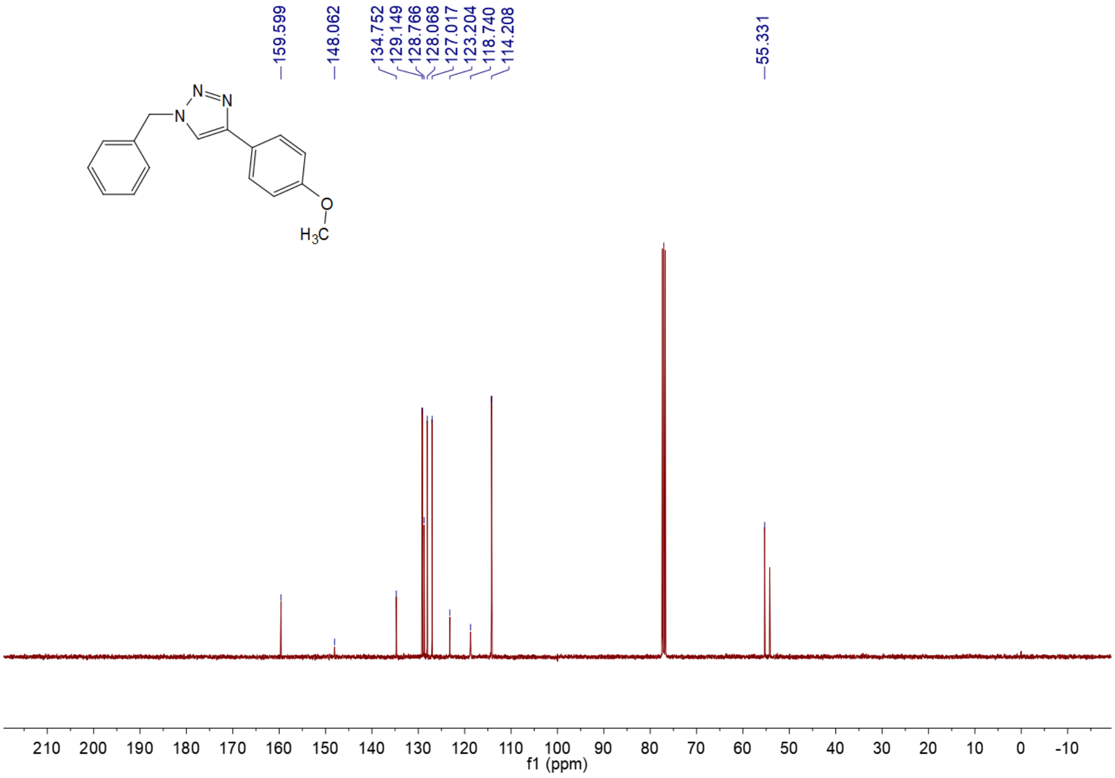

1-Benzyl-4-(4-methoxyphenyl)-1*H*-1,2,3-triazole (4e)

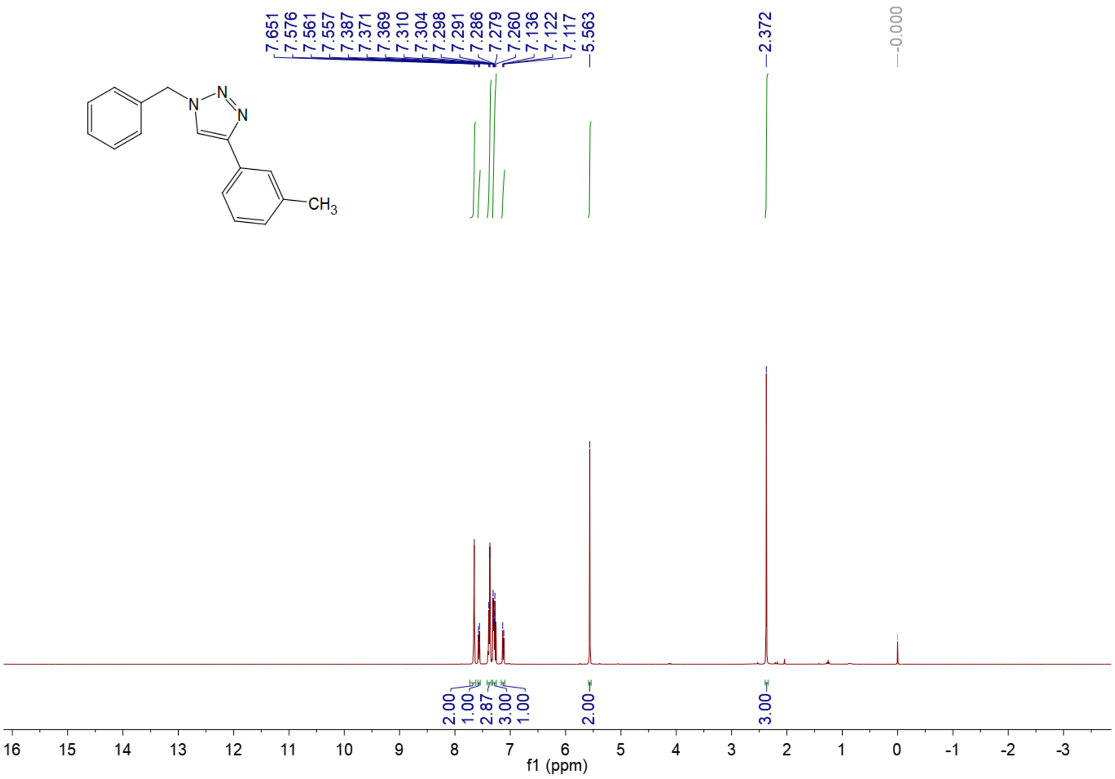

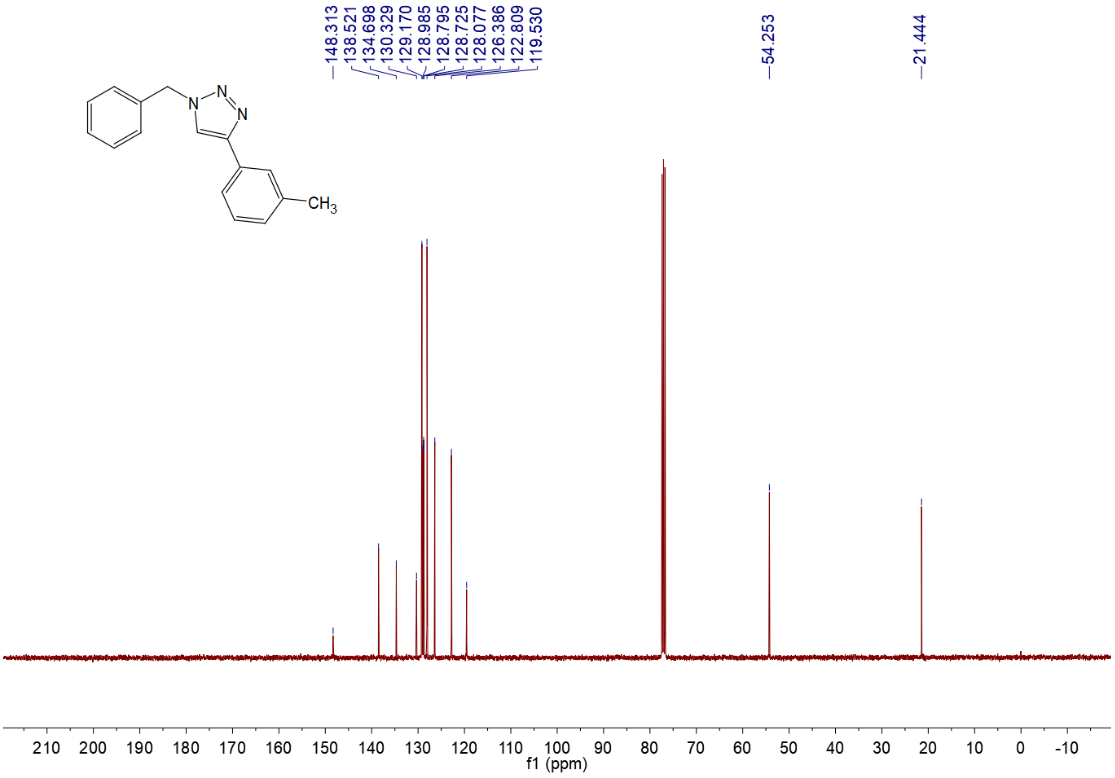

1-Benzyl-4-(o-tolyl)-1*H*-1,2,3-triazole (4f)

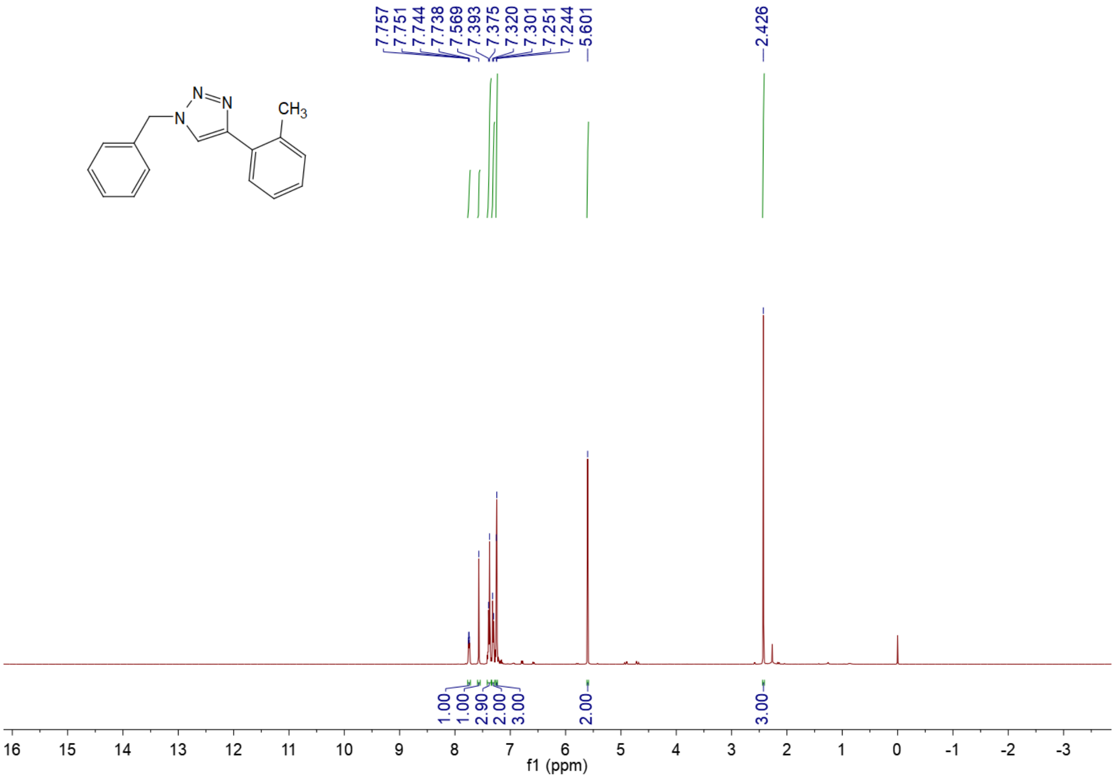

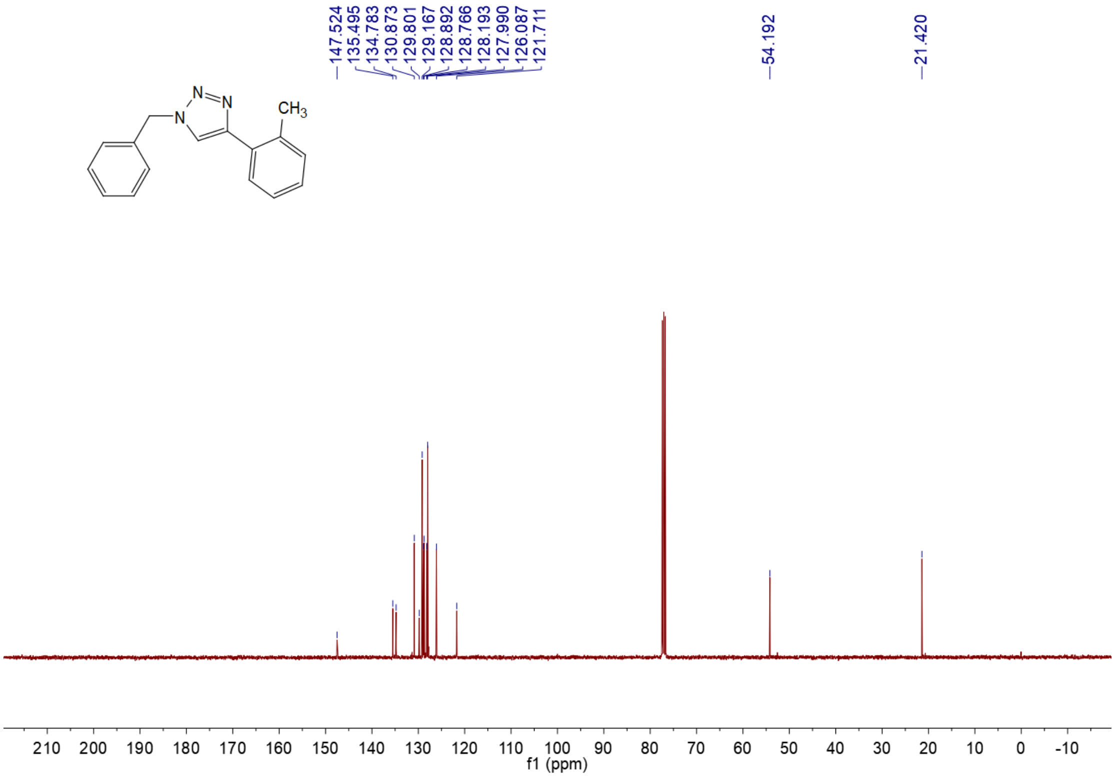

1-Benzyl-4-(4-fluorophenyl)-1*H*-1,2,3-triazole (4g)

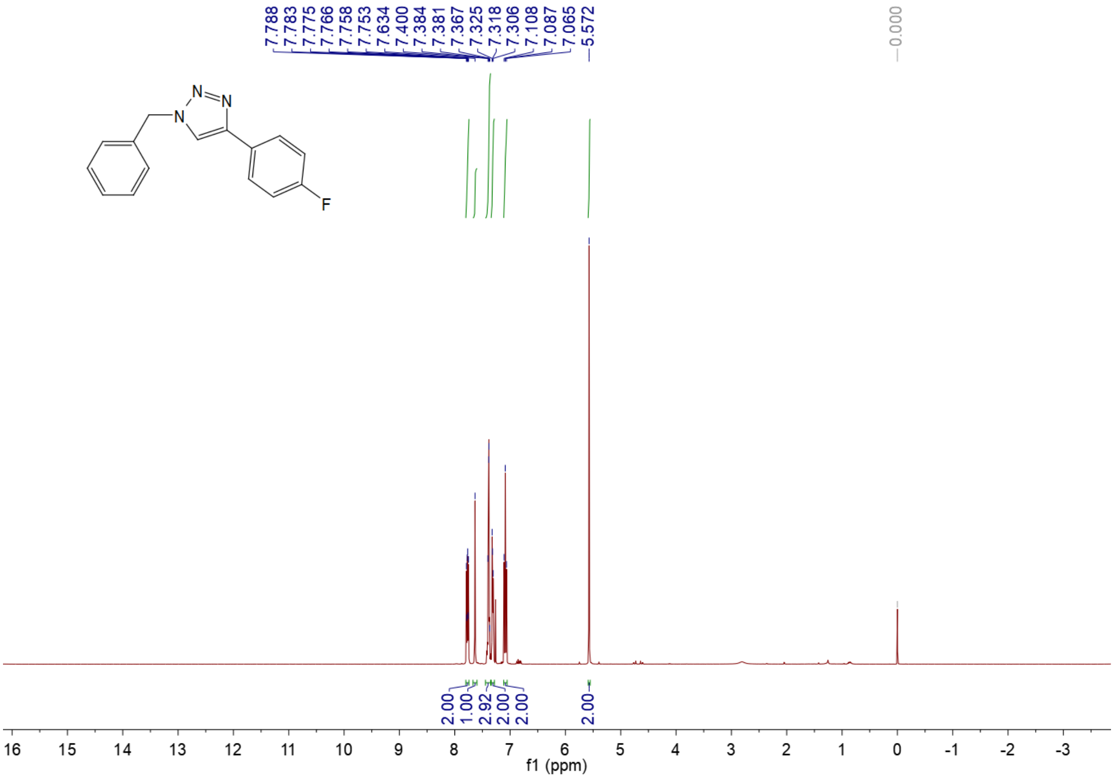

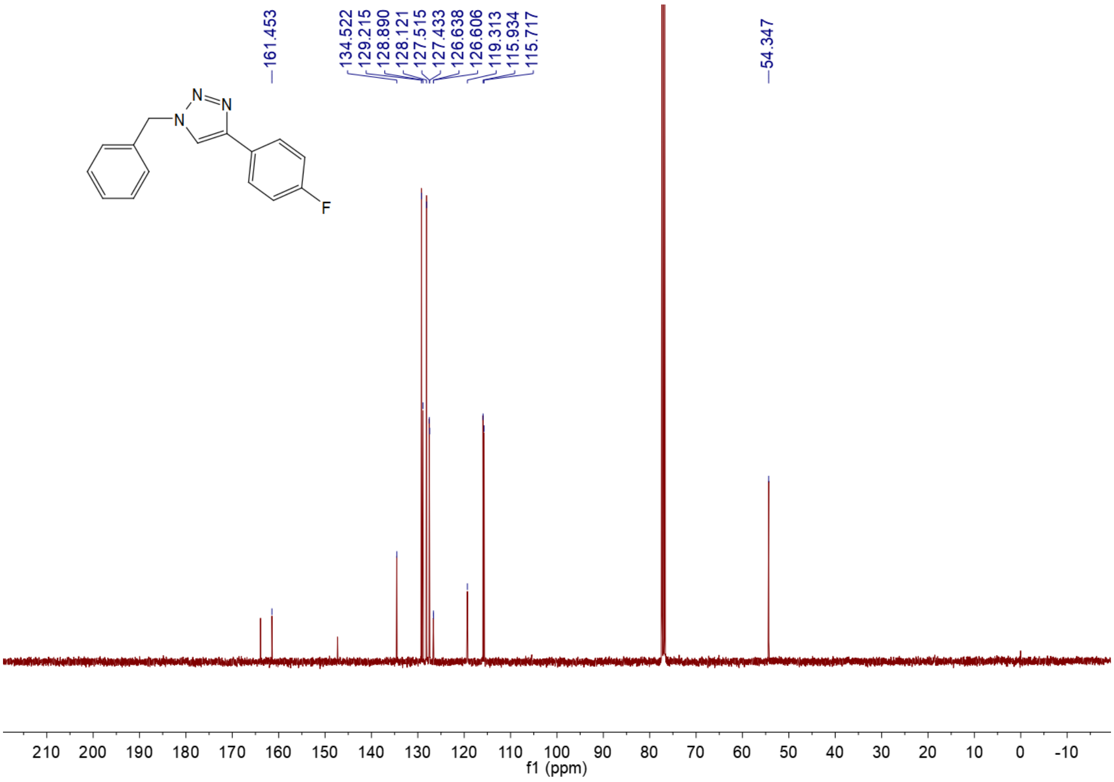

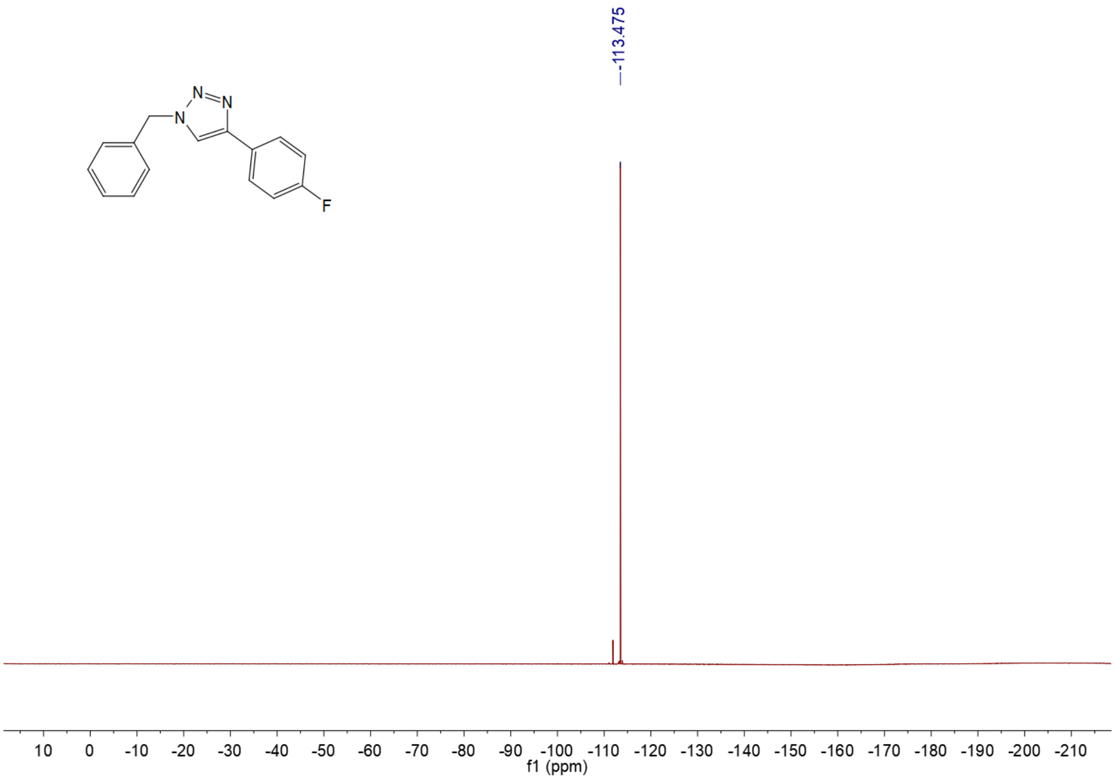
-113.475
N
N
N
F
10 0 -10 -20 -30 -40 -50 -60 -70 -80 -90 -100 -110 -120 -130 -140 -150 -160 -170 -180 -190 -200 -210
f1 (ppm)

Benzyl-4-(4-chlorophenyl)-1*H*-1,2,3-triazole (4h)

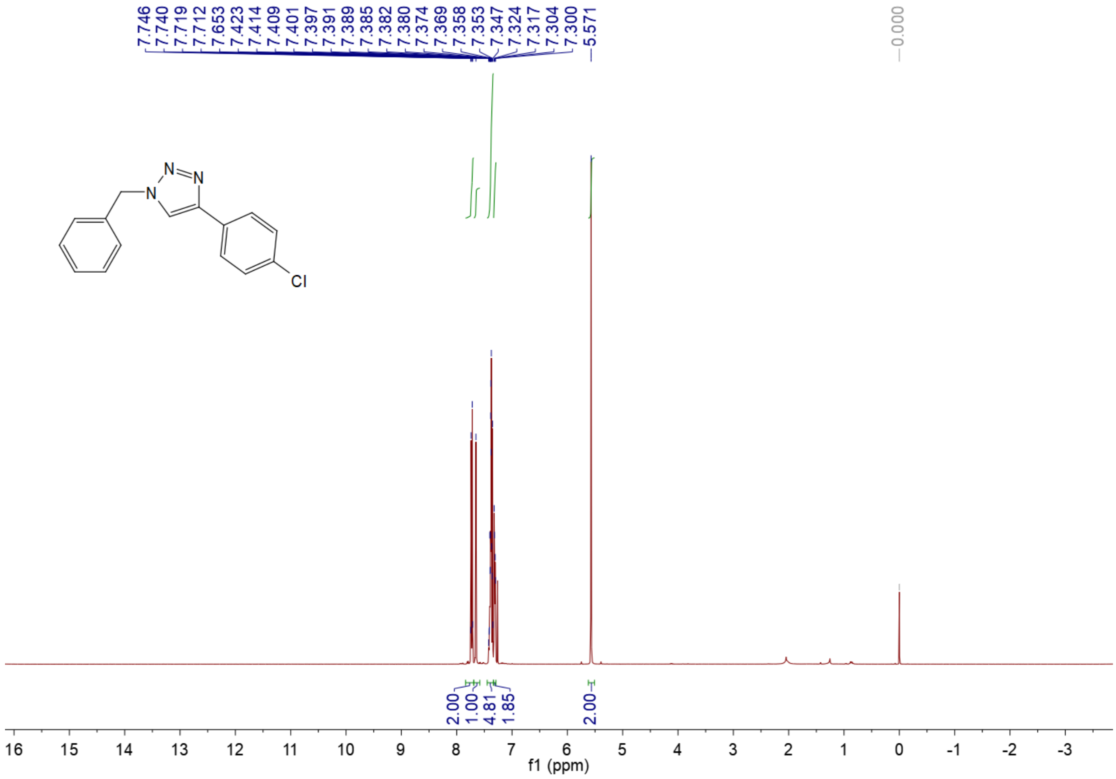

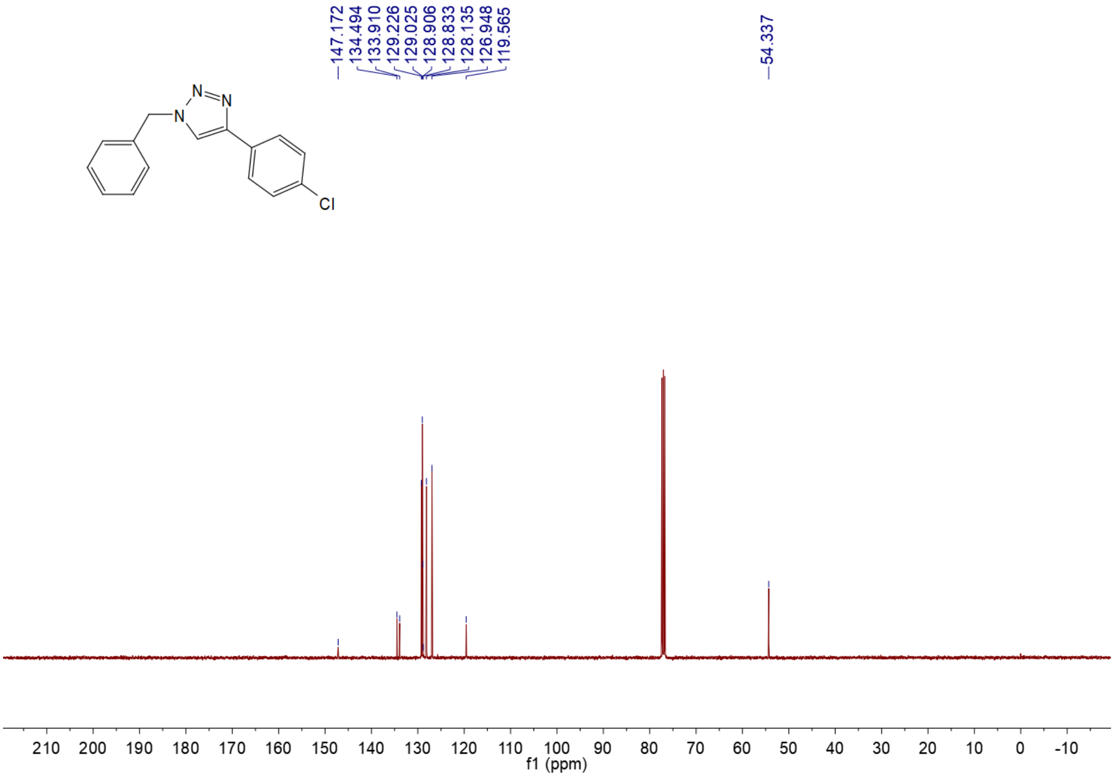

1-Benzyl-4-(4-bromophenyl)-1*H*-1,2,3-triazole (4i)

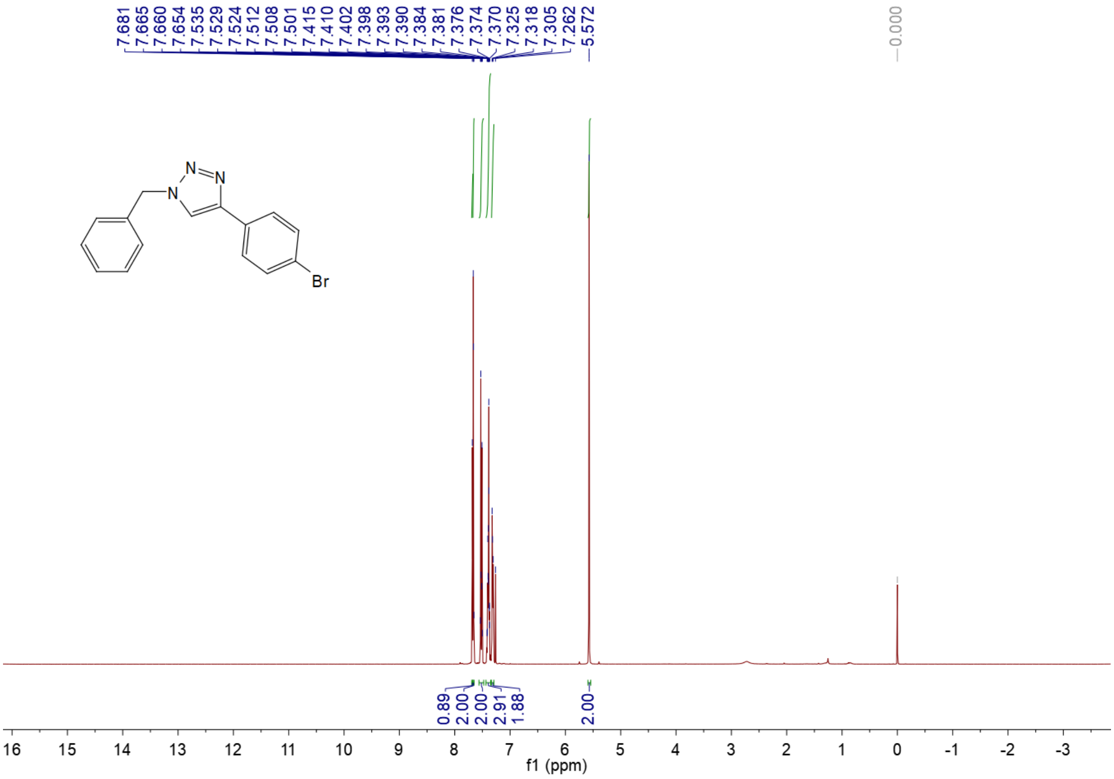

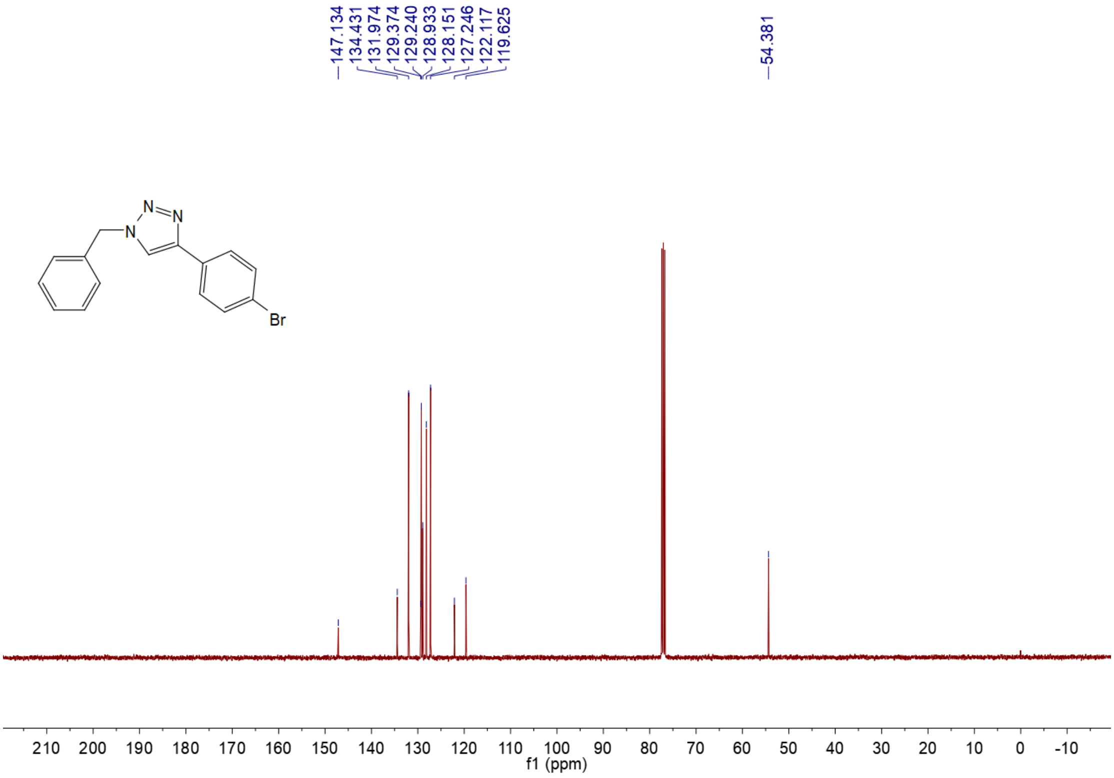

Benzyl-4-(3-fluorophenyl)-1*H*-1,2,3-triazole (4j)

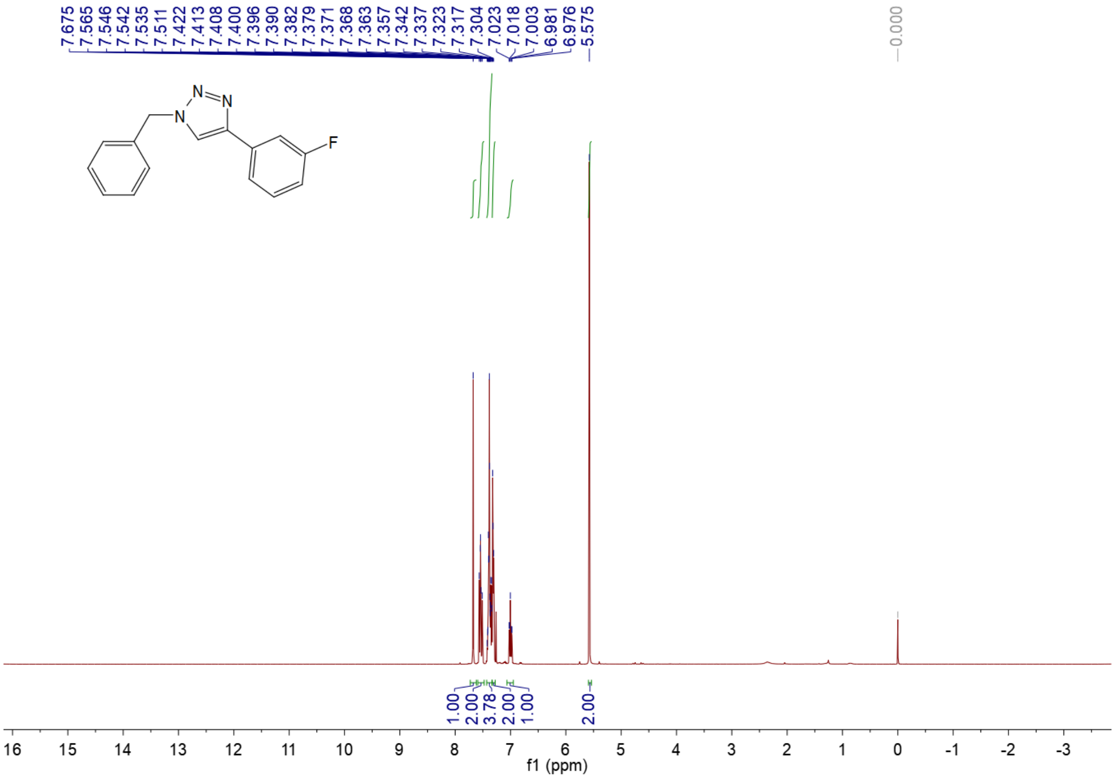

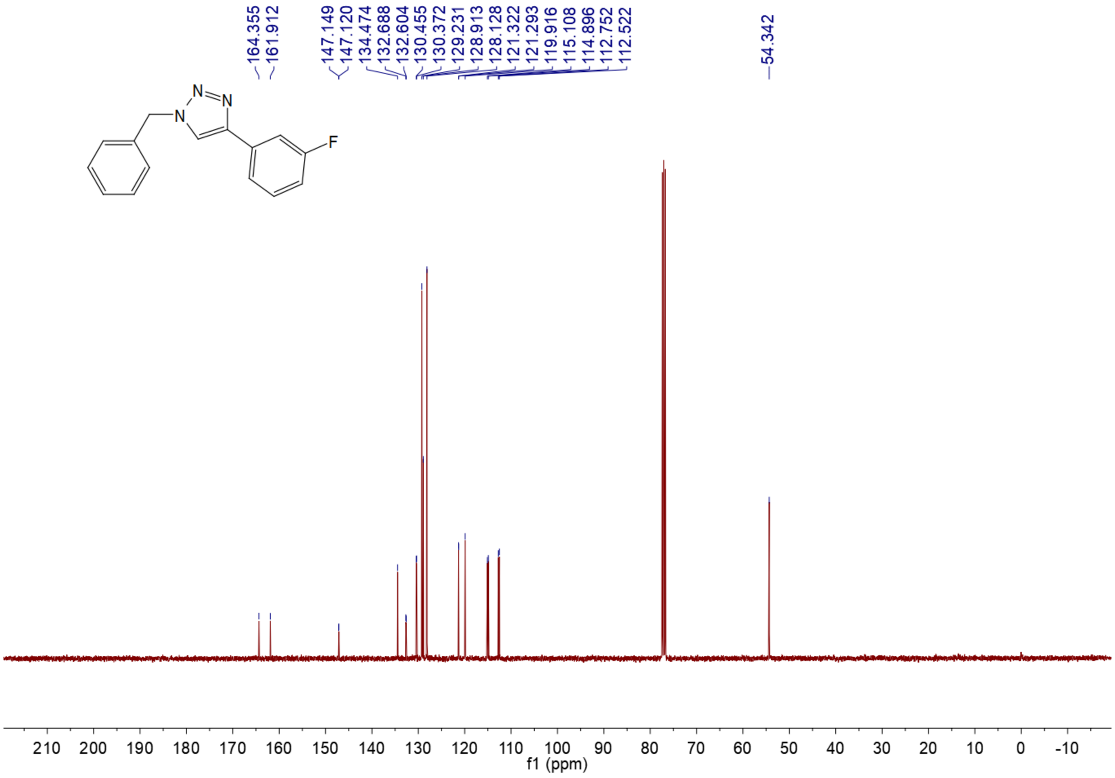

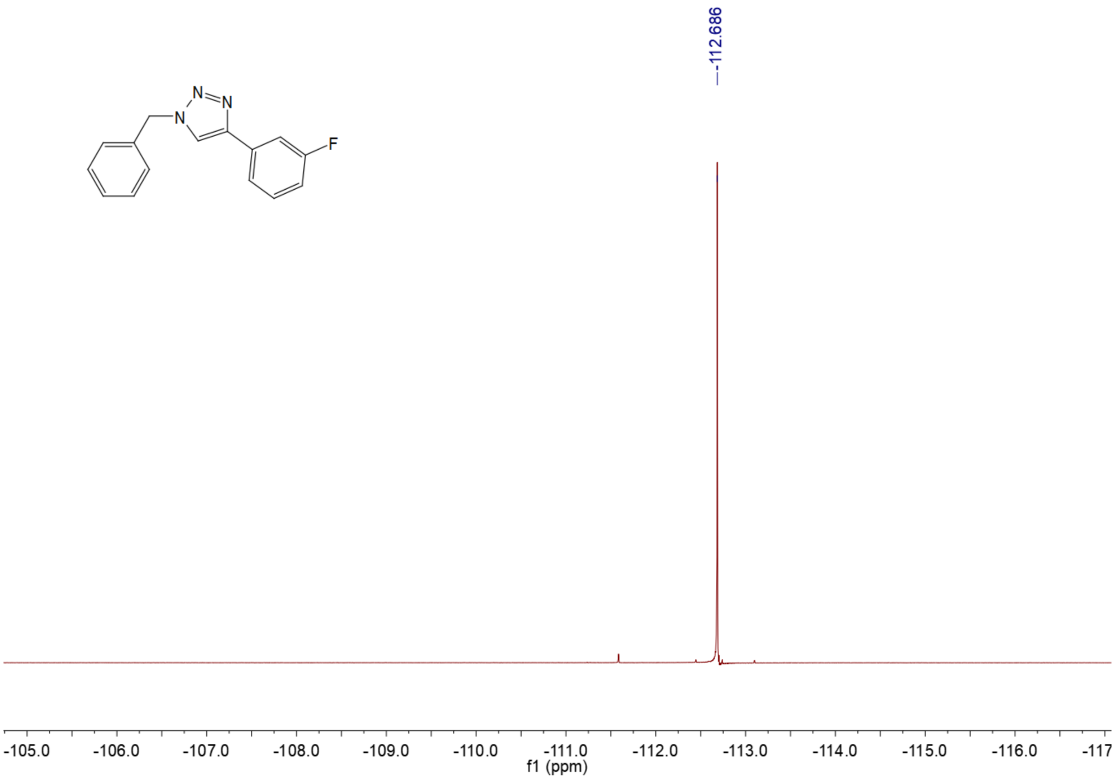

-112.686
N
N
N
F
-105.0
-106.0
-107.0
-108.0
-109.0
-110.0
-111.0
-112.0
-113.0
-114.0
-115.0
-116.0
-117
f1 (ppm)

Benzyl-4-(3-bromophenyl)-1*H*-1,2,3-triazole (4k)

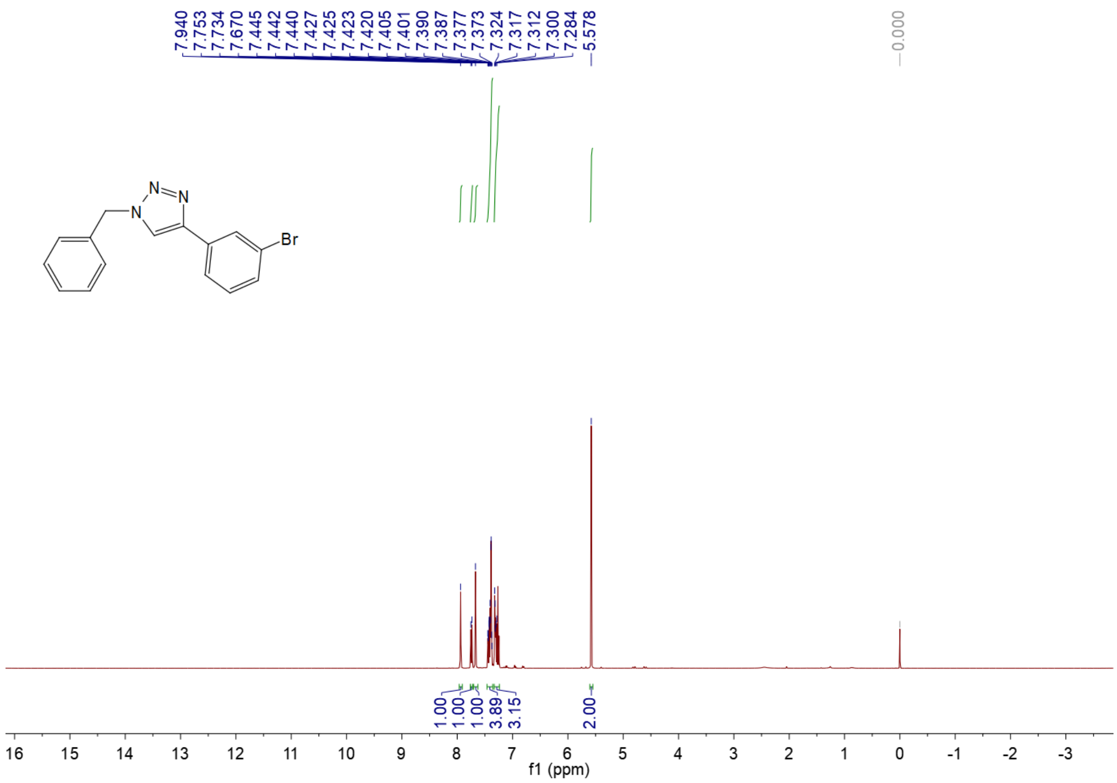

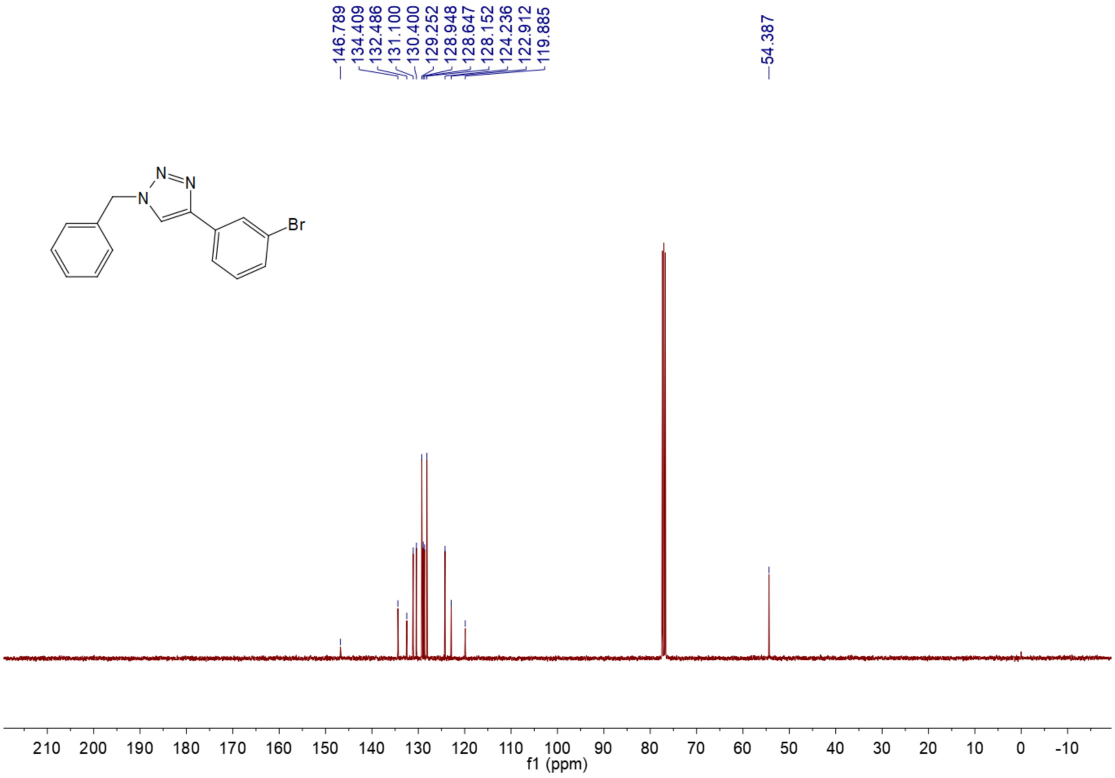

2-(1-Benzyl-1*H*-1,2,3-triazol-4-yl)propan-2-ol (4l)

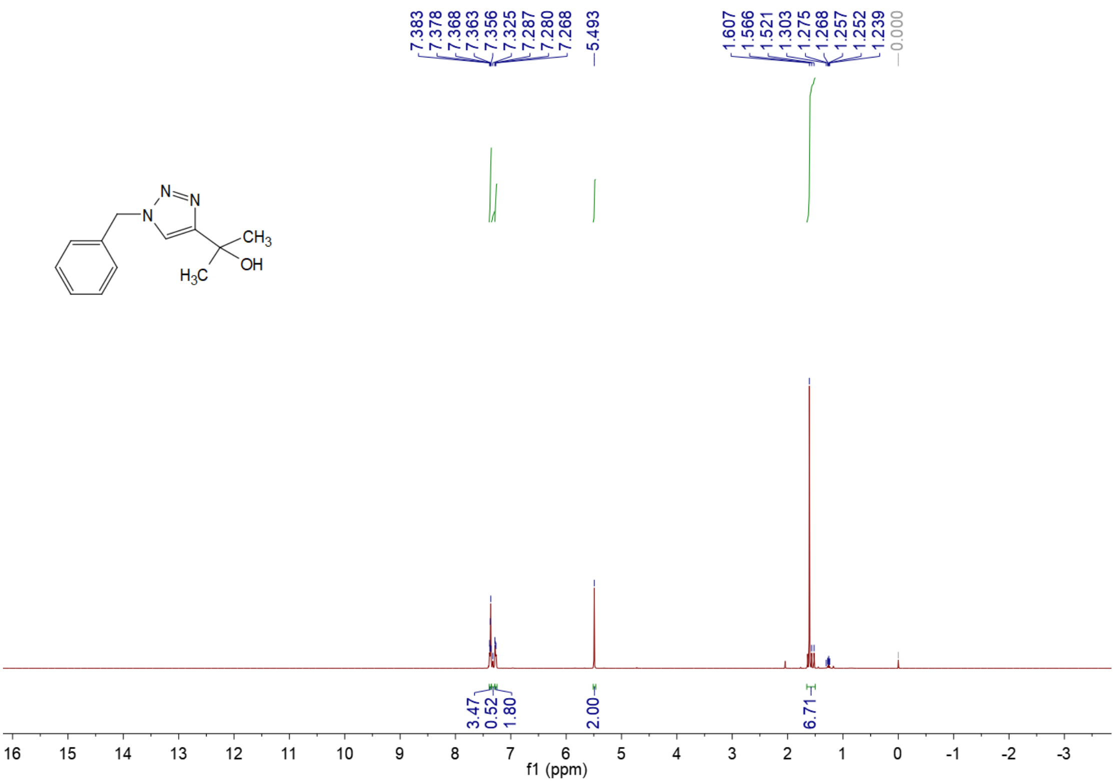

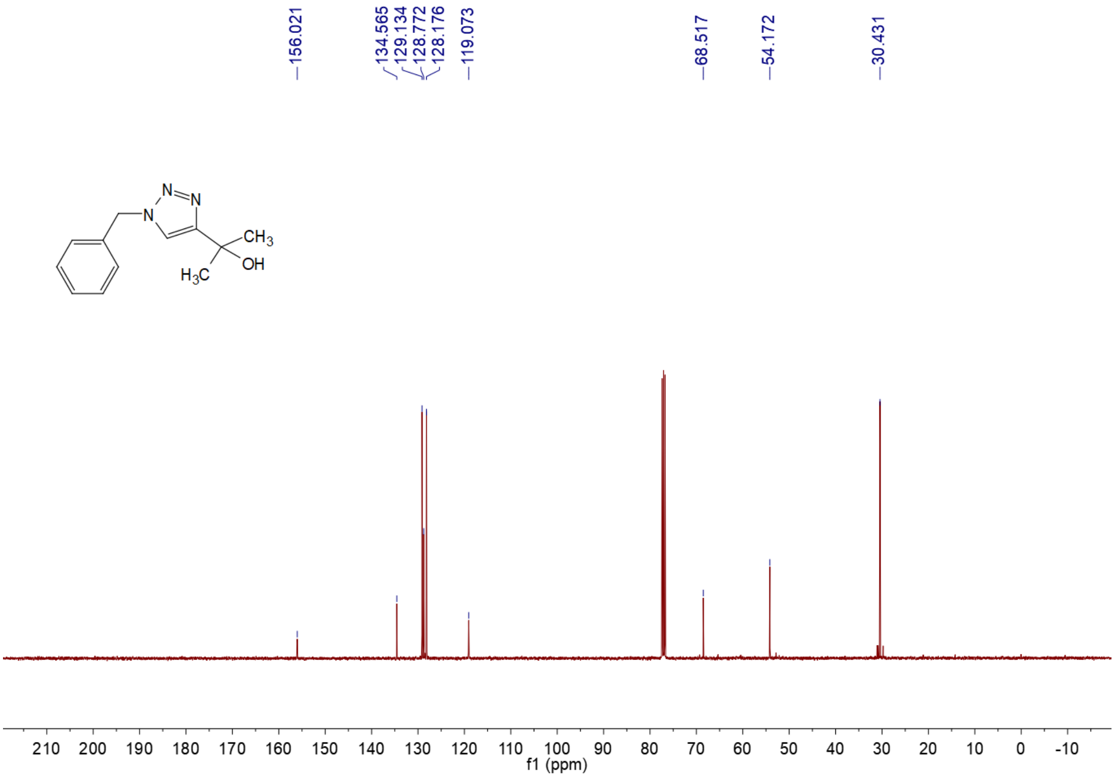

1-Benzyl-4-butyl-1*H*-1,2,3-triazole (4m)

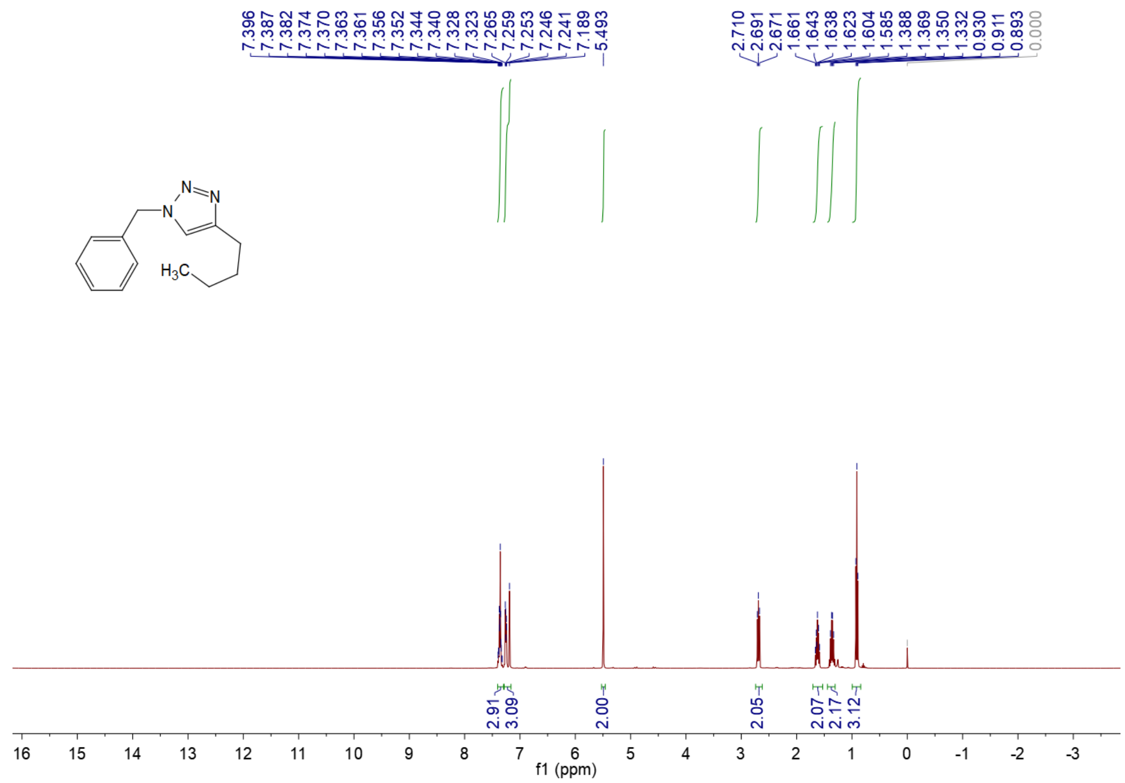

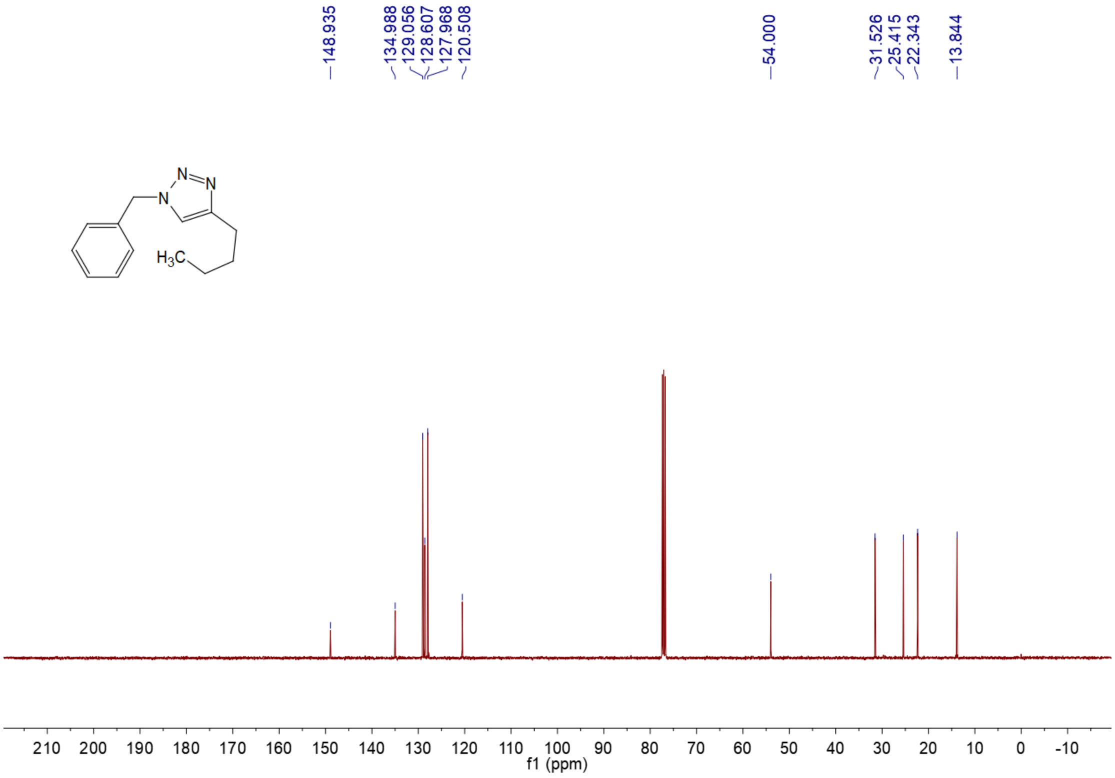

1-(4-Methylbenzyl)-4-phenyl-1*H*-1,2,3-triazole (4n)

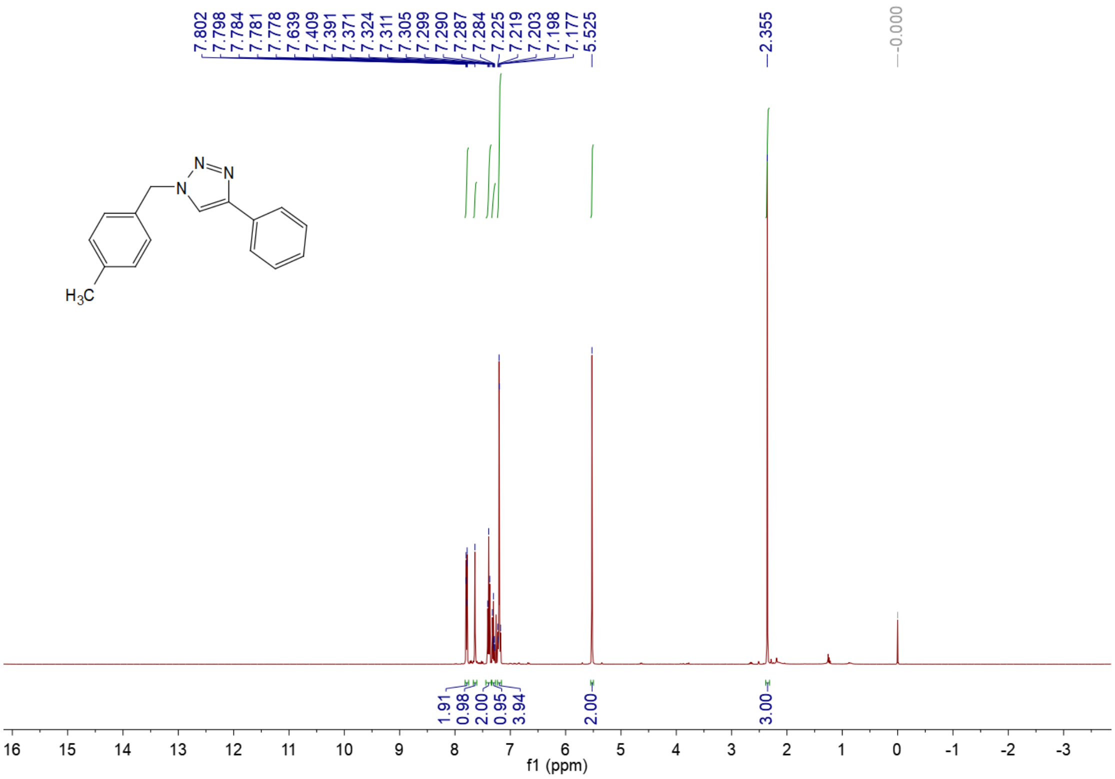

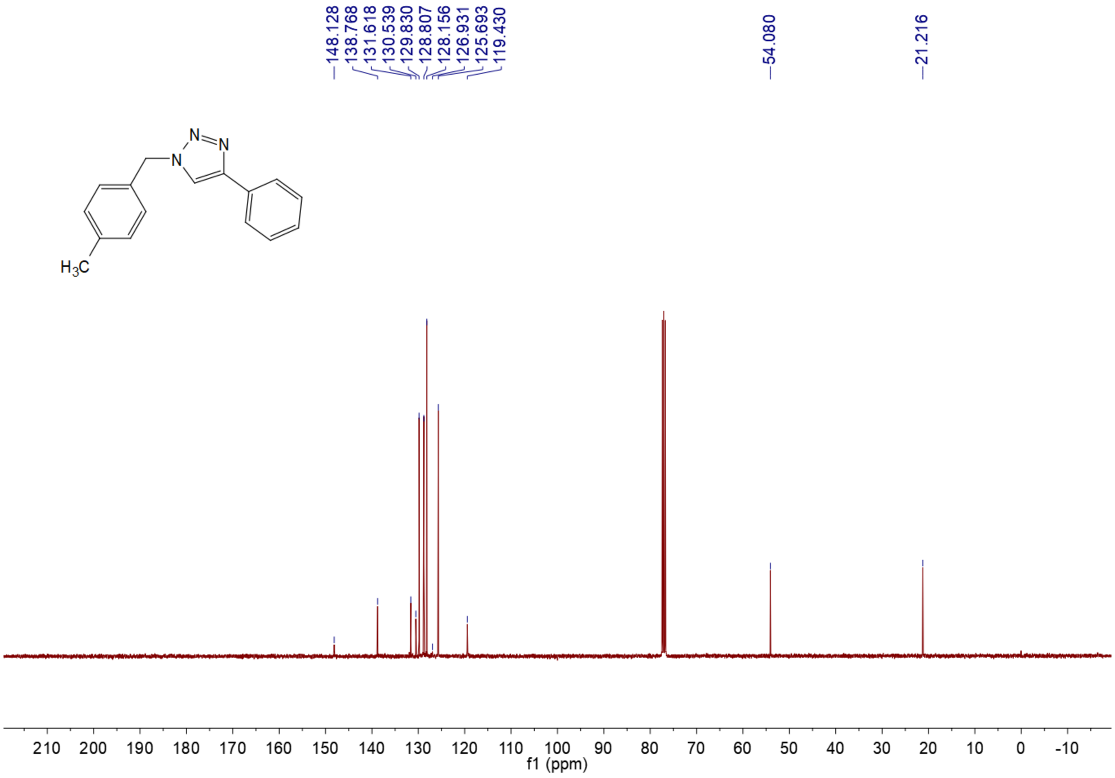

1-(4-Methylbenzyl)-4-phenyl-1*H*-1,2,3-triazole (4o)

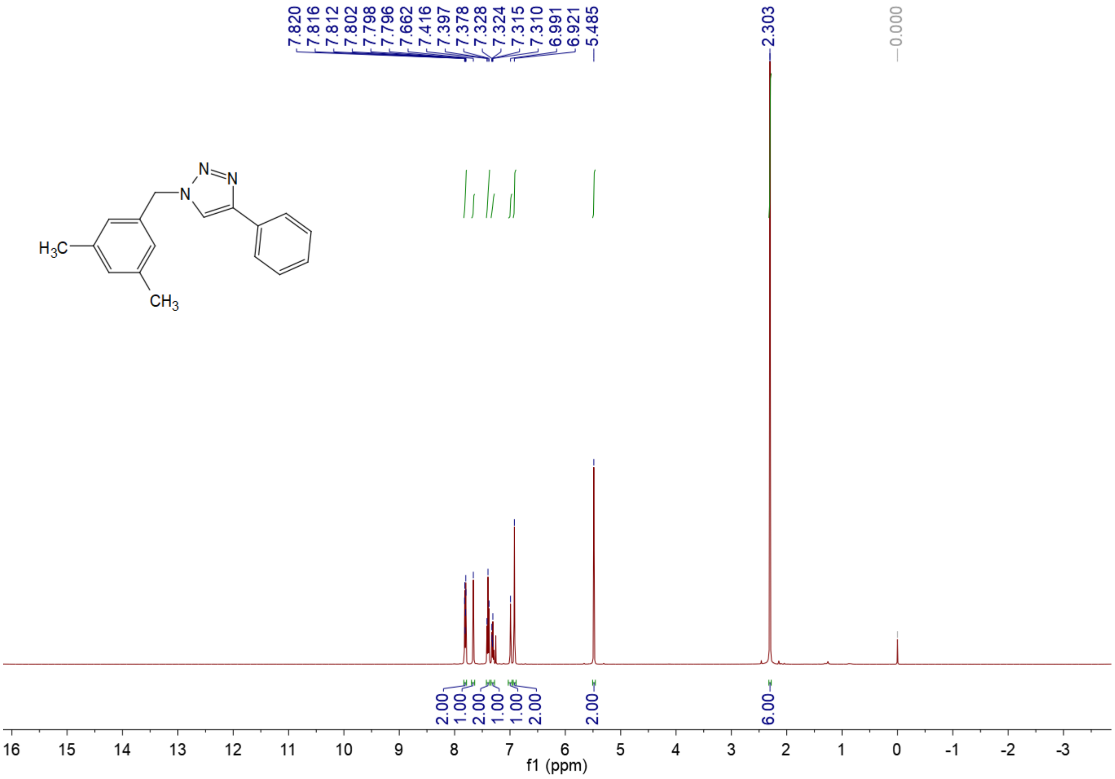

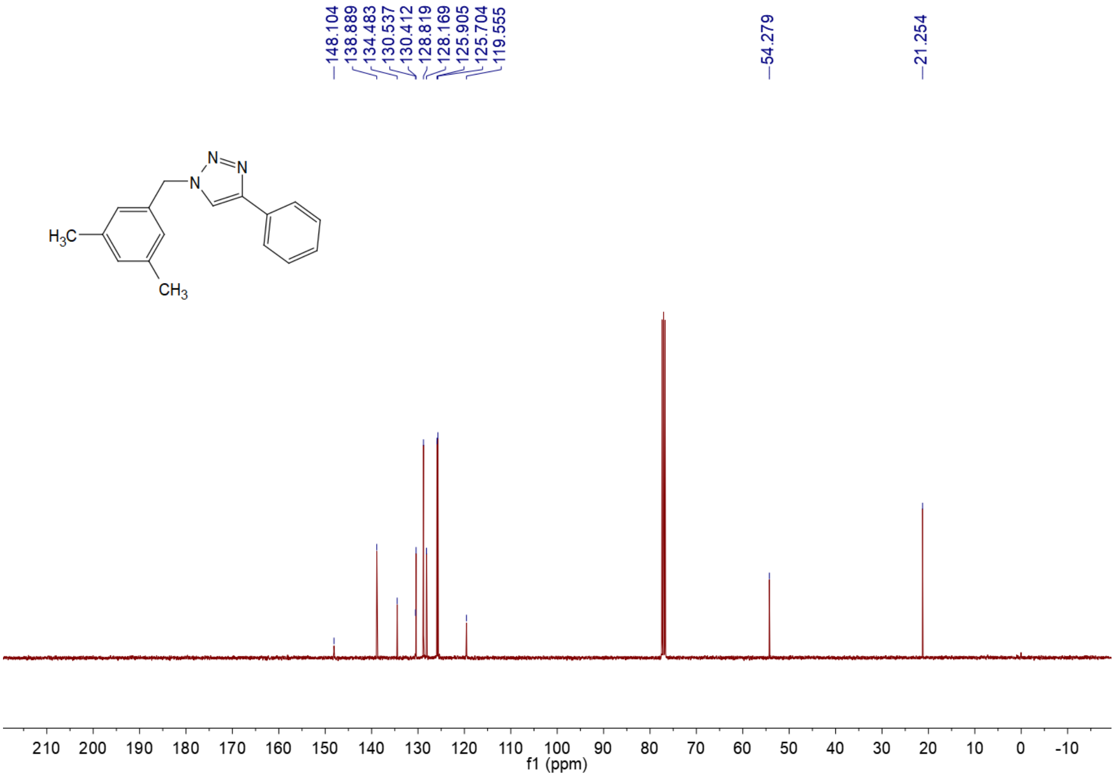

1-(3-Methoxybenzyl)-4-phenyl-1*H*-1,2,3-triazole (4p)

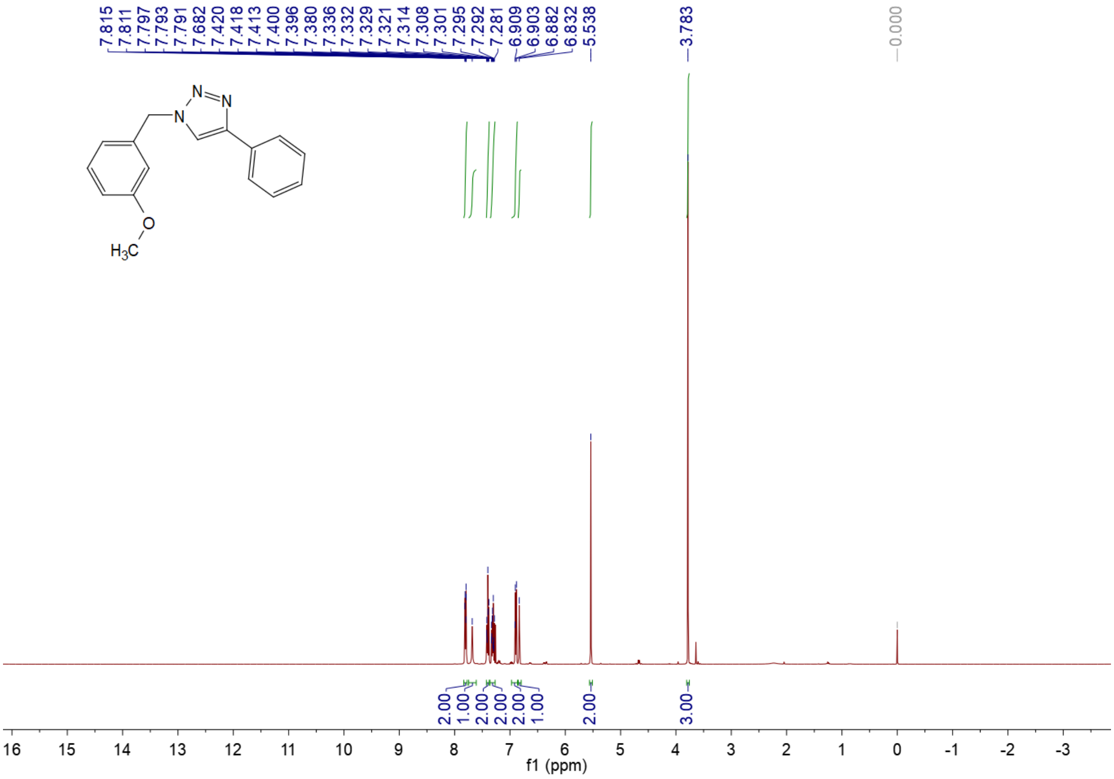

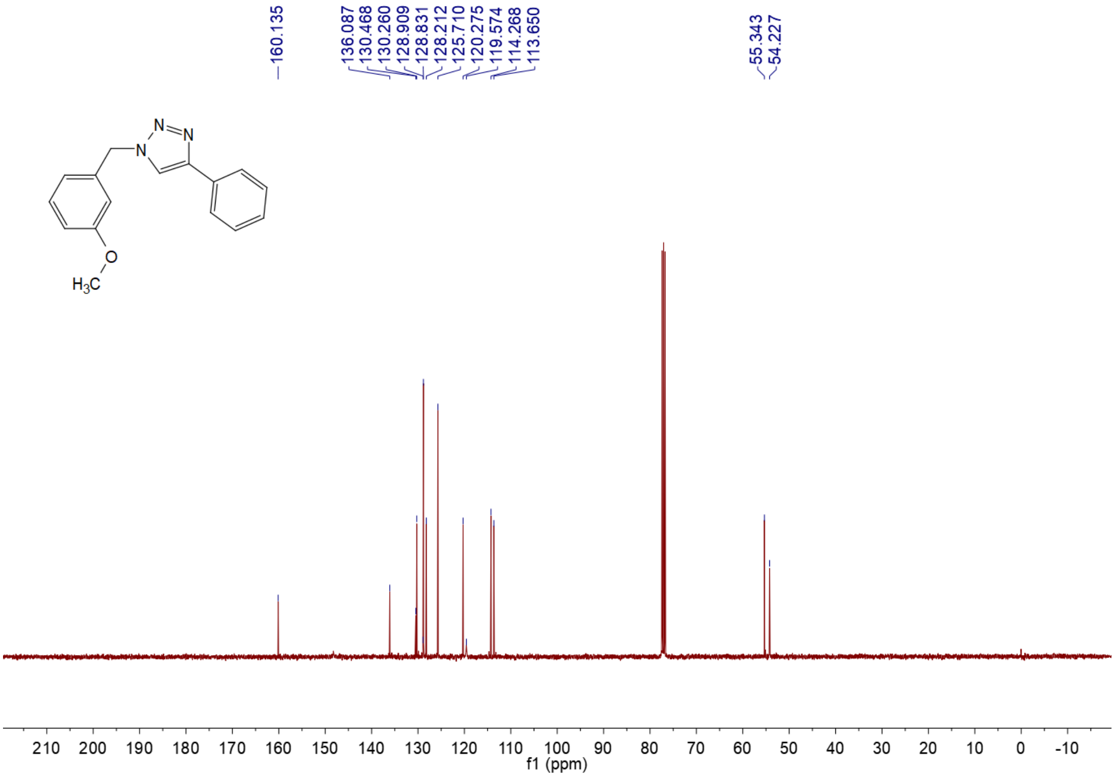

1-(3,5-Dimethoxybenzyl)-4-phenyl-1*H*-1,2,3-triazole (4q)

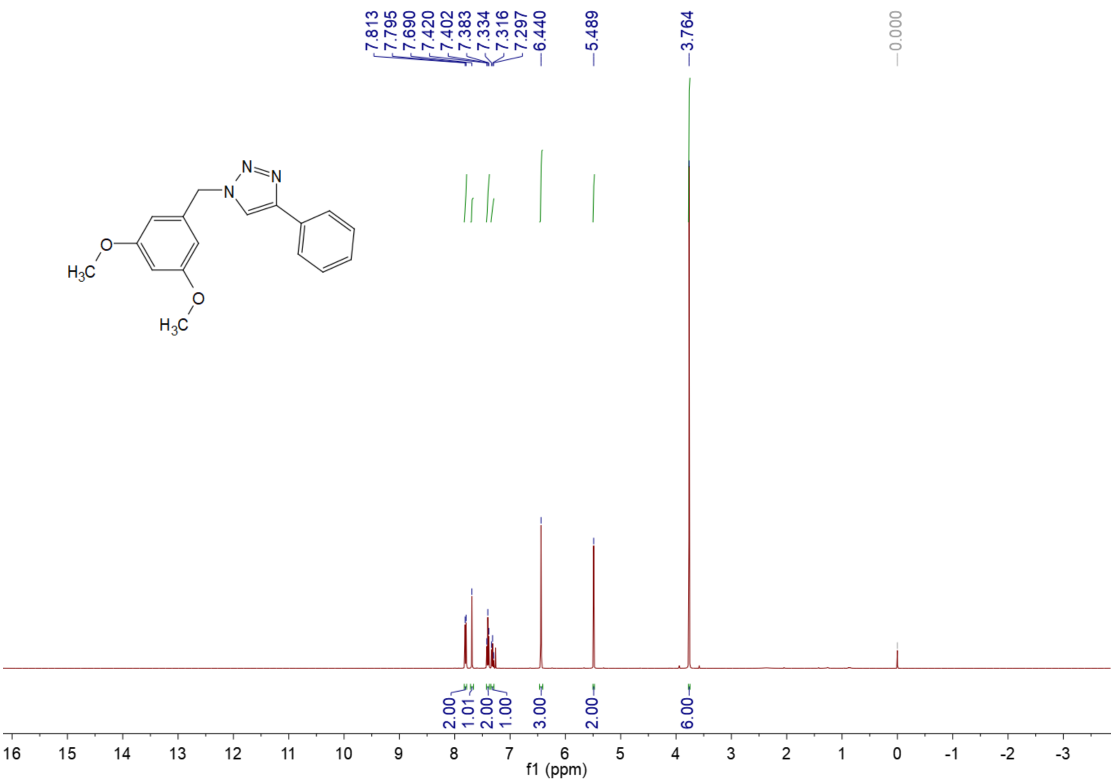

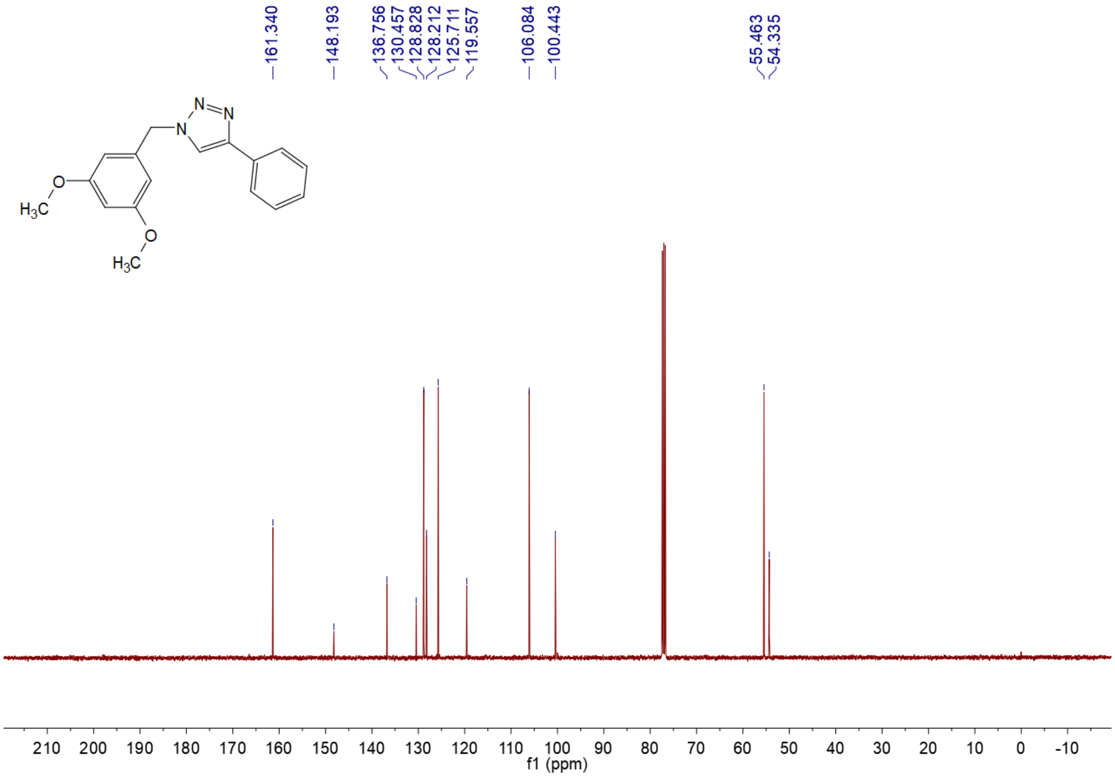

1-(4-(Tert-butyl)benzyl)-4-phenyl-1*H*-1,2,3-triazole (4r)

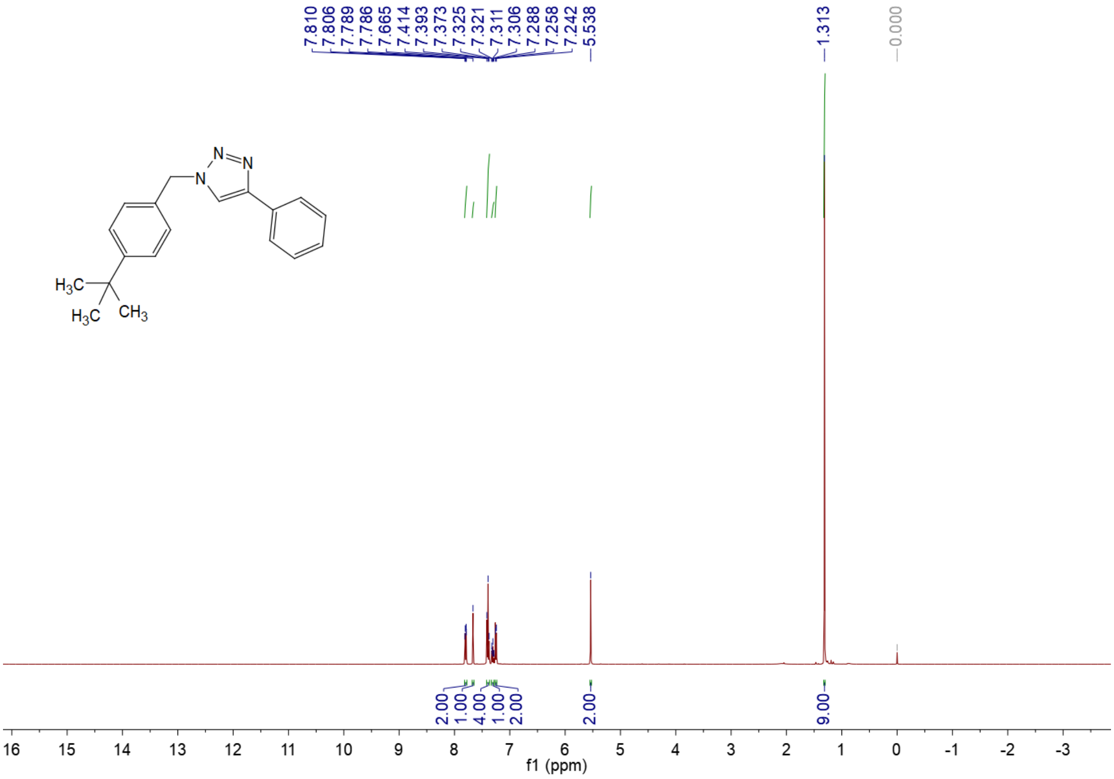

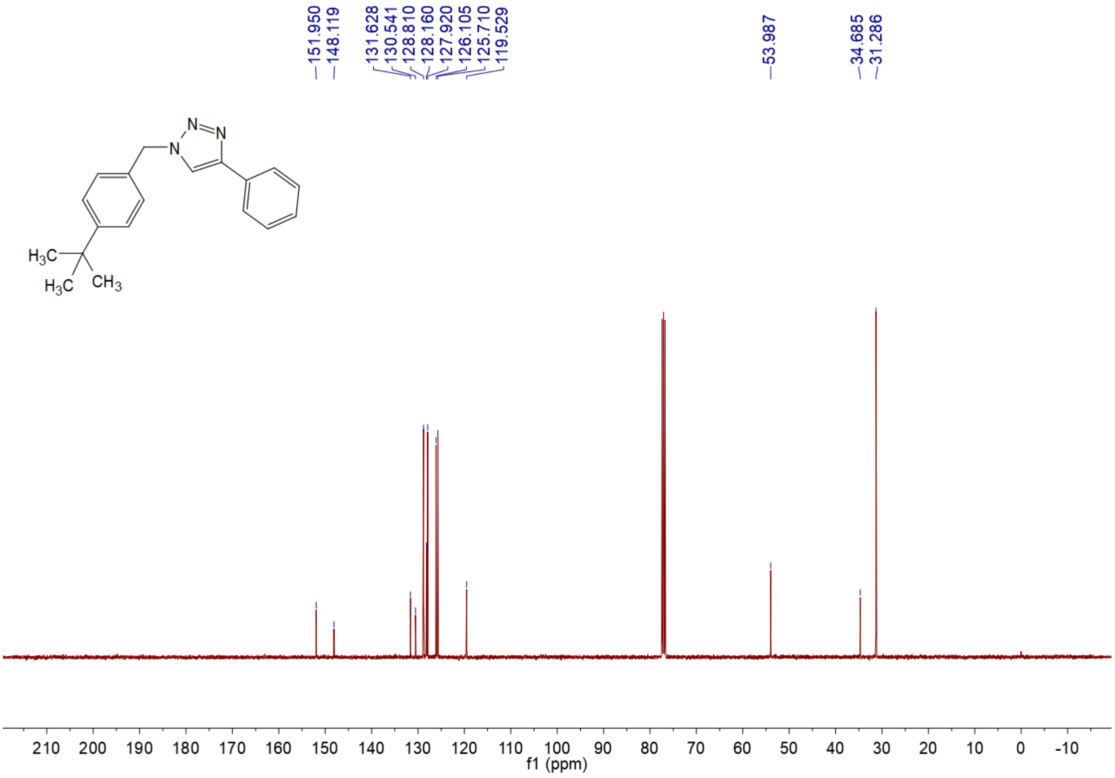

1-(4-Fluorobenzyl)-4-phenyl-1H-1,2,3-triazole (4s)

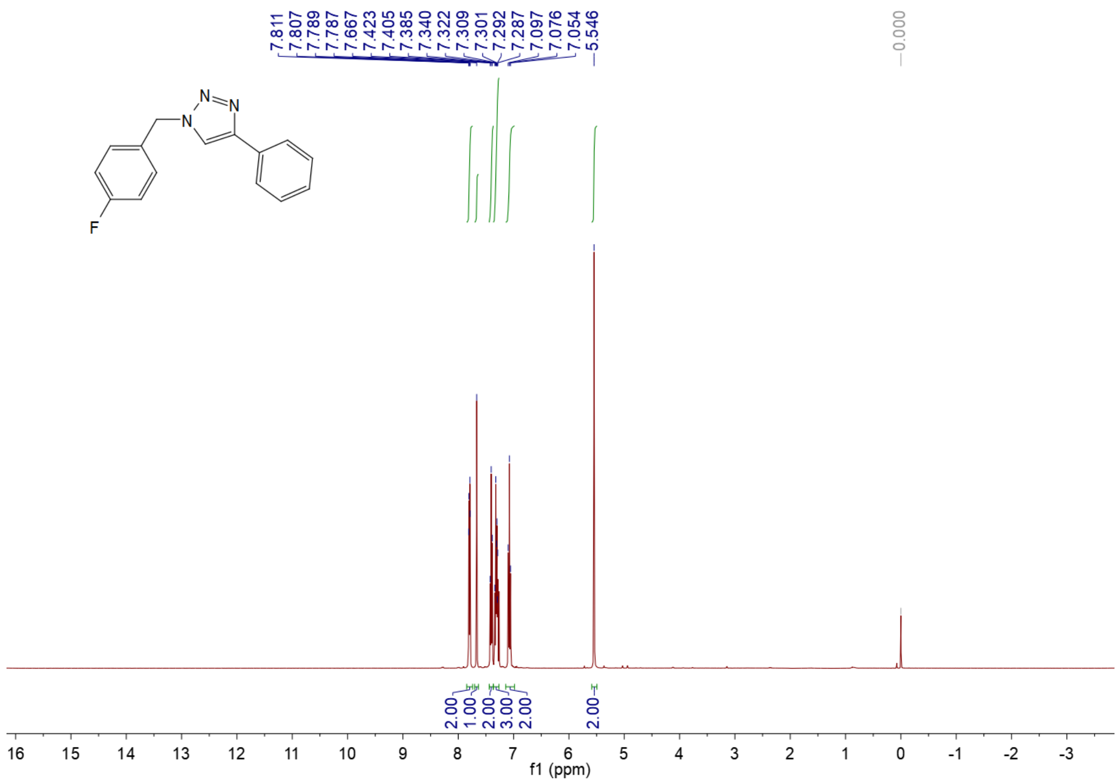

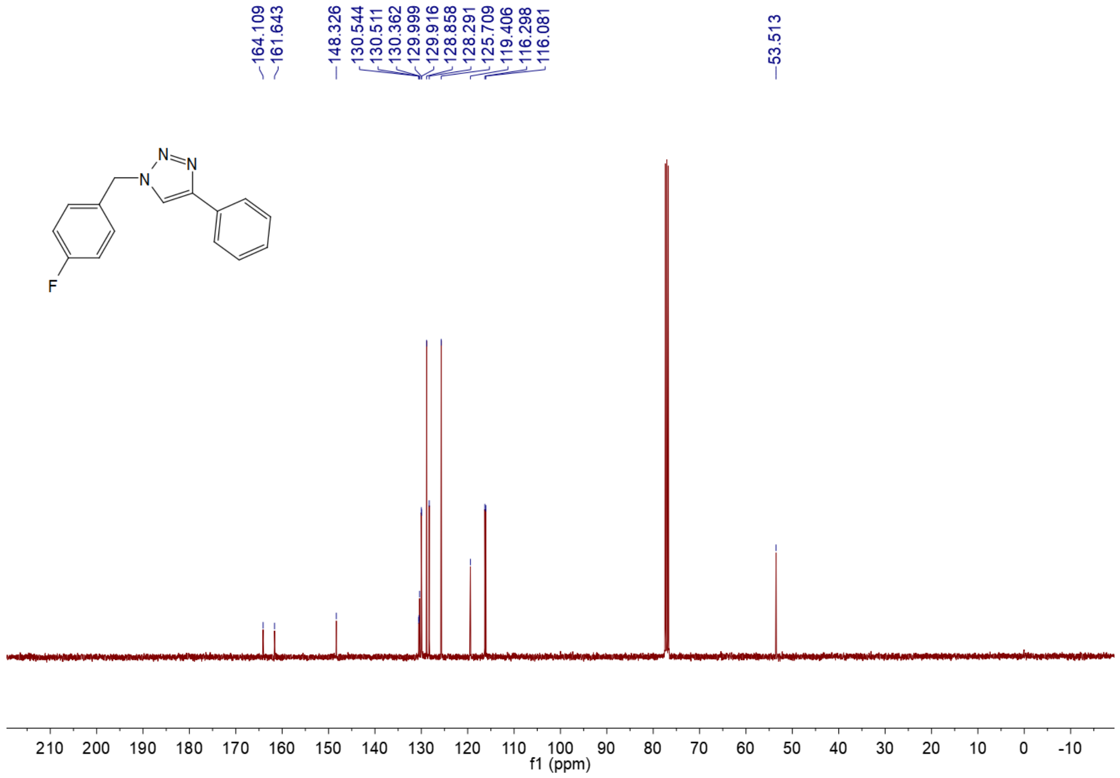

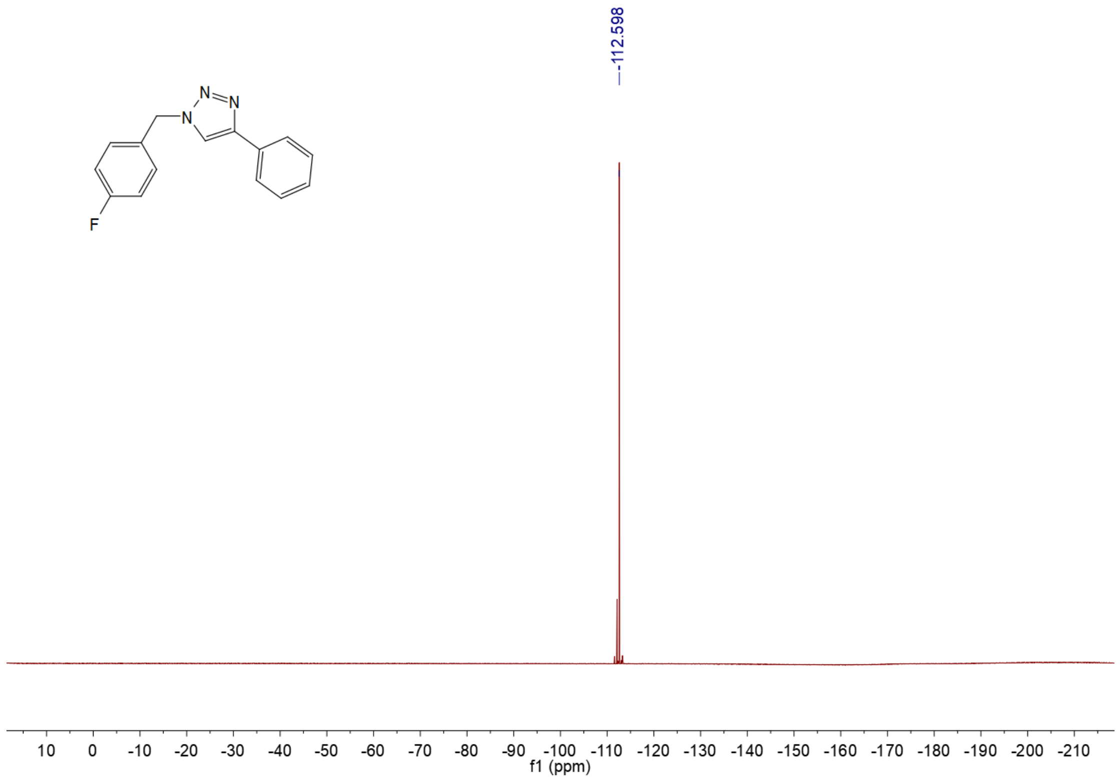

-112.598
N
N
N
F
10
0
-10
-20
-30
-40
-50
-60
-70
-80
-90
-100
-110
-120
-130
-140
-150
-160
-170
-180
-190
-200
-210
f1 (ppm)

1-(4-Chlorobenzyl)-4-phenyl-1*H*-1,2,3-triazole (4t)

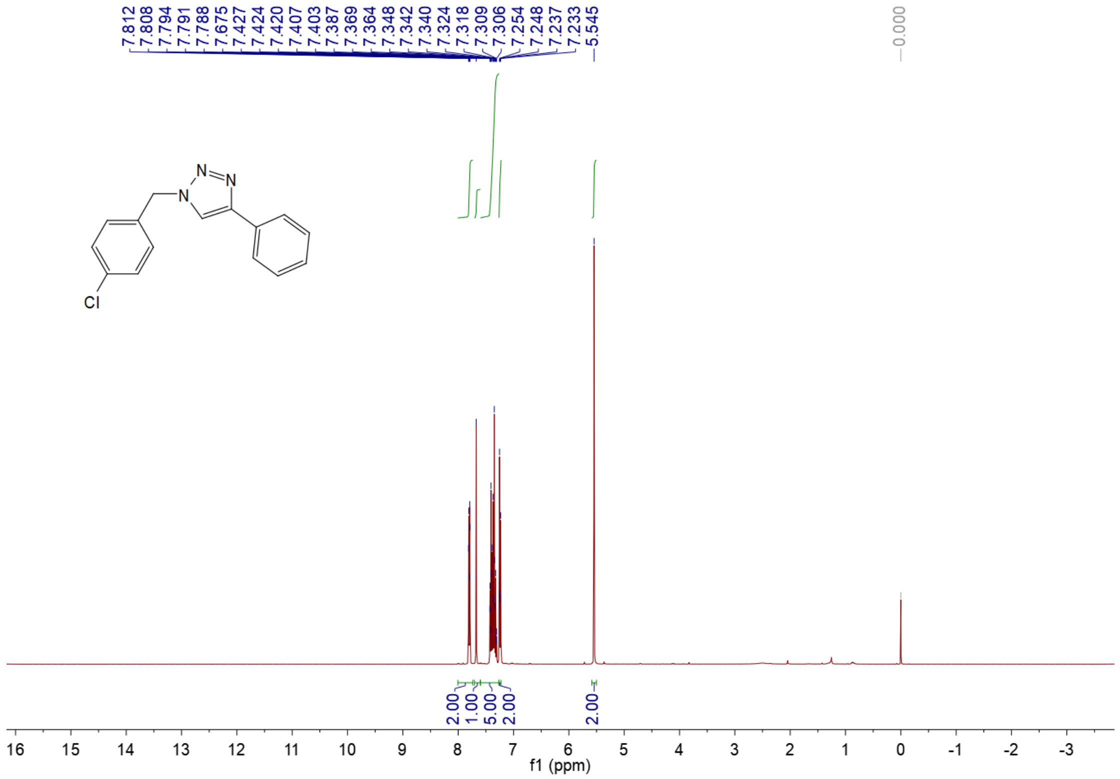

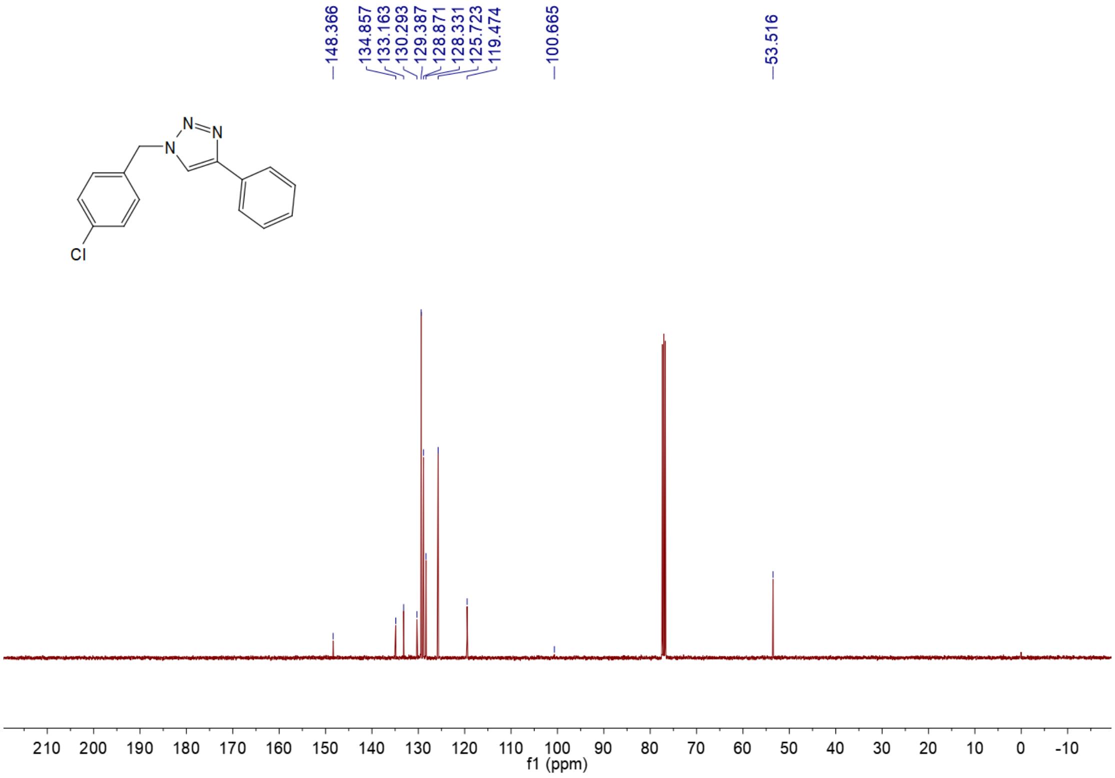

1-(4-Bromobenzyl)-4-phenyl-1*H*-1,2,3-triazole (4u)

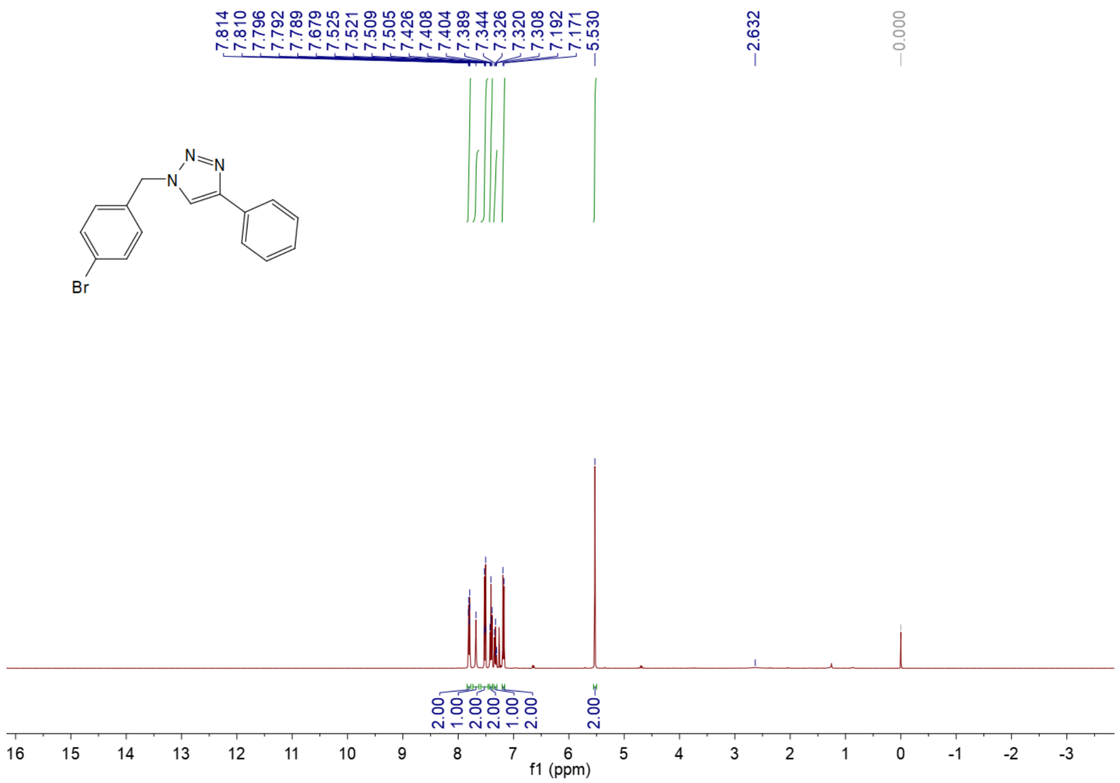

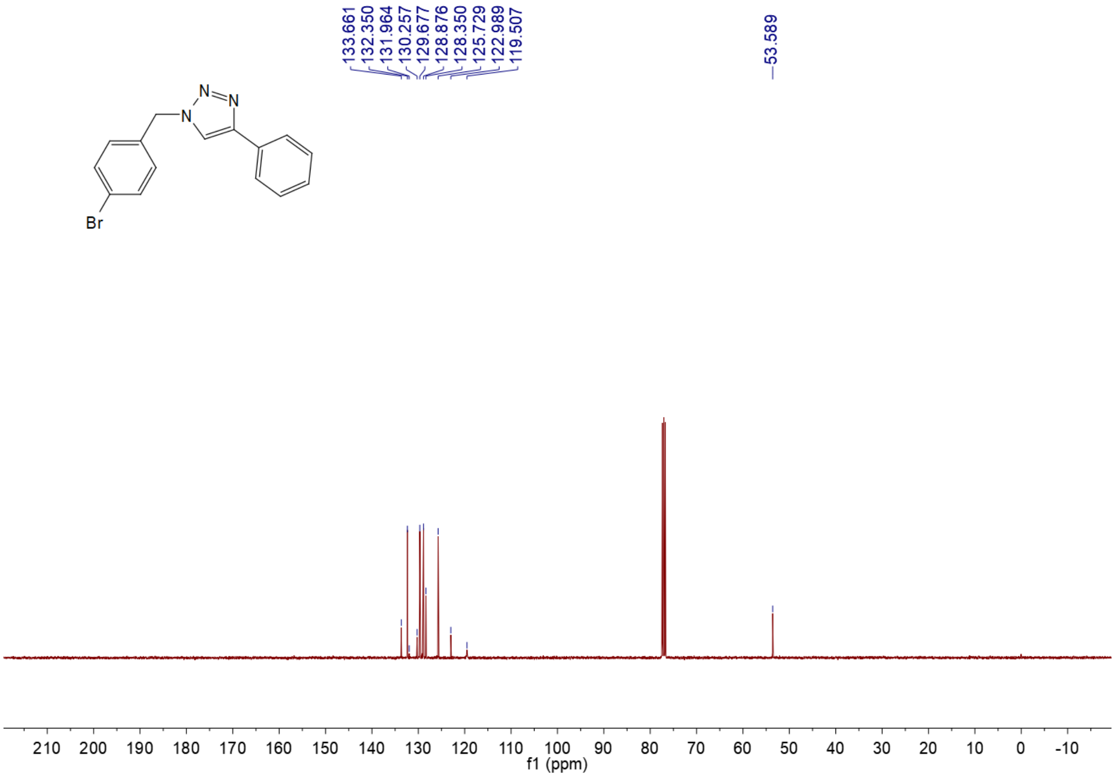

1-(4-Nitrobenzyl)-4-phenyl-1*H*-1,2,3-triazole (4v)

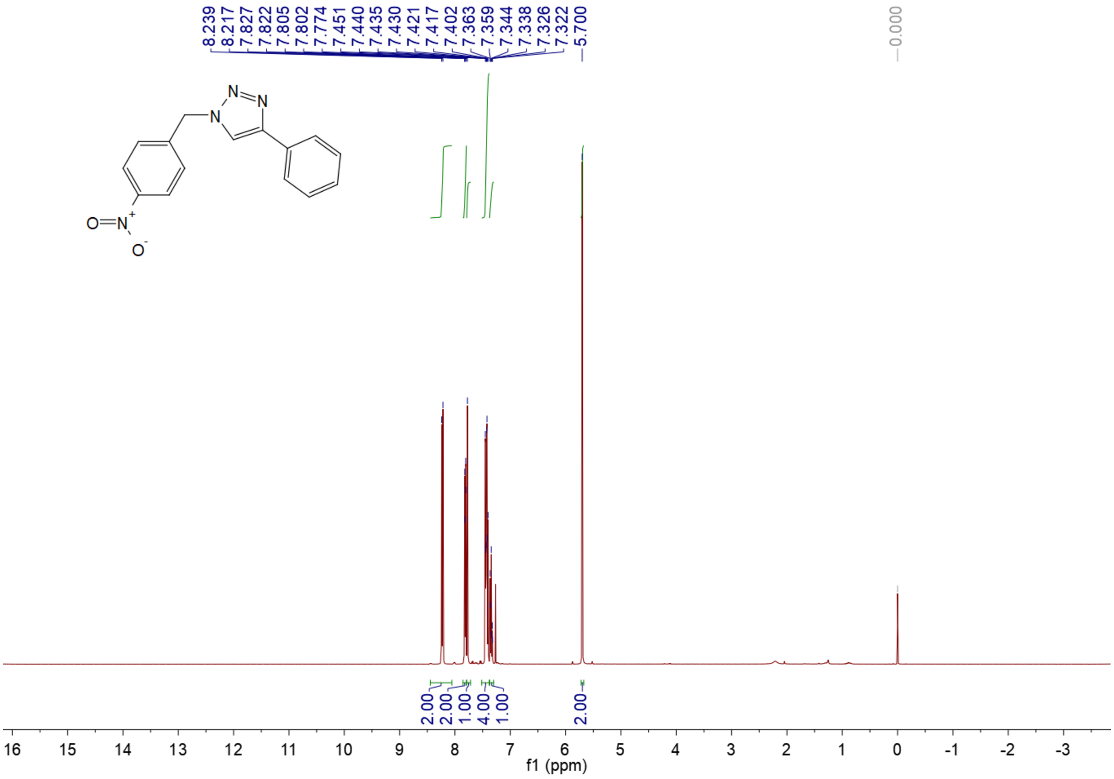

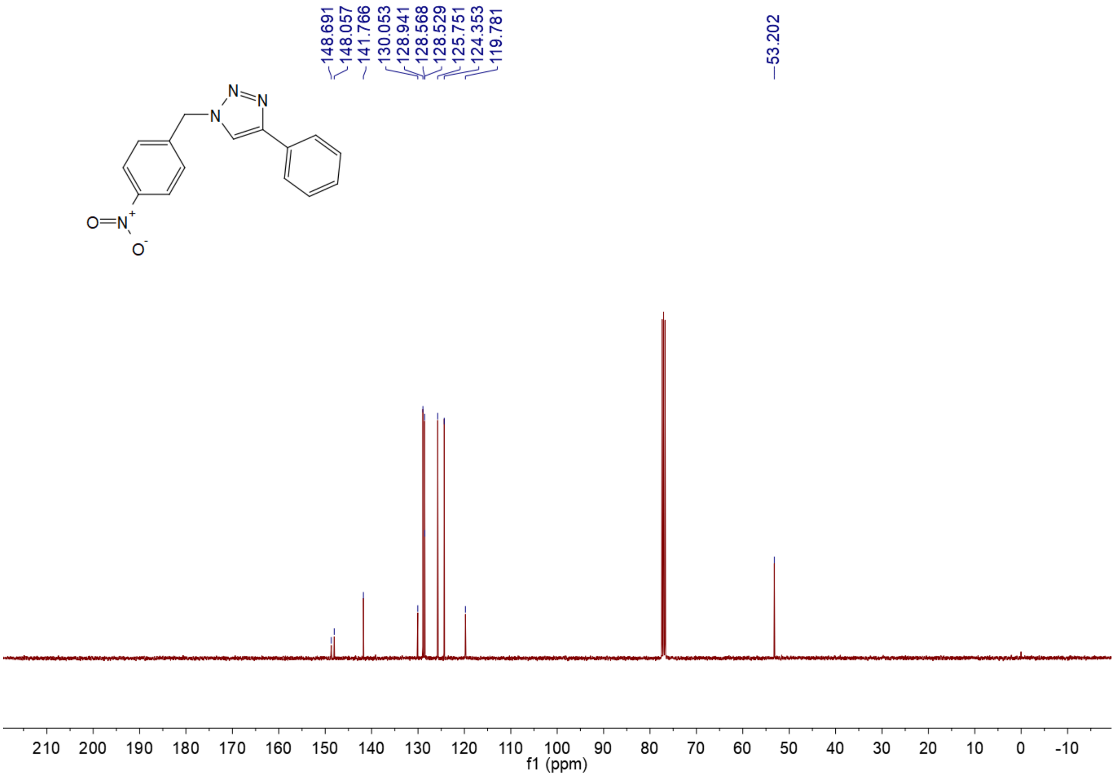

4-Phenyl-1-(4-(trifluoromethyl)benzyl)-1*H*-1,2,3-triazole (4w)

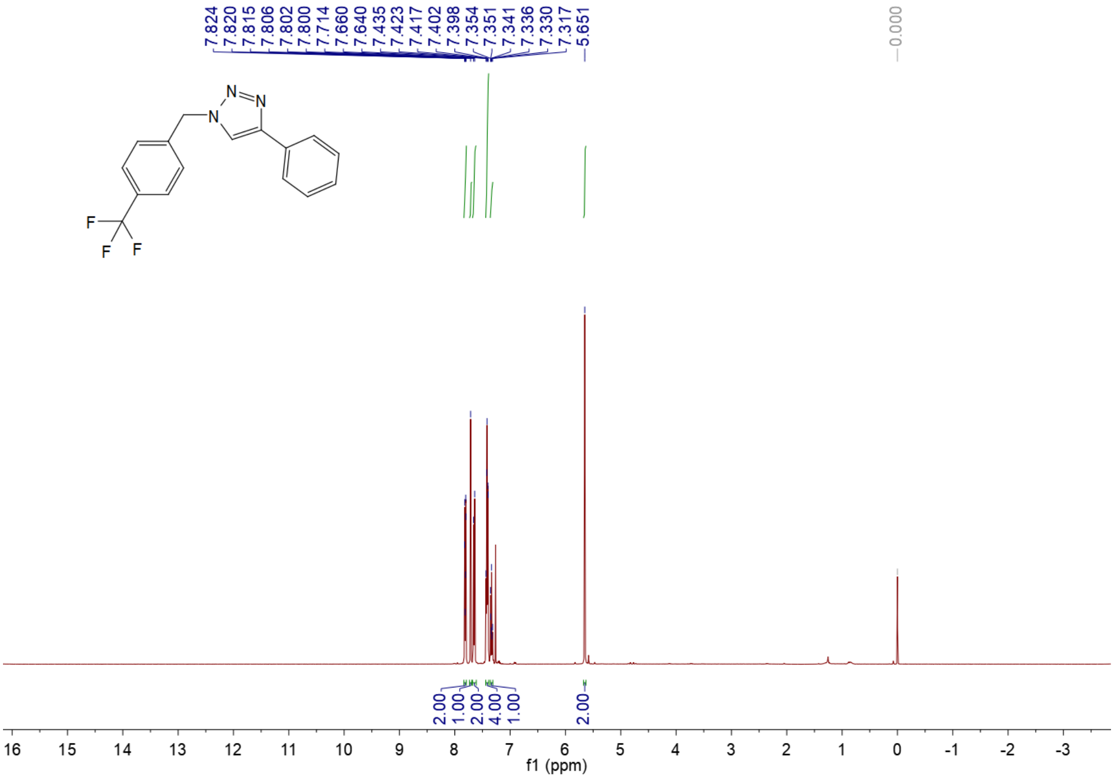

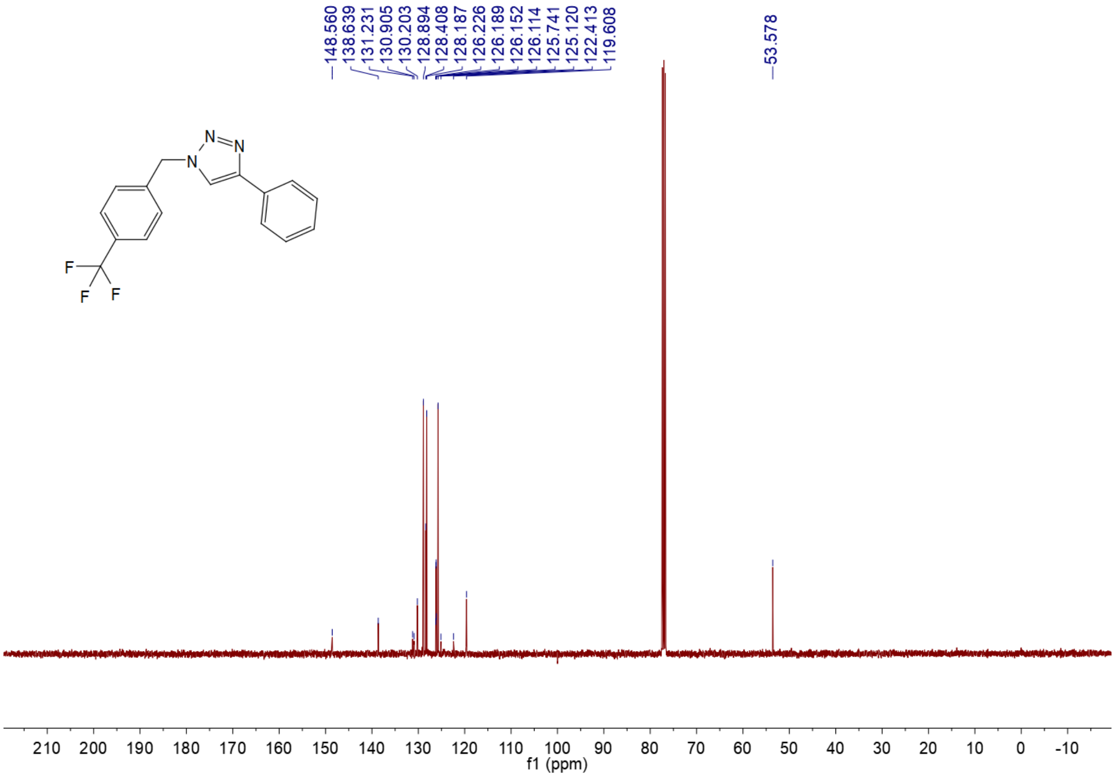

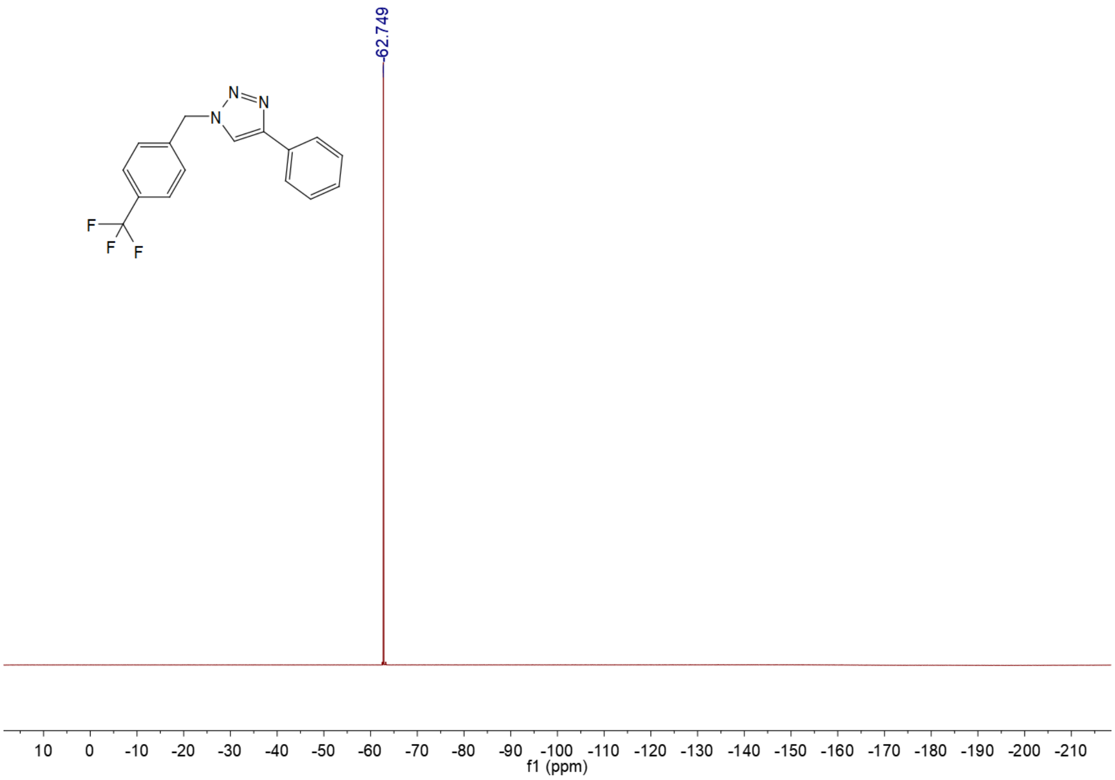
-62.749
N
N
N
F
F
F
10
0
-10
-20
-30
-40
-50
-60
-70
-80
-90
-100
-110
-120
-130
-140
-150
-160
-170
-180
-190
-200
-210
f1 (ppm)

1-(2-Fluorobenzyl)-4-phenyl-1*H*-1,2,3-triazole (4x)

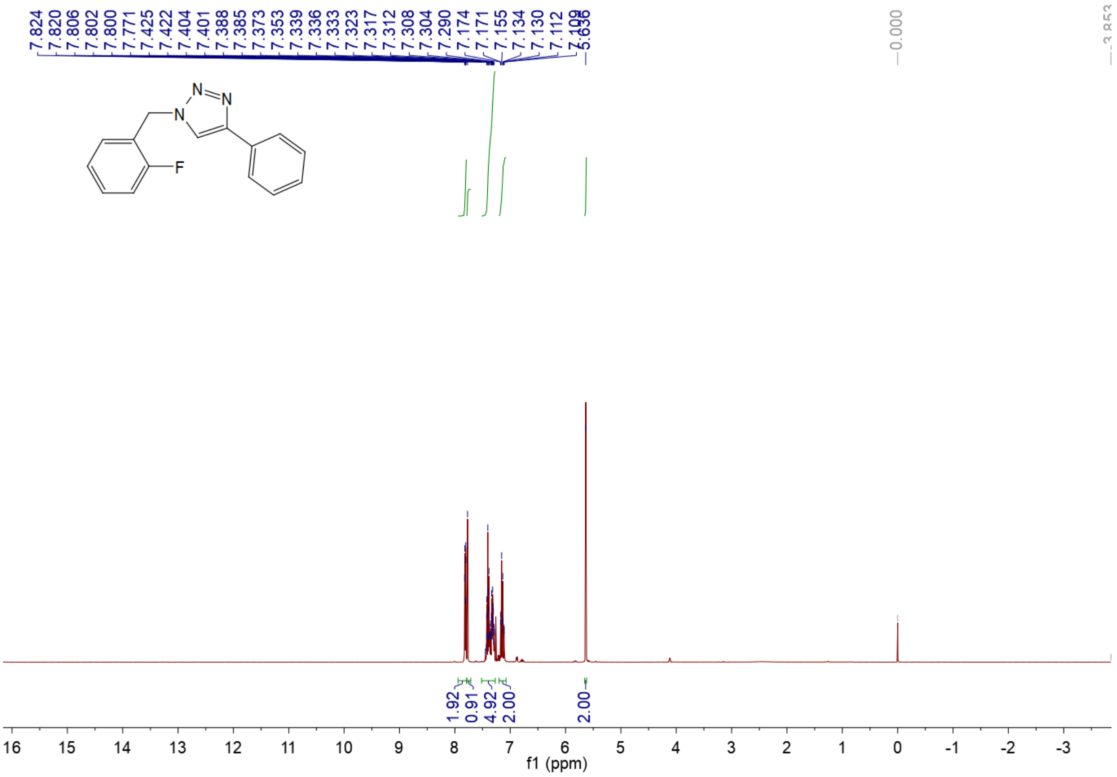

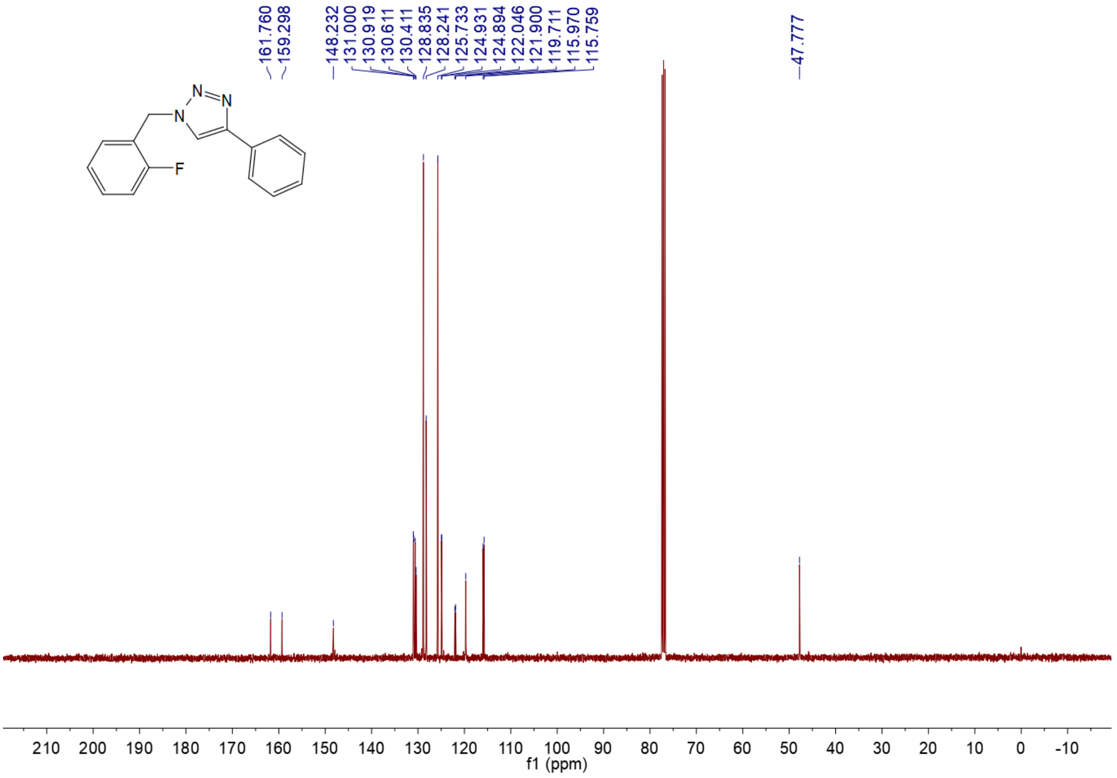

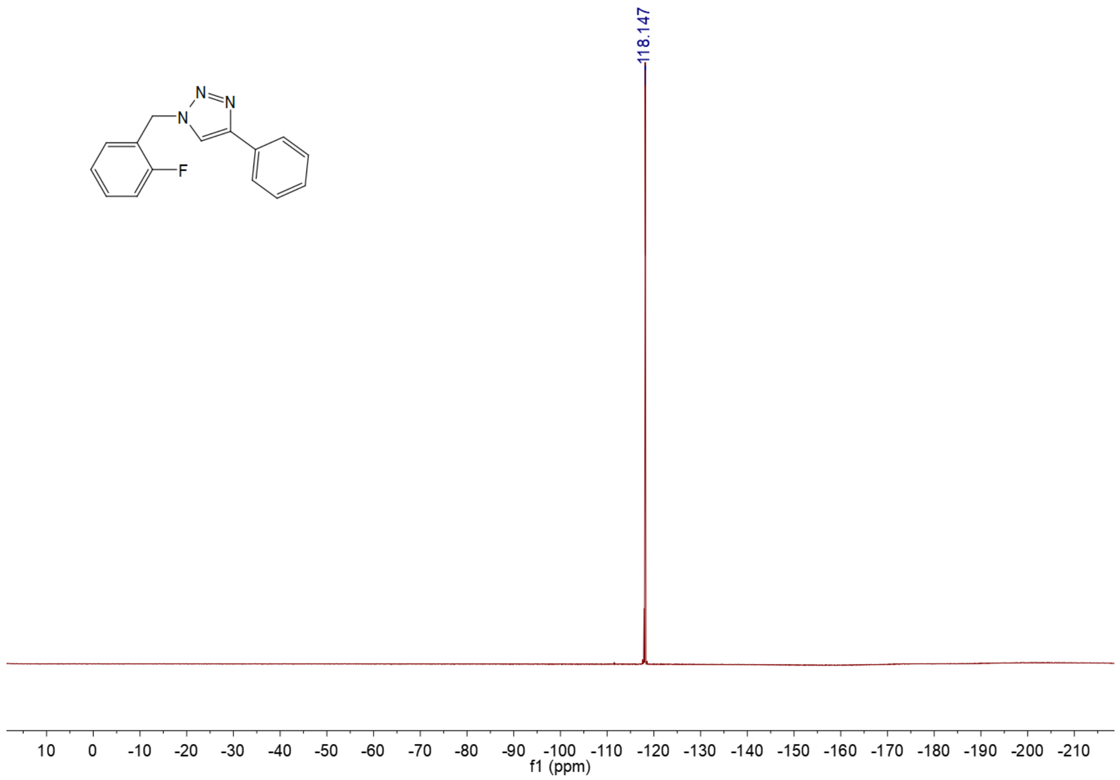

N
N
N
F
-118.147
10 0 -10 -20 -30 -40 -50 -60 -70 -80 -90 -100 -110 -120 -130 -140 -150 -160 -170 -180 -190 -200 -210
f1 (ppm)

1-(2-Chlorobenzyl)-4-phenyl-1*H*-1,2,3-triazole (4y)

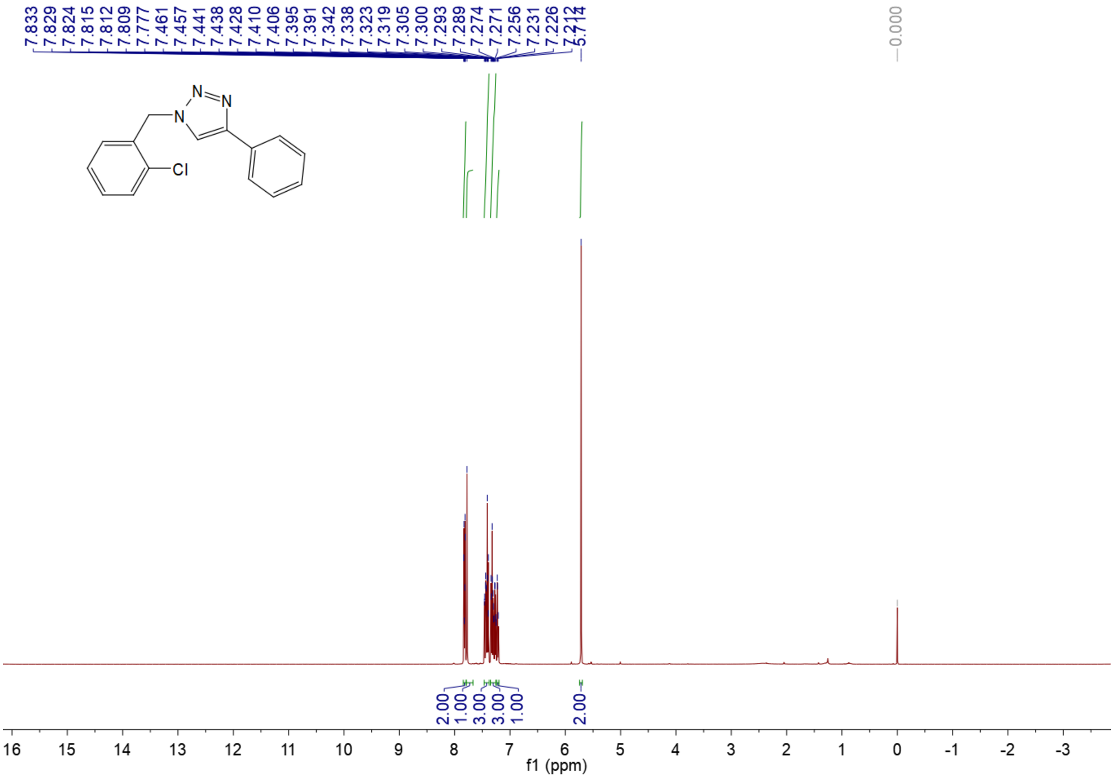

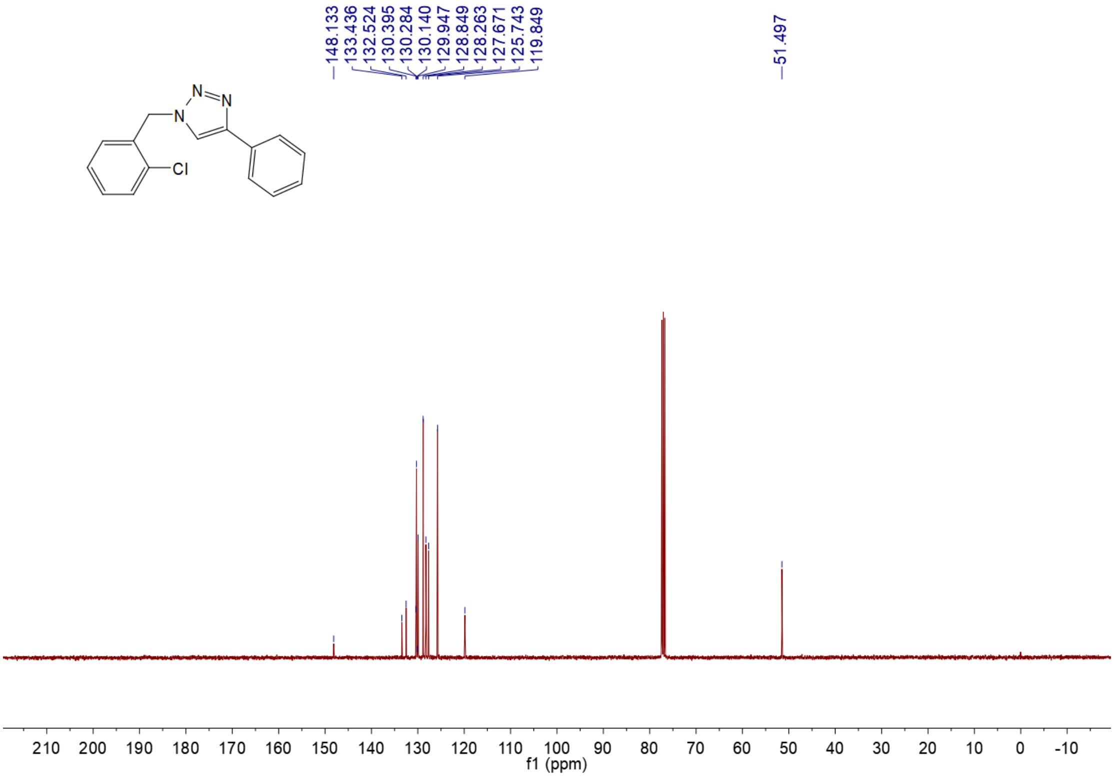

1-(2,5-Difluorobenzyl)-4-phenyl-1*H*-1,2,3-triazole (4z)

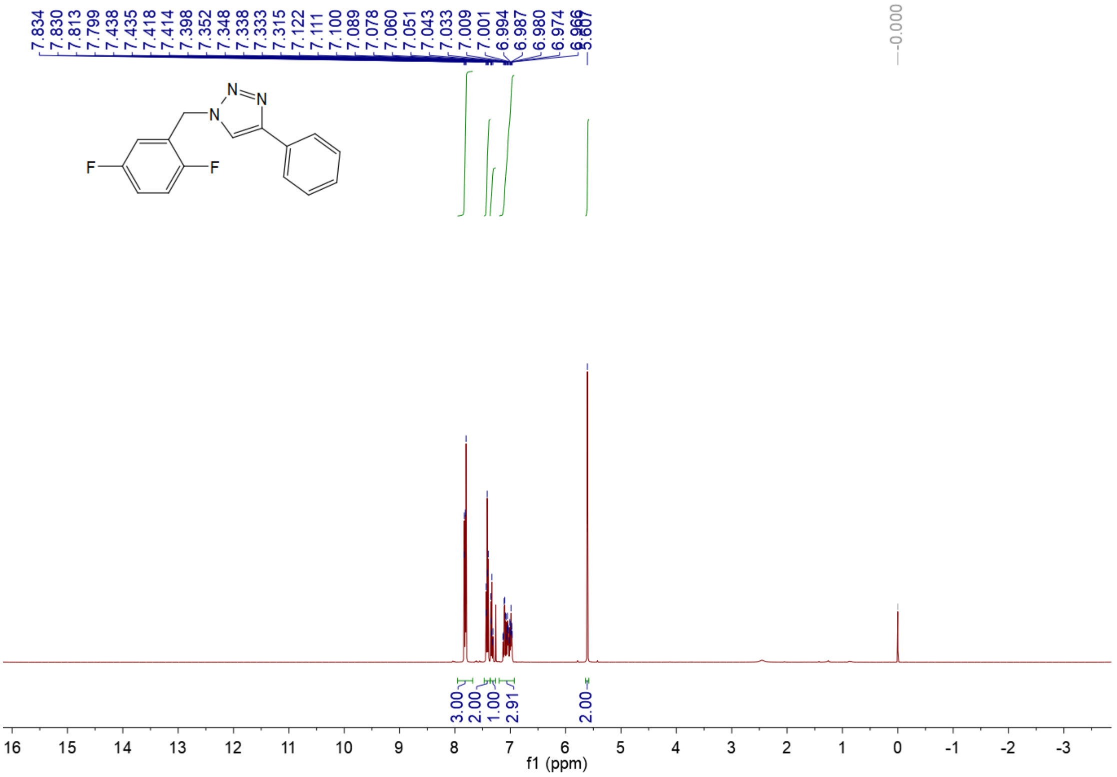

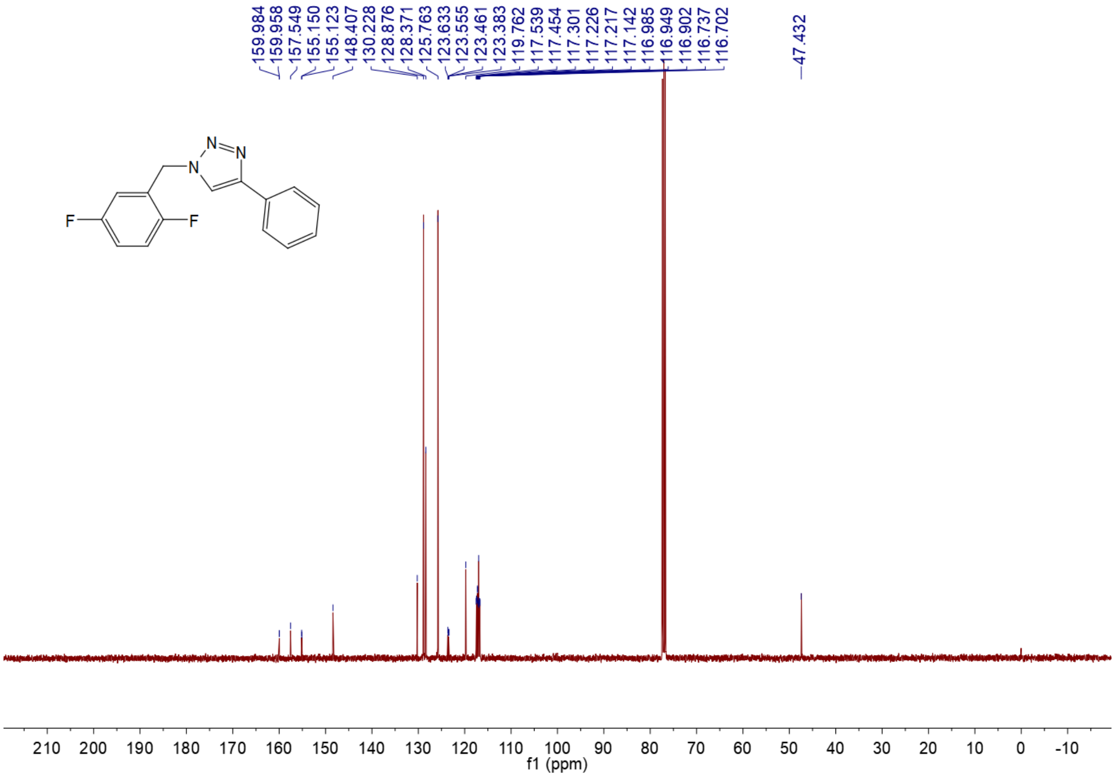

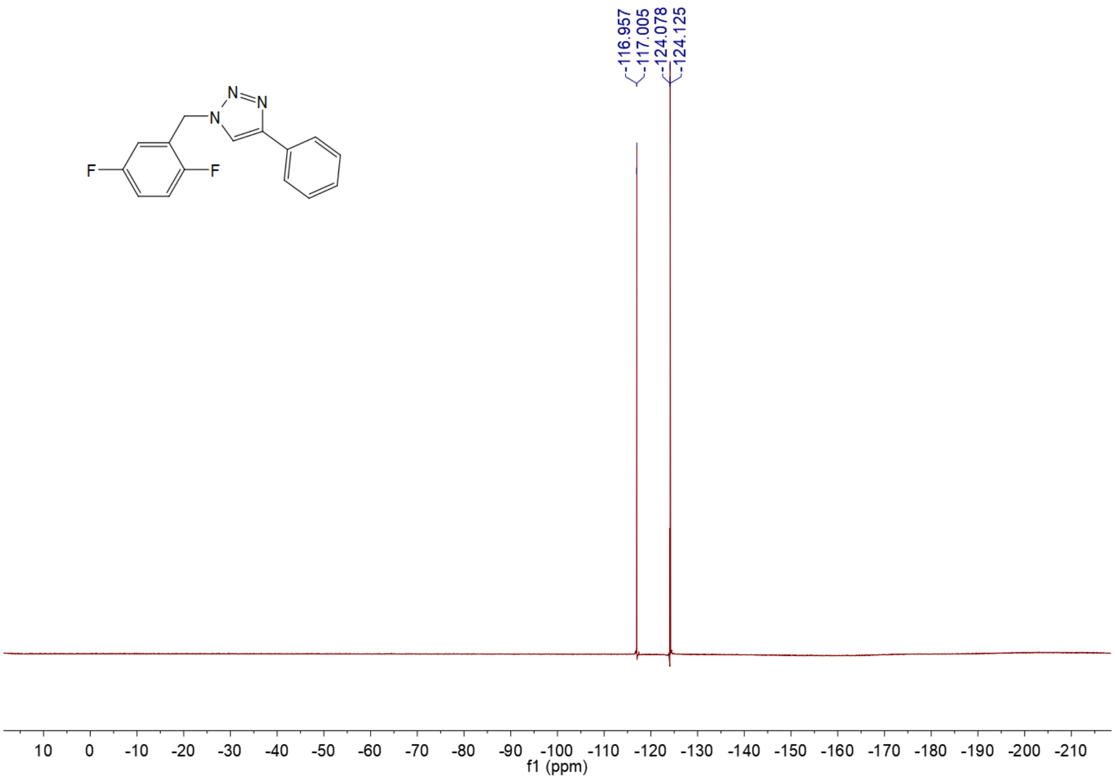

-116.957
-117.005
-124.078
-124.125
F
F
N
N
N
10 0 -10 -20 -30 -40 -50 -60 -70 -80 -90 -100 -110 -120 -130 -140 -150 -160 -170 -180 -190 -200 -210
f1 (ppm)

1-(3-Fluorobenzyl)-4-phenyl-1*H*-1,2,3-triazole (4aa)

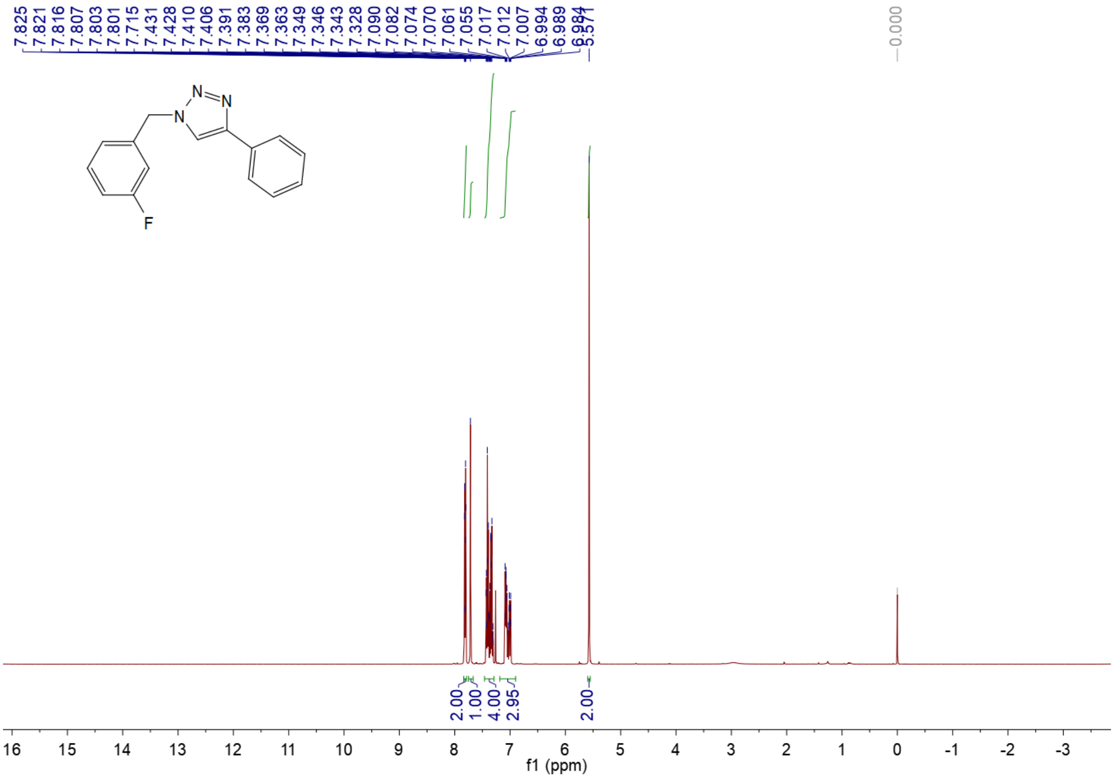

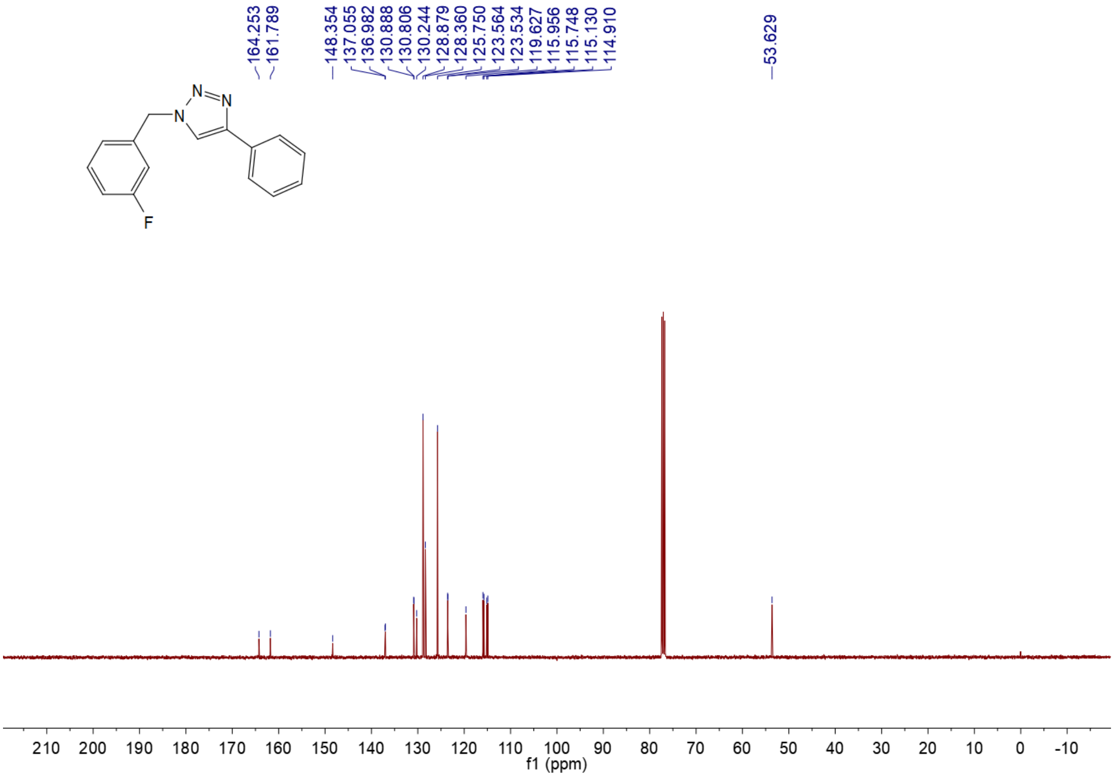

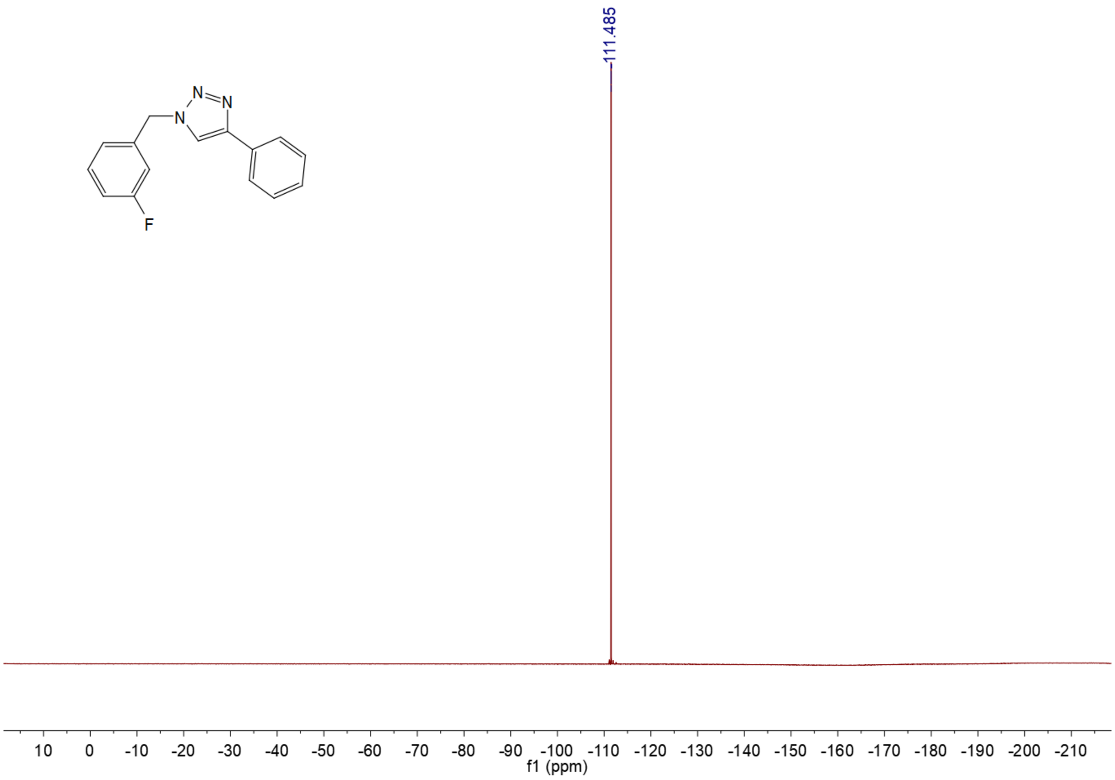

N
N
N
F
111.485
10 0 -10 -20 -30 -40 -50 -60 -70 -80 -90 -100 -110 -120 -130 -140 -150 -160 -170 -180 -190 -200 -210
f1 (ppm)

1-(3-Chlorobenzyl)-4-phenyl-1*H*-1,2,3-triazole (4ab)

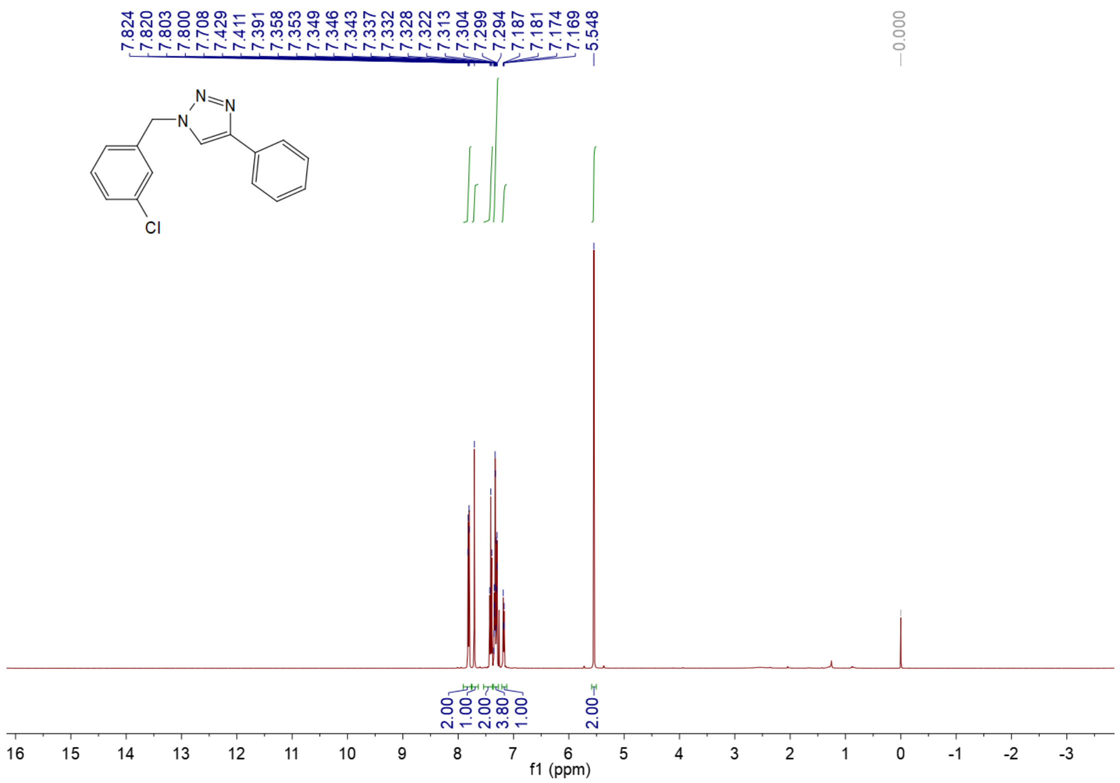

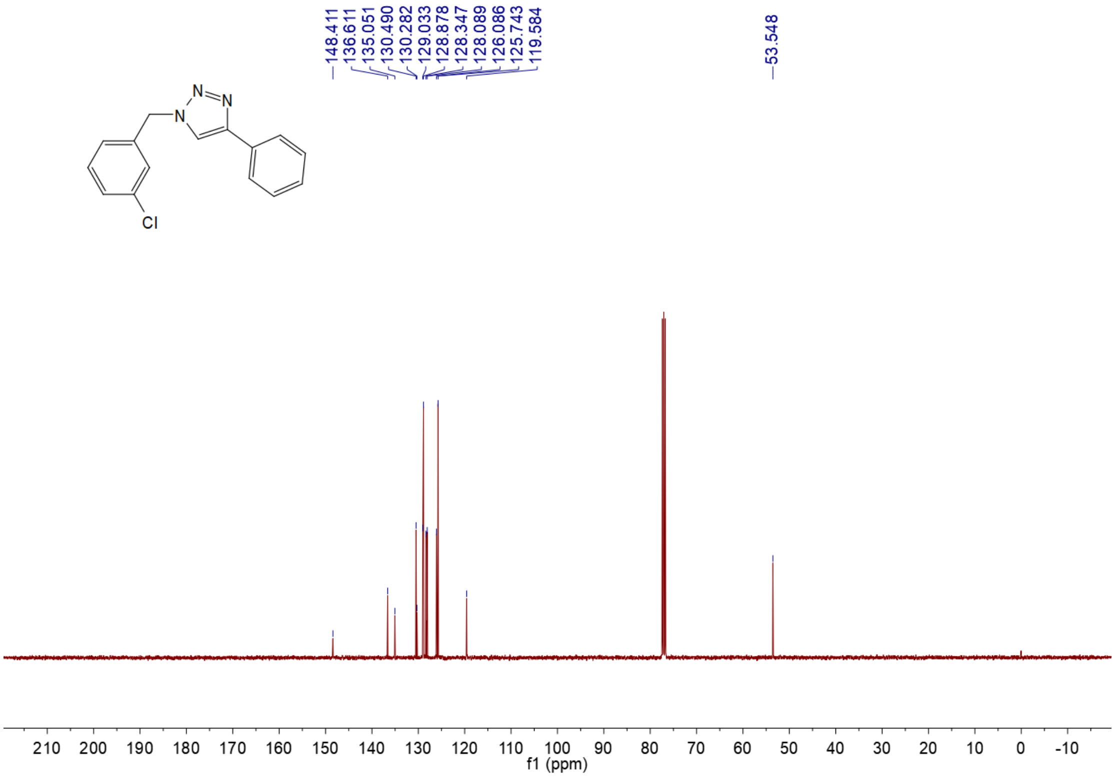

1-(3-Bromobenzyl)-4-phenyl-1H-1,2,3-triazole (4ac)

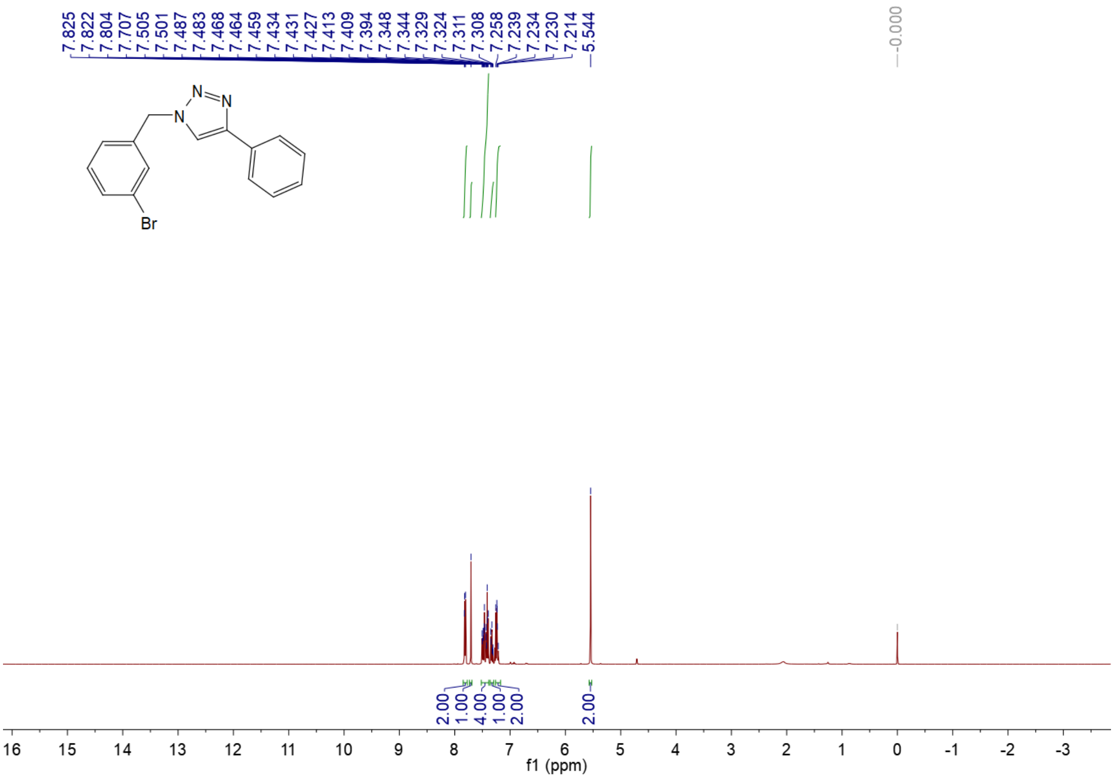

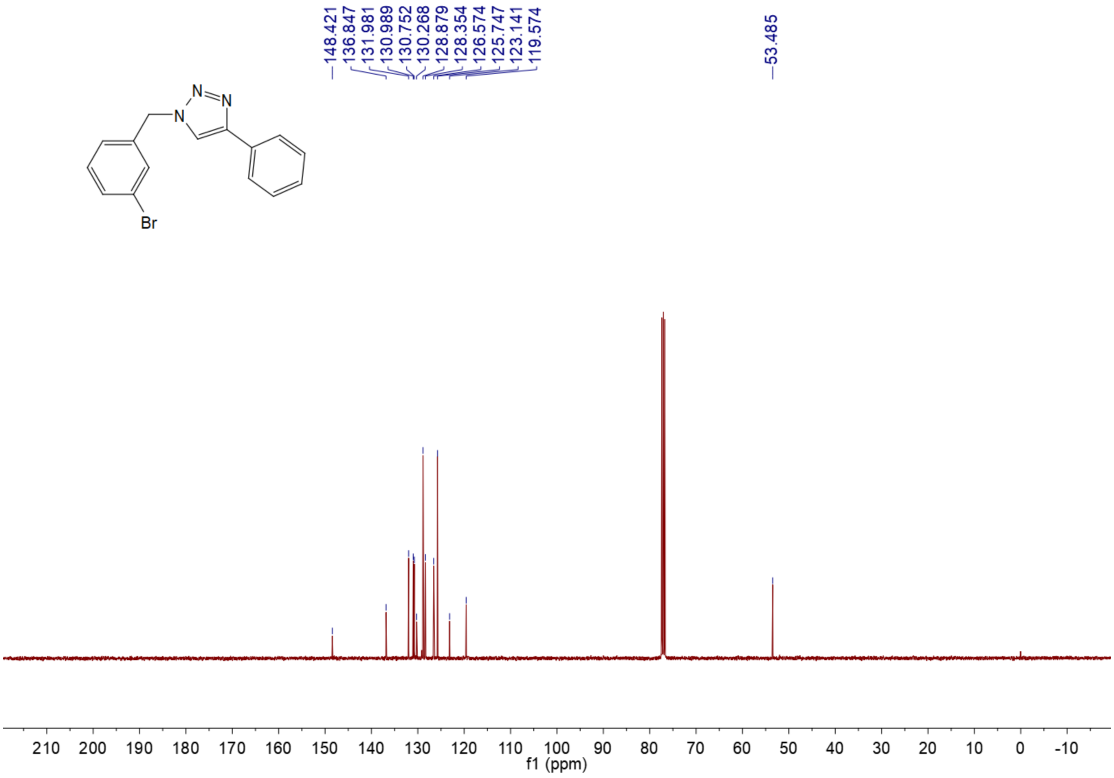

1-(3,4-Dichlorobenzyl)-4-phenyl-1*H*-1,2,3-triazole (4ad)

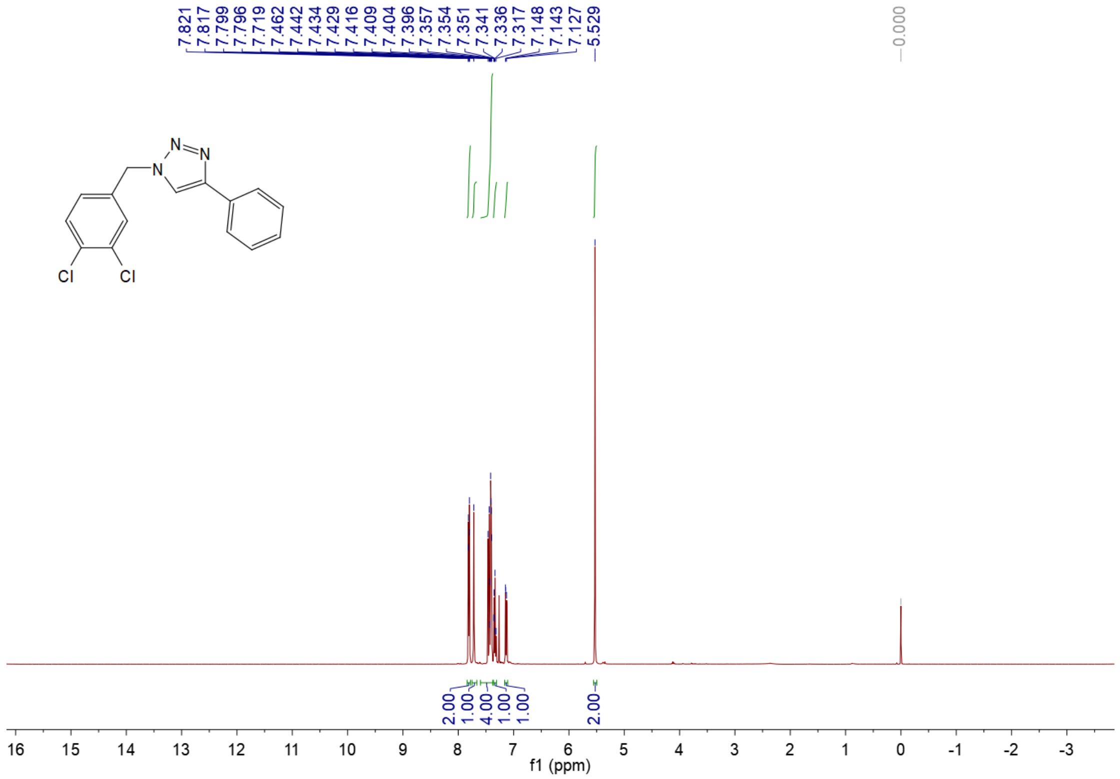

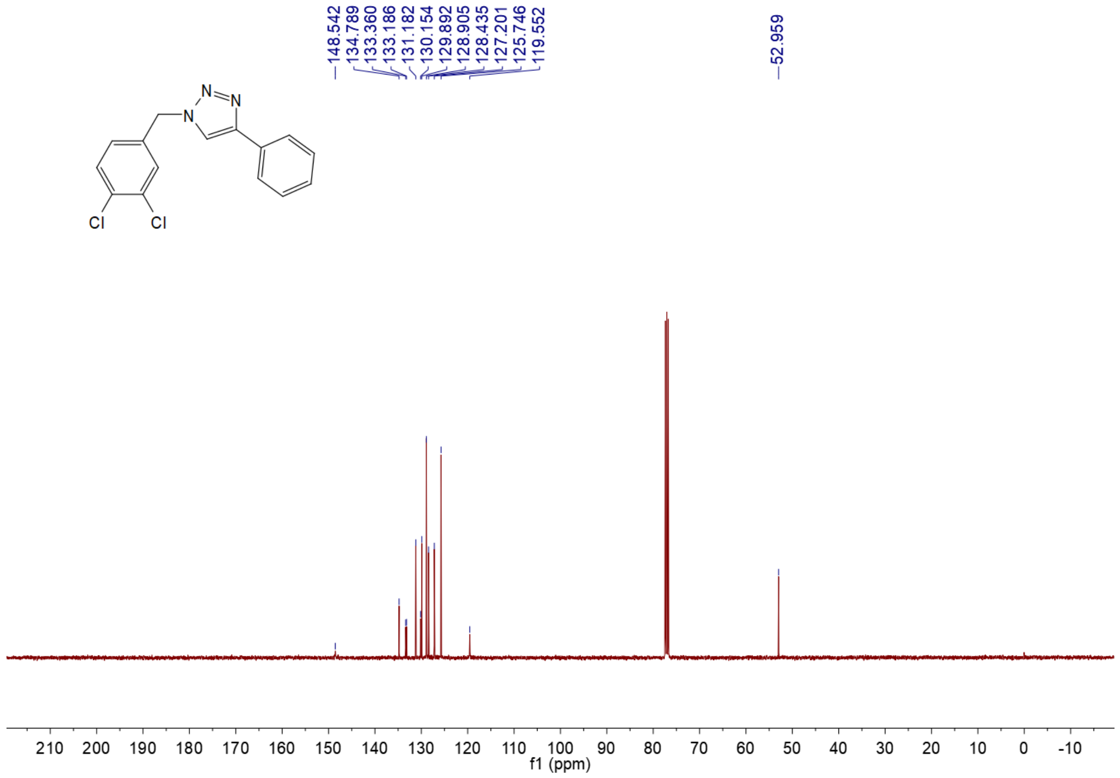

1-(3-Chloro-4-fluorobenzyl)-4-phenyl-1*H*-1,2,3-triazole (4ae)

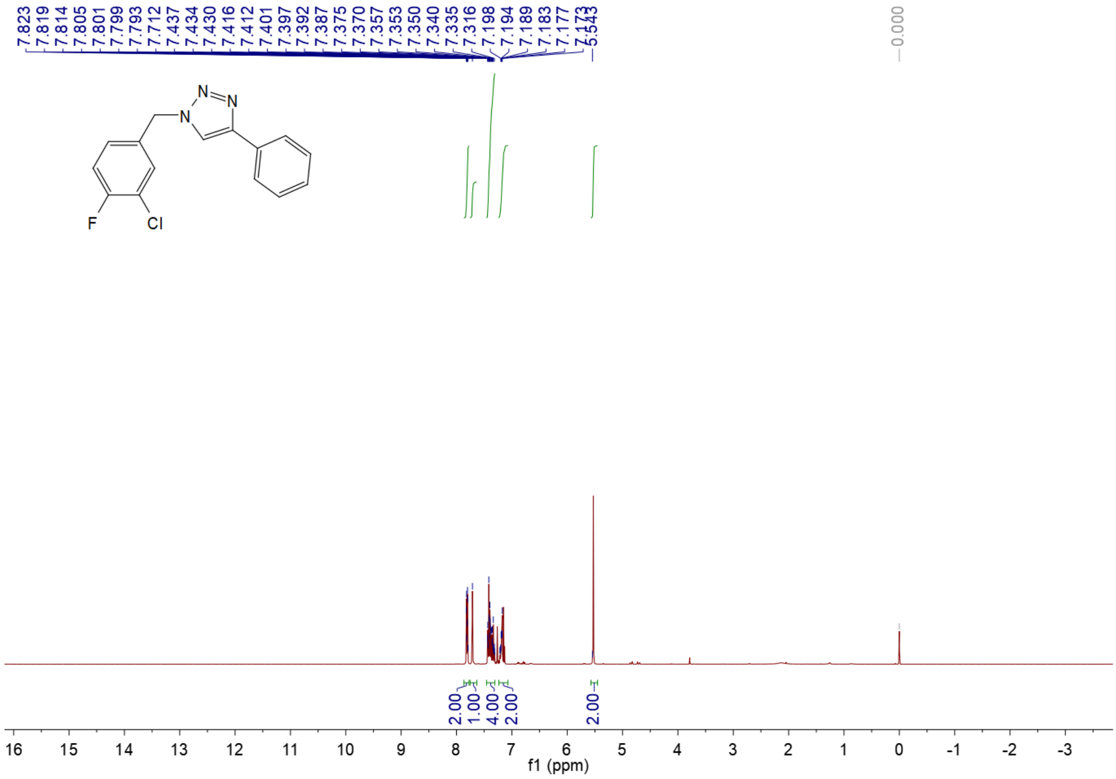

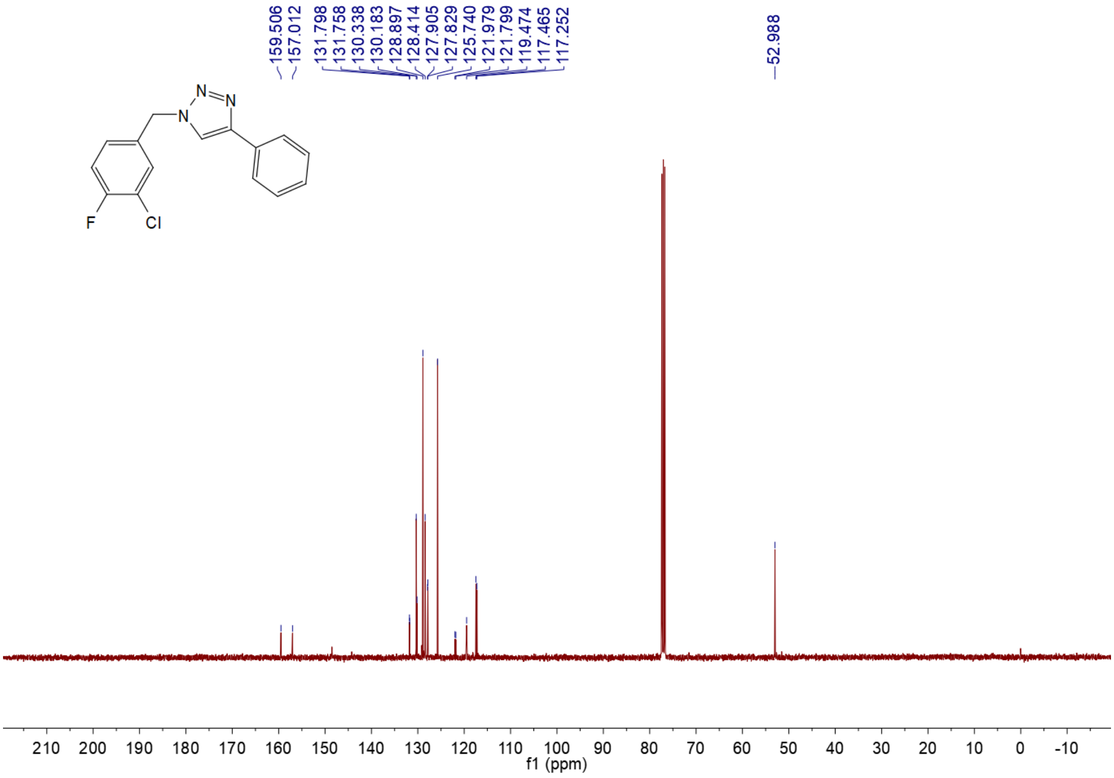

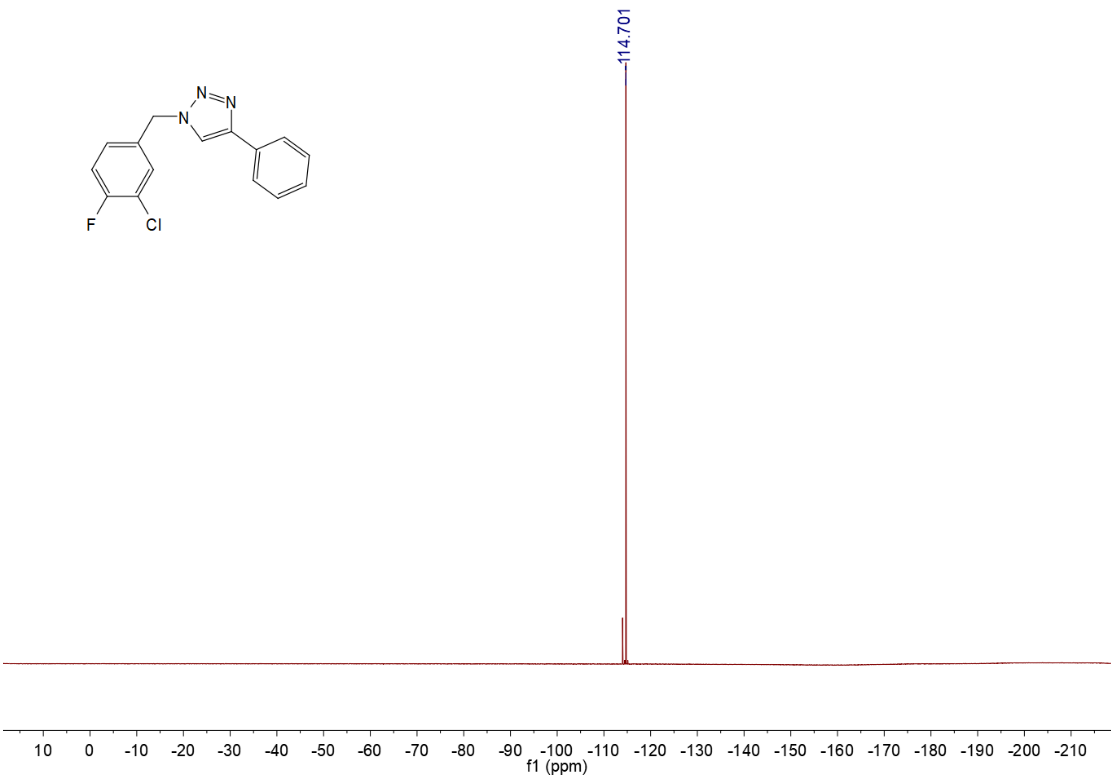

N
N
N
F
Cl
-114.701
10 0 -10 -20 -30 -40 -50 -60 -70 -80 -90 -100 -110 -120 -130 -140 -150 -160 -170 -180 -190 -200 -210
f1 (ppm)

(8R,9S,10R,13S,14S,17S)-17-(1-benzyl-1H-1,2,3-triazol-4-yl)-17-hydroxy-10,13-dimethyl-1,2,6,7,8,9,10,11,12,13,14,15,16,17-tetradecahydro-3H-cyclopenta[a]phenanthren-3-one (5a)

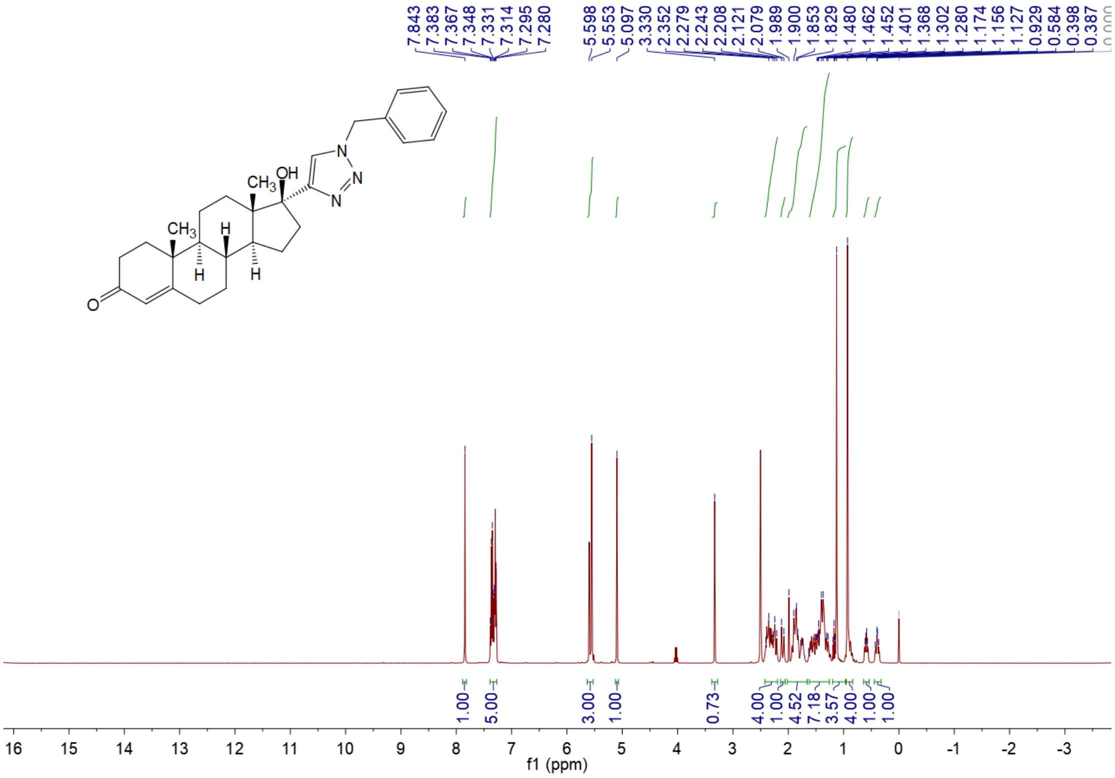

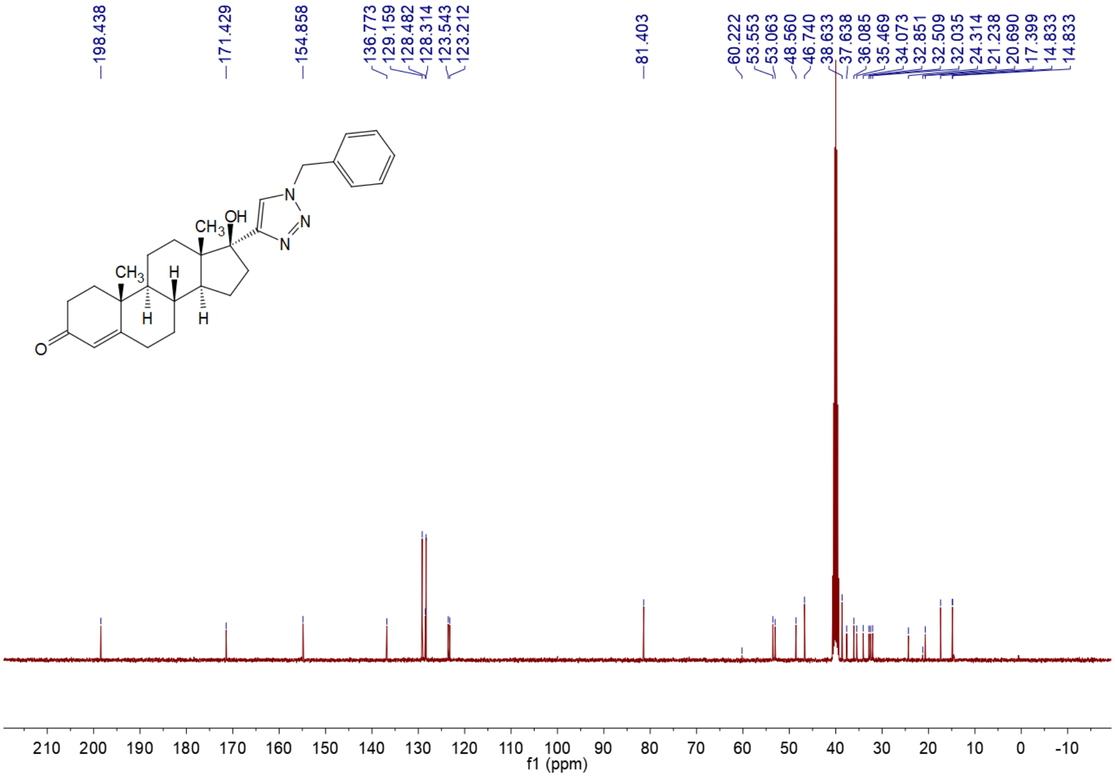

1-((2R,4S,5S)-5-(hydroxymethyl)-4-(4-phenyl-1H-1,2,3-triazol-1-yl)tetrahydrofuran-2-yl)-5-methylpyrimidine-2,4(1H,3H)-dione (5b)

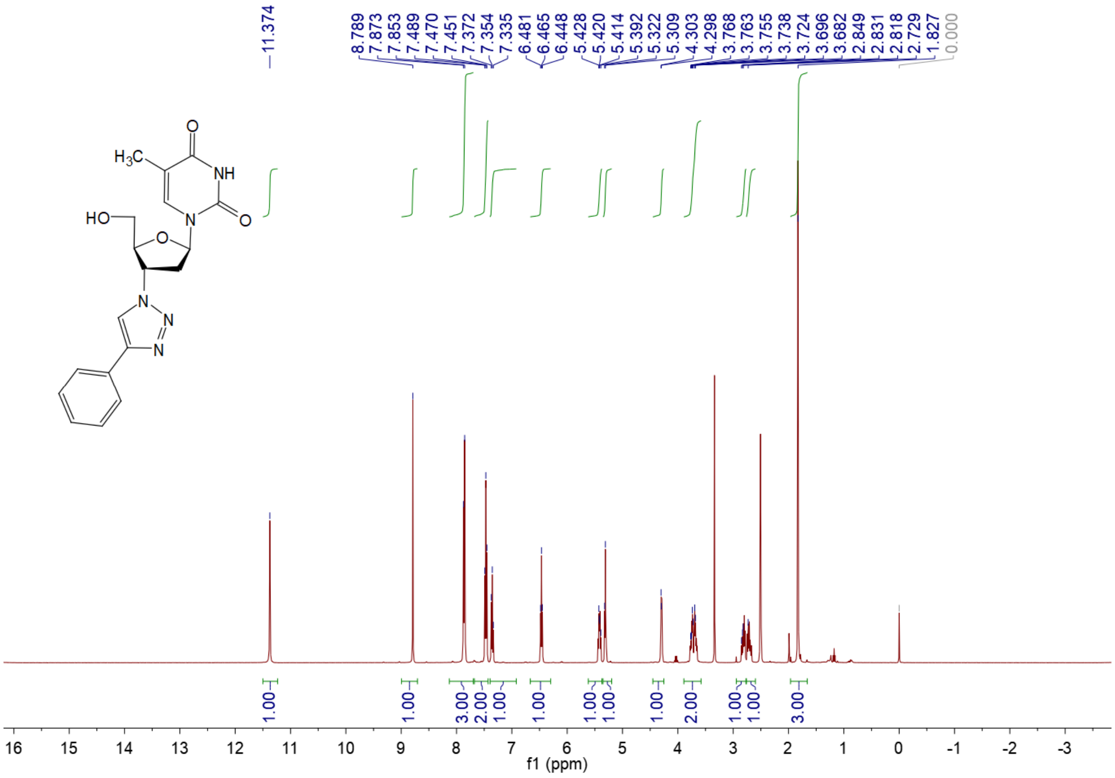

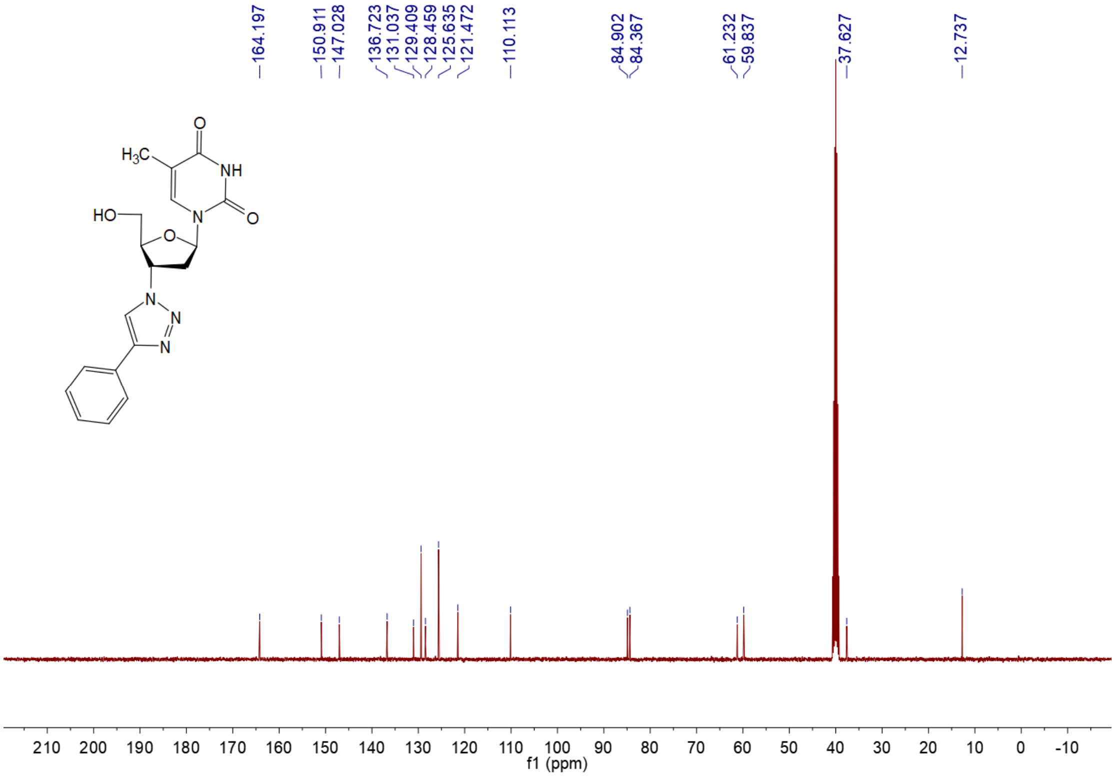

Supplement: Supplementary file 1 [file nanomaterials-12-01070-s001.zip › nanomaterials-1644757-supplementary.pdf]
